# Supplementary material for: Effectiveness and Efficacy of Long-Lasting Insecticidal Nets for Malaria Control in Africa: Systematic Review and Meta-Analysis of Randomized Controlled Trials
Source: Int J Environ Res Public Health. 2025 Jun 30;22(7):1045. doi: 10.3390/ijerph22071045 (PMC12294781; doi:10.3390/ijerph22071045)
Supplement: Supplementary file 1 [file ijerph-22-01045-s001.zip › File S1. Supplementary File searching 1.pdf]

Search	Query	Results	Time
#21	Search: **((((((((((("Randomized Controlled Trials as Topic"[Mesh] OR "Randomized Controlled Trial" [Publication Type] OR "Controlled Clinical Trials as Topic"[Mesh] OR "Intention to Treat Analysis"[Mesh]) AND ("Cost-Effectiveness Analysis"[Mesh] OR "Comparative Effectiveness Research"[Mesh] OR "Treatment Outcome"[Mesh] OR "Evaluation Studies as Topic"[Mesh] OR "Pragmatic Clinical Trial" [Publication Type] OR "Evaluation Study" [Publication Type])) OR ("Treatment Outcome"[Mesh])) OR (("Treatment Outcome"[Mesh]) OR ("Collective Efficacy"[Mesh] OR "Vaccine Efficacy"[Mesh]))) AND ("pyriproxyfen" [Supplementary Concept])) OR ("chlorfenapyr" [Supplementary Concept])) OR ("Piperonyl Butoxide"[Mesh] OR "esdepallethin - piperonyl butoxide" [Supplementary Concept])) AND ("Insecticide-Treated Bednets"[Mesh] OR "Mosquito Nets"[Mesh])) AND ("Pyrethrins"[Mesh])) AND ("Malaria"[Mesh])) AND ("prevention and control" [Subheading]))**	39	00:51:30
#20	Search: **((((((((((("Randomized Controlled Trials as Topic"[Mesh] OR "Randomized Controlled Trial" [Publication Type] OR "Controlled Clinical Trials as Topic"[Mesh] OR "Intention to Treat Analysis"[Mesh]) AND ("Cost-Effectiveness Analysis"[Mesh] OR "Comparative Effectiveness Research"[Mesh] OR "Treatment Outcome"[Mesh] OR "Evaluation Studies as Topic"[Mesh] OR "Pragmatic Clinical Trial" [Publication Type] OR "Evaluation Study" [Publication Type])) OR ("Treatment Outcome"[Mesh])) OR (("Treatment Outcome"[Mesh]) OR ("Collective Efficacy"[Mesh] OR "Vaccine Efficacy"[Mesh]))) AND ("pyriproxyfen" [Supplementary Concept])) OR ("chlorfenapyr" [Supplementary Concept])) OR ("Piperonyl Butoxide"[Mesh] OR "esdepallethin - piperonyl butoxide" [Supplementary Concept])) AND ("Insecticide-Treated Bednets"[Mesh] OR "Mosquito Nets"[Mesh])) AND ("Pyrethrins"[Mesh])) AND ("Malaria"[Mesh])) AND ("prevention and control" [Subheading])) AND ("Africa South of the Sahara"[Mesh])**	31	00:50:04
#19	Search: **"Africa South of the Sahara"[Mesh]** Sort by: **Most Recent**	265,897	00:47:36
#18	Search: **"prevention and control" [Subheading]** Sort by: **Most Recent**	1,479,005	00:45:25
#17	Search: **"Malaria"[Mesh]** Sort by: **Most Recent**	75,742	00:43:19
#16	Search: **"Pyrethrins"[Mesh]** Sort by: **Most Recent**	11,392	00:42:27
#15	Search: **"Insecticide-Treated Bednets"[Mesh] OR "Mosquito Nets"[Mesh]** Sort by: **Most Recent**	1,885	00:41:21

Search	Query	Results	Time
#14	Search: **"Insecticide-Treated Bednets"[Mesh]** Sort by: **Most Recent**	1,581	00:40:30
#13	Search: **"Piperonyl Butoxide"[Mesh] OR "esdepallethin - piperonyl butoxide" [Supplementary Concept]** Sort by: **Most Recent**	822	00:35:16
#12	Search: **"chlorfenapyr" [Supplementary Concept]** Sort by: **Most Recent**	162	00:34:26
#11	Search: **"pyriproxyfen" [Supplementary Concept]** Sort by: **Most Recent**	446	00:33:39
#10	Search: **("Treatment Outcome"[Mesh]) OR ("Collective Efficacy"[Mesh] OR "Vaccine Efficacy"[Mesh])** Sort by: **Most Recent**	1,270,886	00:32:35
#9	Search: **"Treatment Outcome"[Mesh]** Sort by: **Most Recent**	1,270,024	00:28:46
#8	Search: **"Cost-Effectiveness Analysis"[Mesh] OR "Comparative Effectiveness Research"[Mesh] OR "Treatment Outcome"[Mesh] OR "Evaluation Studies as Topic"[Mesh] OR "Pragmatic Clinical Trial" [Publication Type] OR "Evaluation Study" [Publication Type]** Sort by: **Most Recent**	2,609,569	00:27:04
#7	Search: **"Randomized Controlled Trials as Topic"[Mesh] OR "Randomized Controlled Trial" [Publication Type] OR "Controlled Clinical Trials as Topic"[Mesh] OR "Intention to Treat Analysis"[Mesh]** Sort by: **Most Recent**	778,719	00:24:16
#5	Search: **Similar articles for PMID: 37776879** Filters: **Full text, Humans, English, from 2000/1/1 - 2024/2/24**	71	00:18:02
#6	Search: **Similar articles for PMID: 37776879** Filters: **Full text, Associated data, Humans, English, from 2000/1/1 - 2024/2/24**	41	00:17:43
#4	Search: **Similar articles for PMID: 37776879** Filters: **Full text, in the last 5 years, Humans, English**	43	00:15:04
#3	Search: **Similar articles for PMID: 37776879** Filters: **Full text, in the last 5 years, Humans, English**	43	00:14:19
#2	Search: **Similar articles for PMID: 37776879** Filters: **Full text, Associated data, in the last 5 years, Humans, English**	16	00:13:02

Search	Query	Results	Time
#1	Search: **randomized controlled trials on Effectiveness and Efficacy of pyriproxyfen, chlorfenapyr, and piperonyl butoxide long-lasting insecticidal nets (LLINs) with pyrethroid-only LLINs for malaria control** Sort by: **Most Recent**	1	00:12:29
