# Supplementary material for: Effectiveness and Efficacy of Long-Lasting Insecticidal Nets for Malaria Control in Africa: Systematic Review and Meta-Analysis of Randomized Controlled Trials
Source: Int J Environ Res Public Health. 2025 Jun 30;22(7):1045. doi: 10.3390/ijerph22071045 (PMC12294781; doi:10.3390/ijerph22071045)
Supplement: Supplementary file 1 [file ijerph-22-01045-s001.zip › File S3. Supplementary Result Figures S1-S106.pdf]

Supplementary Result Figure S

Supplementary Figure = "Figure S "

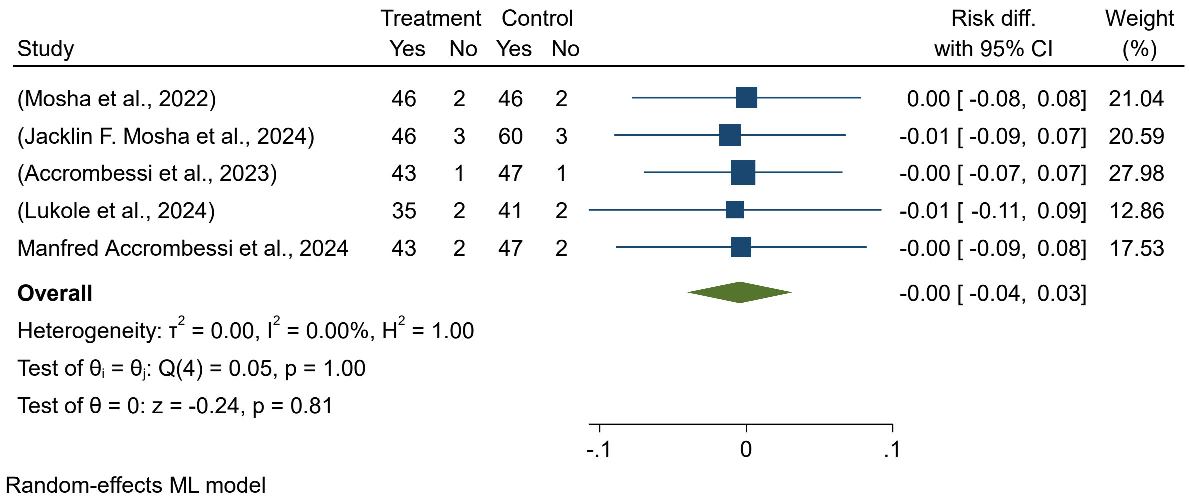

Figure S1: Illustrates the effectiveness and efficacy of pyriproxyfen long-lasting insecticidal nets (LLINs) in malaria infection risk reduction among children compared to pyrethroid-only LLINs for malaria control in Africa 2024.

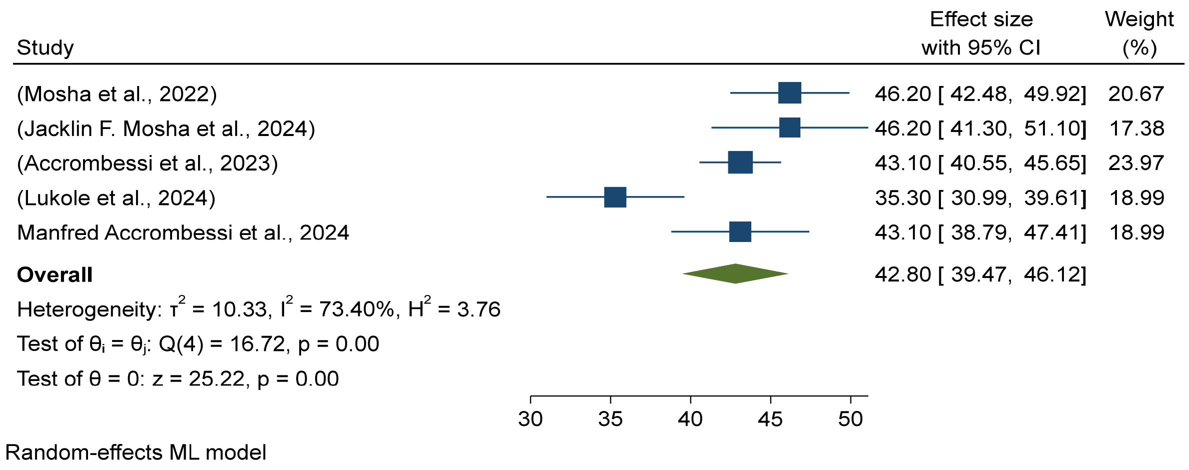

Figure S2: Forest plots showed pooled prevalence of malaria among children using pyriproxyfen long-lasting insecticidal nets (LLINs) for malaria control in Africa in 2024.

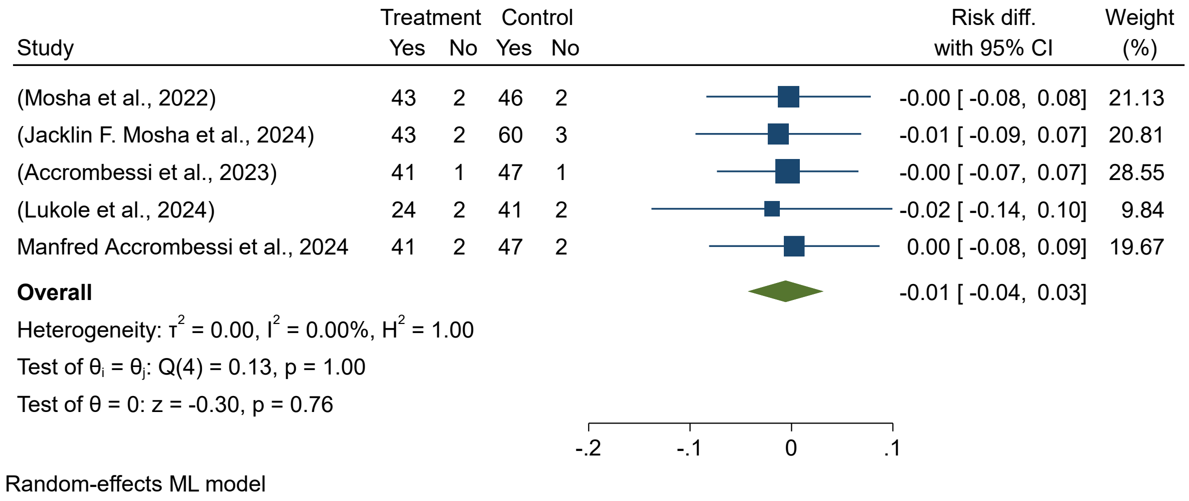

Figure S3: illustrates the effectiveness and efficacy of chlorfenapyr long-lasting insecticidal nets (LLINs) in malaria infection risk reduction among children compared to pyrethroid-only LLINs for malaria control in Africa 2024.

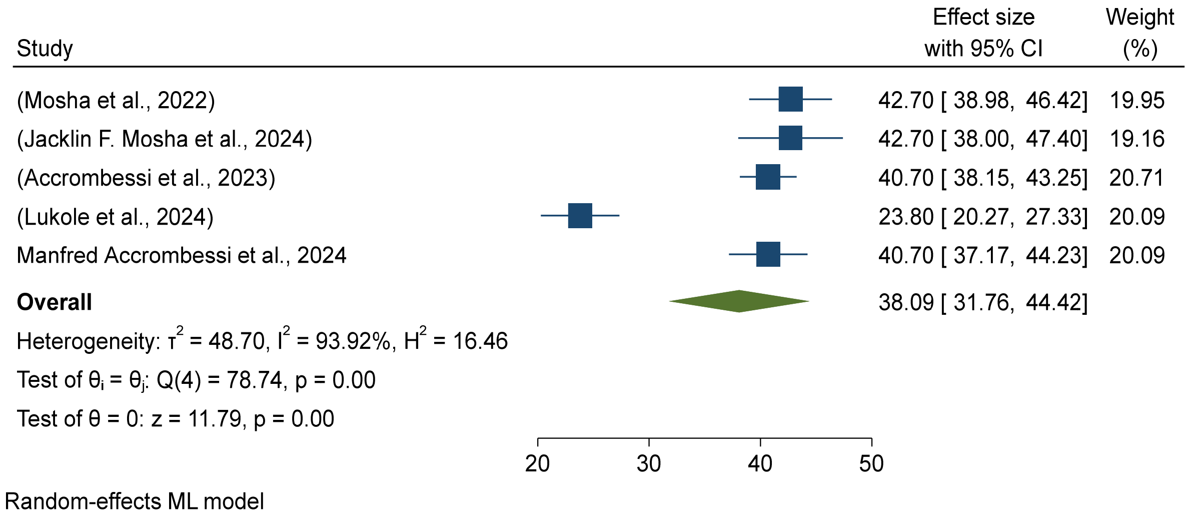

Figure S4: Forest plots showed pooled prevalence of malaria infection among children using chlorfenapyr long-lasting insecticidal nets (LLINs) for malaria control in Africa 2024.

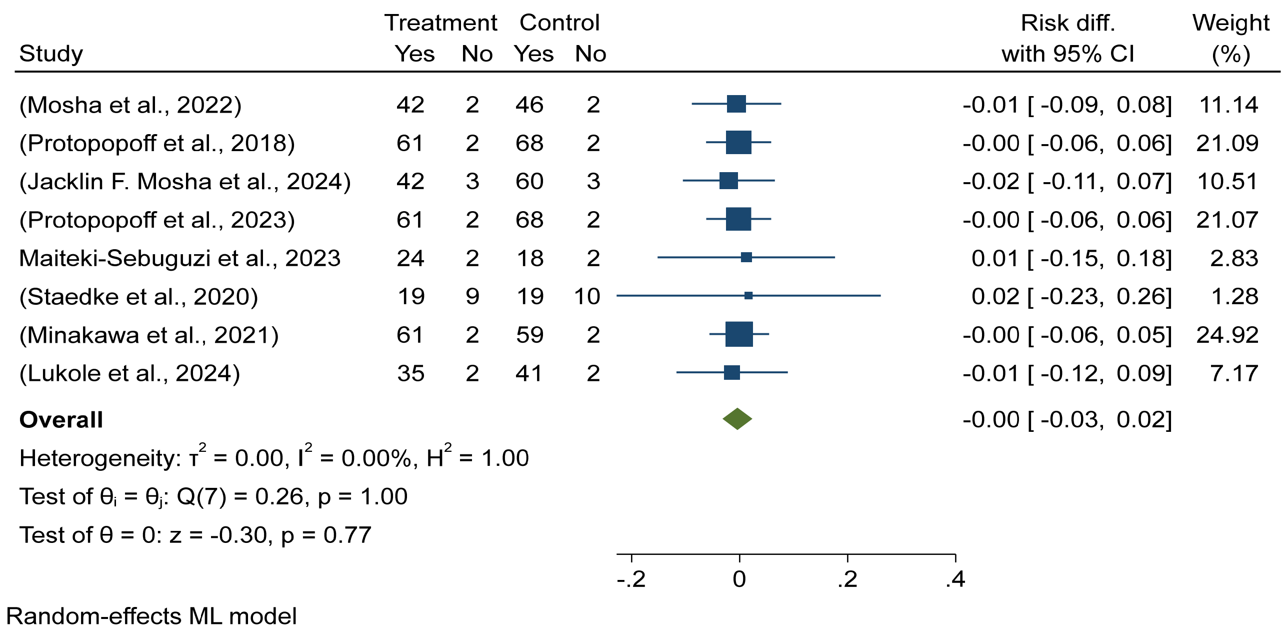

Figure S5: Forest plot illustrates the effectiveness and efficacy of Piperonyl butoxide long-lasting insecticidal nets (LLINs) in malaria infection risk reduction among children compared to pyrethroid-only LLINs for malaria control in Africa 2024

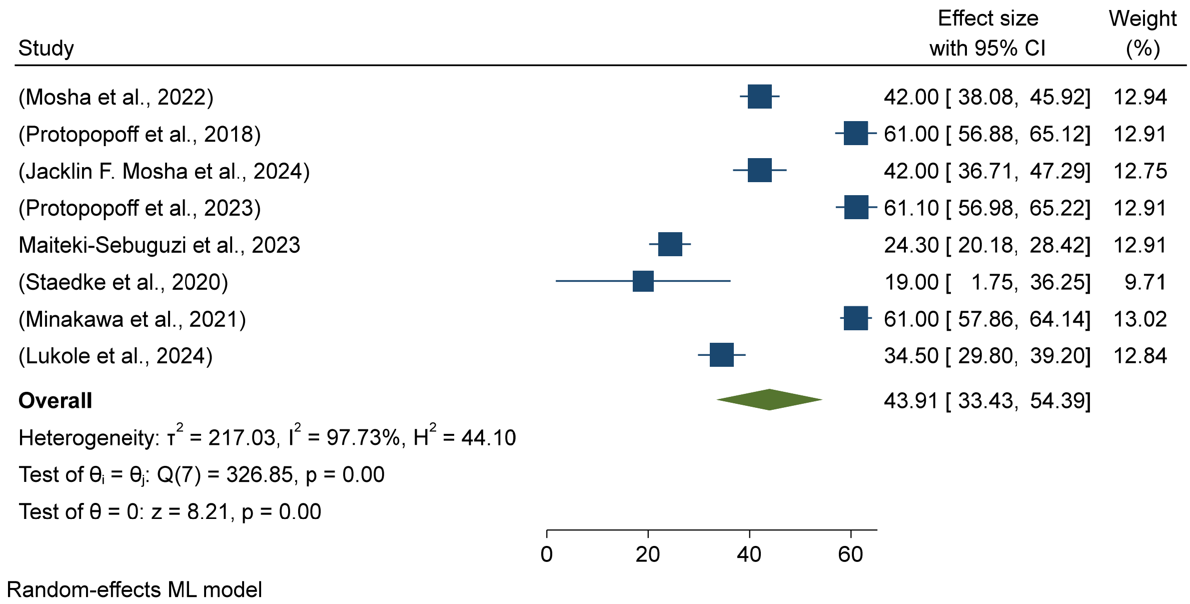

Figure S6: Forest plots showed pooled prevalence of malaria infection among children using c Piperonyl butoxide long-lasting insecticidal nets (LLINs) for malaria control in Africa 2024.

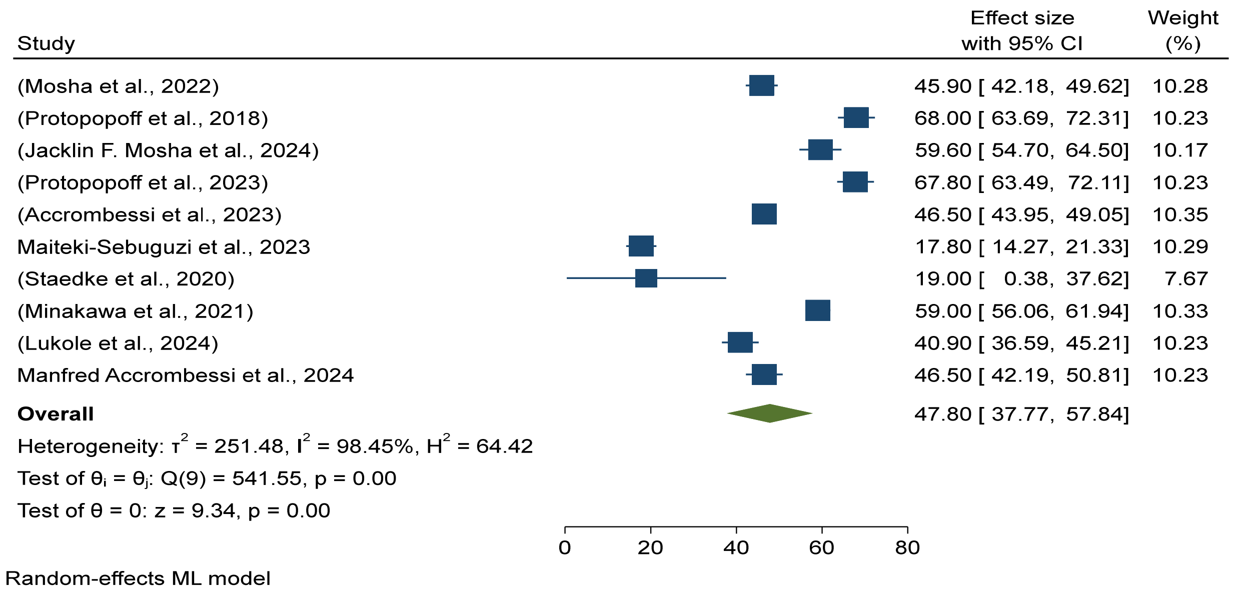

Figure S7: Forest plots showed pooled prevalence of malaria infection among children using pyrethroid-only long-lasting insecticidal nets (LLINs) for malaria control in Africa 2024

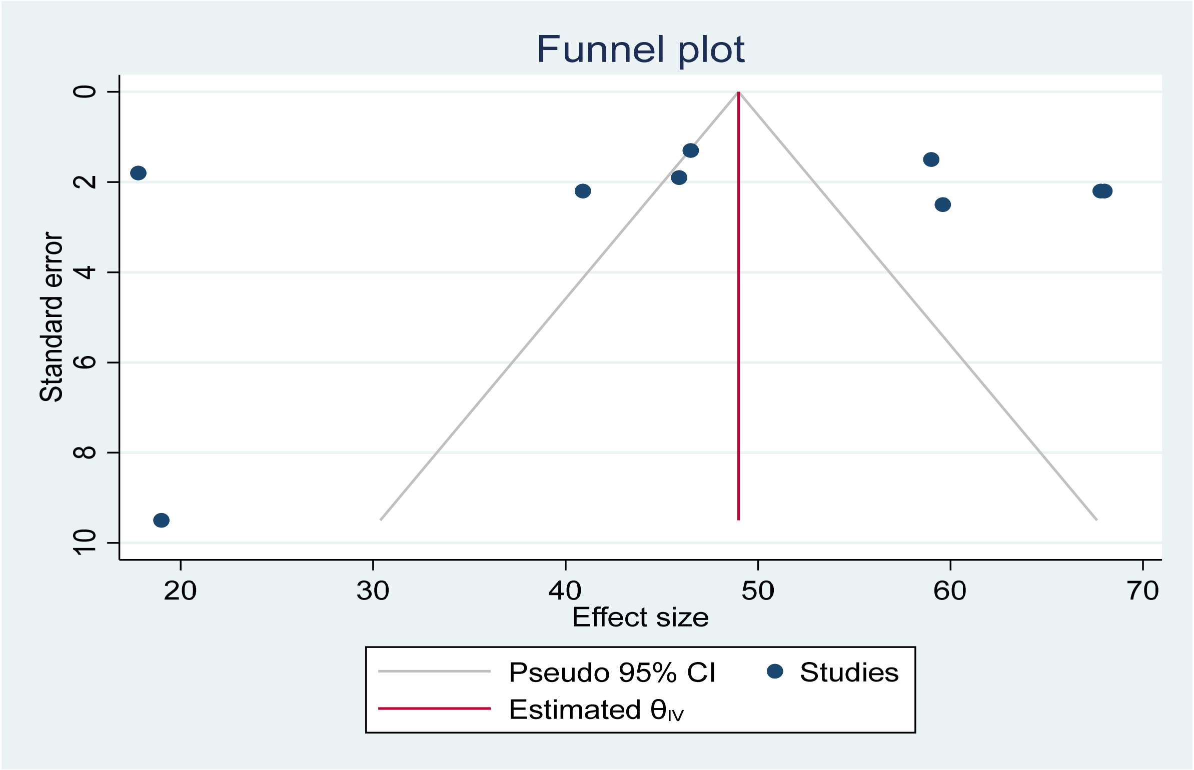

Figure S8**:** Funnel plot showing the distribution of included studies pooled malaria infection risk reduction among children using pyrethroid-only long-lasting insecticidal nets (LLINs) for malaria control in Africa in 2024.

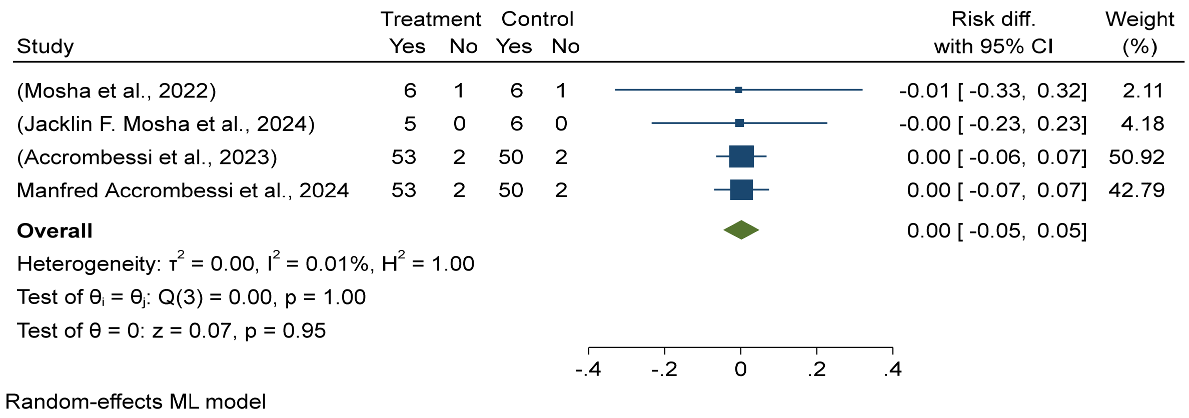

Figure S9: illustrates the effectiveness and efficacy of pyriproxyfen long-lasting insecticidal nets (LLINs) with no difference in anaemia risk reduction among children aged 6 months to 4 years compared to pyrethroid-only LLINs in Africa in 2024.

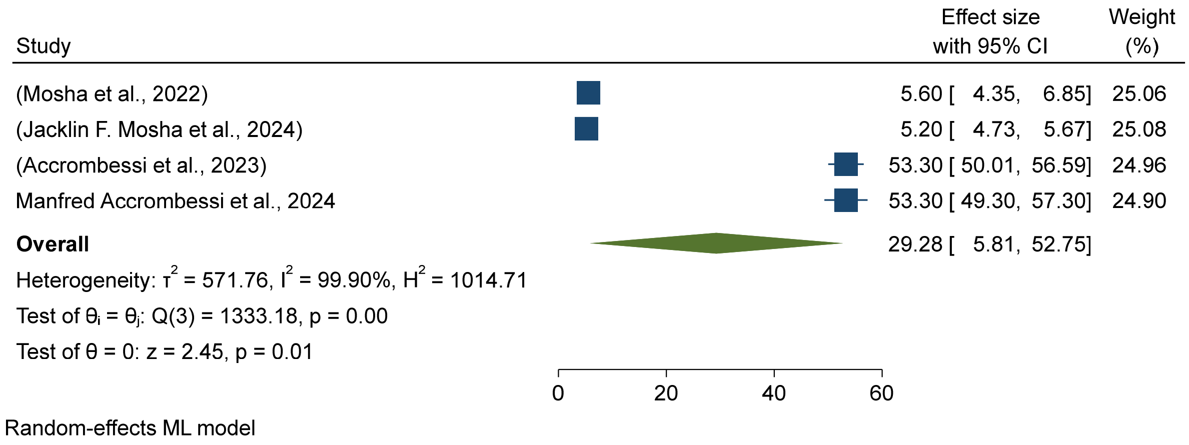

Figure S10: Forest plots showed pooled prevalence of anaemia among children aged 6 months to 4 years using pyriproxyfen long-lasting insecticidal nets (LLINs) for malaria control in Africa in 2024.

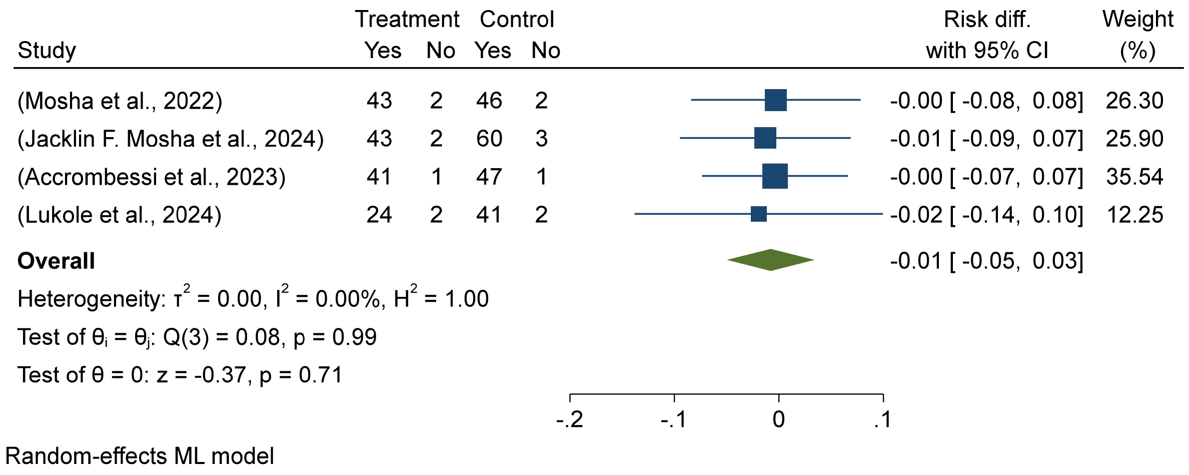

Figure S11: Forest plot shows the effectiveness and efficacy of chlorfenapyr long-lasting insecticidal nets (LLINs) in anaemia risk reduction among children aged 6 months to 4 years compared to pyrethroid-only LLINs in Africa in 2024.

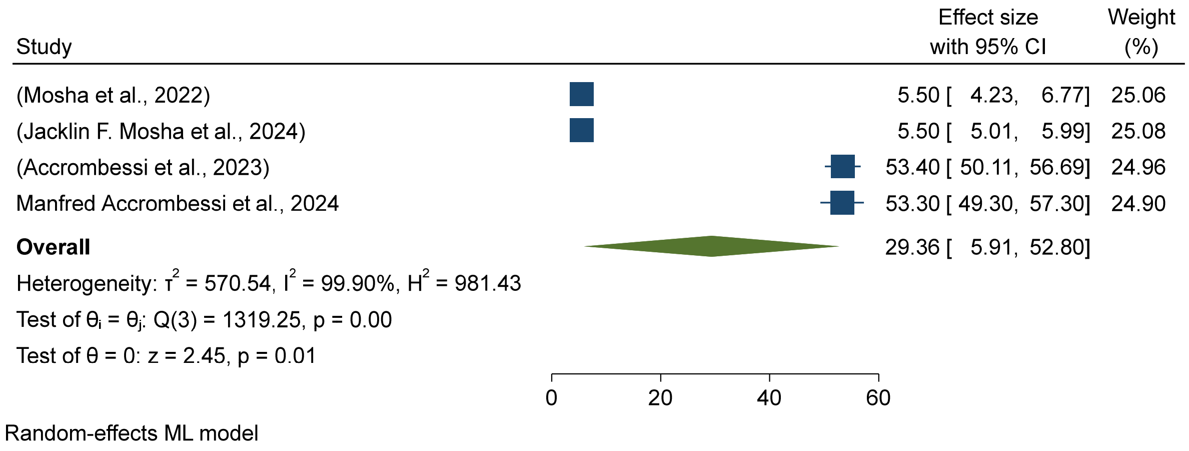

Figure S12: Forest plots showed a pooled prevalence of anaemia among children using chlorfenapyr long-lasting insecticidal nets (LLINs) for malaria control in Africa in 2024.

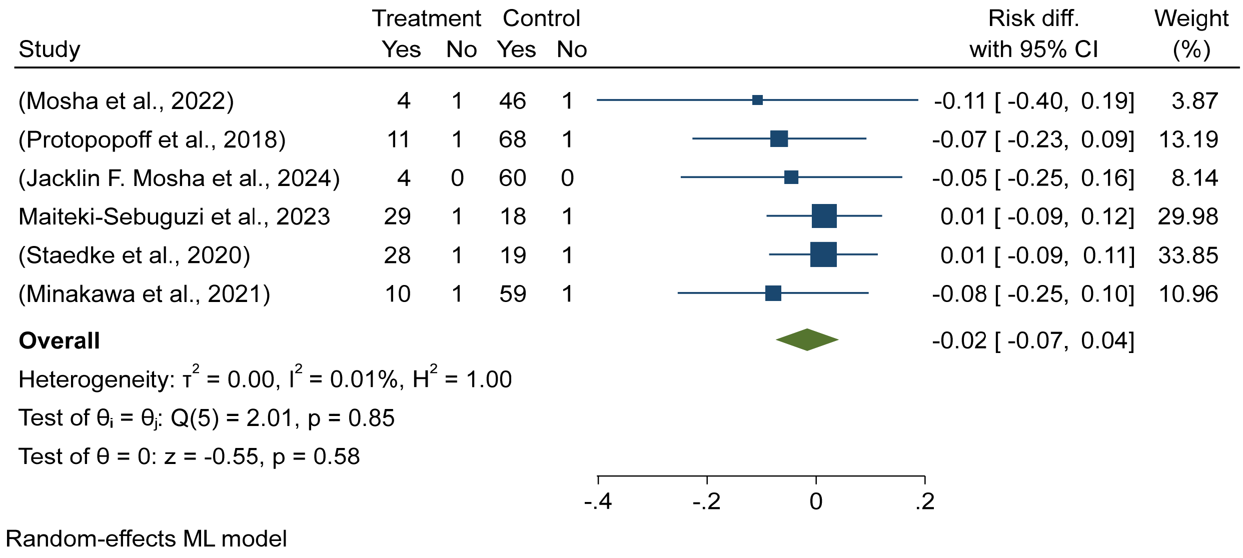

Figure S13: Forest lot shows the effectiveness and efficacy of Piperonyl butoxide long-lasting insecticidal nets (LLINs) in anaemia risk reduction among children aged 6 months to 4 years compared to pyrethroid-only LLINs in Africa in 2024.

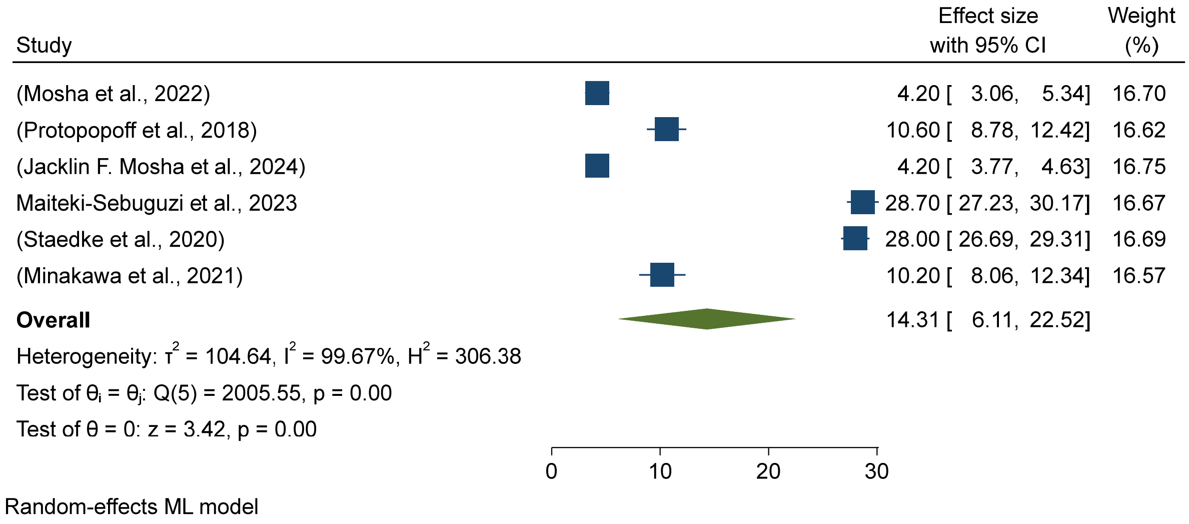

Figure S14: Forest plots showed a pooled prevalence of anaemia among children using piperonyl butoxide long-lasting insecticidal nets (LLINs) for malaria control in Africa in 2024.

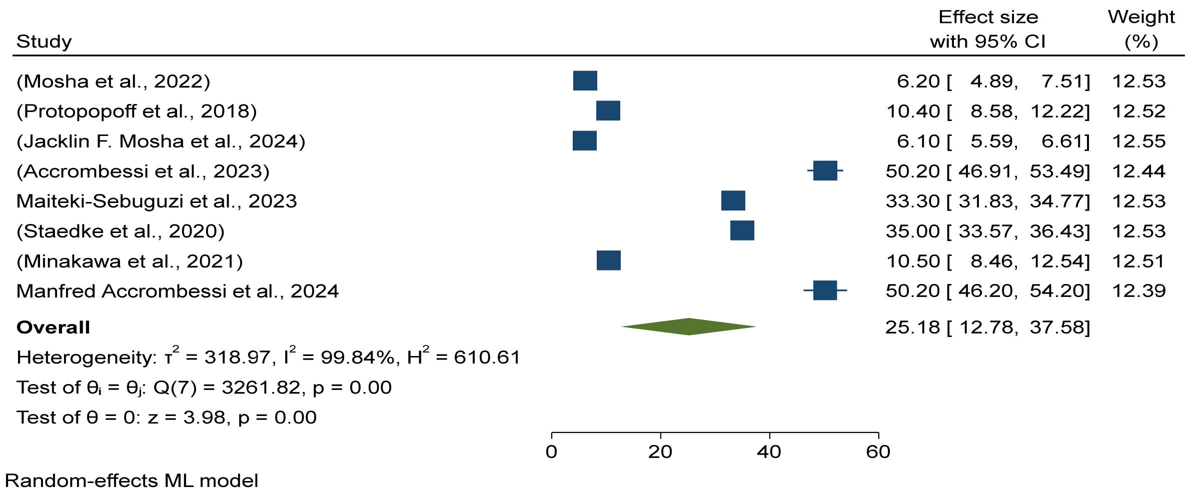

Figure S15: Forest plots showed a pooled prevalence of anaemia among children using pyrethroid-only long lasting insecticidal nets (LLINs) for malaria control in Africa in 2024.

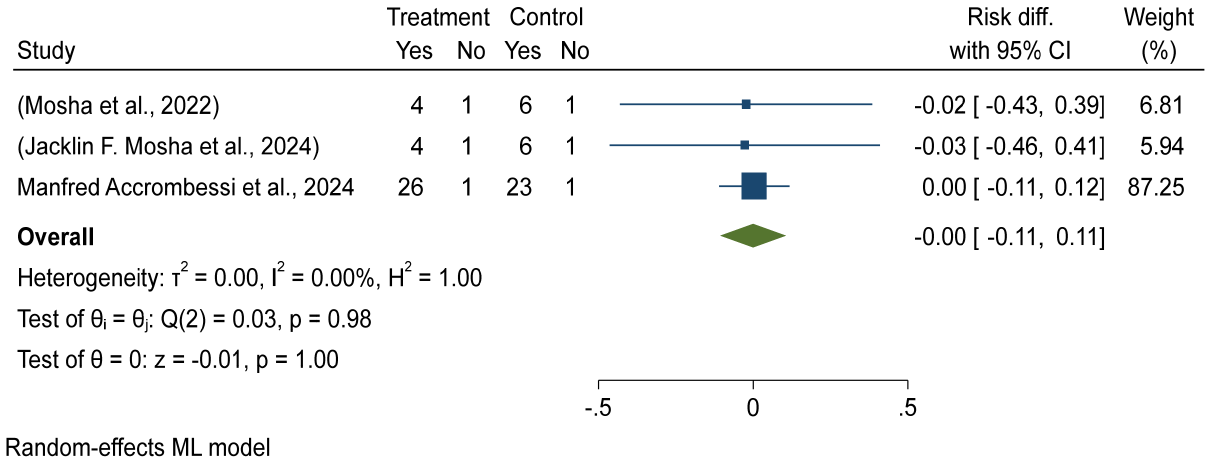

Figure S16: Forest plot shows the effectiveness and efficacy of pyriproxyfen long-lasting insecticidal nets (LLINs) with no difference in reducing mean indoor vector density per household per night compared to pyrethroid-only LLINs in Africa in 2024.

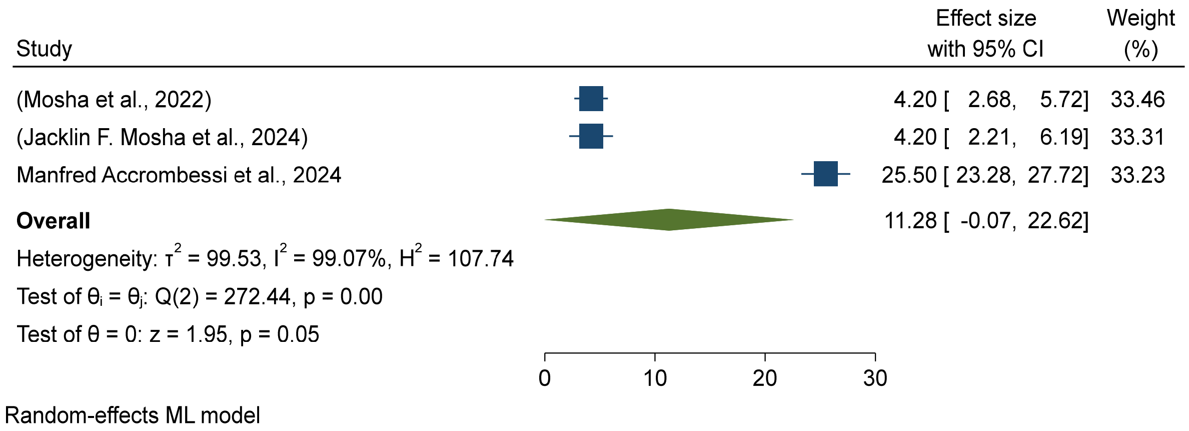

Figure S17: Forest plots showed baseline pooled mean indoor vectors density per household per night using pyriproxyfen long lasting insecticidal nets (LLINs) for malaria control in Africa in 2024.

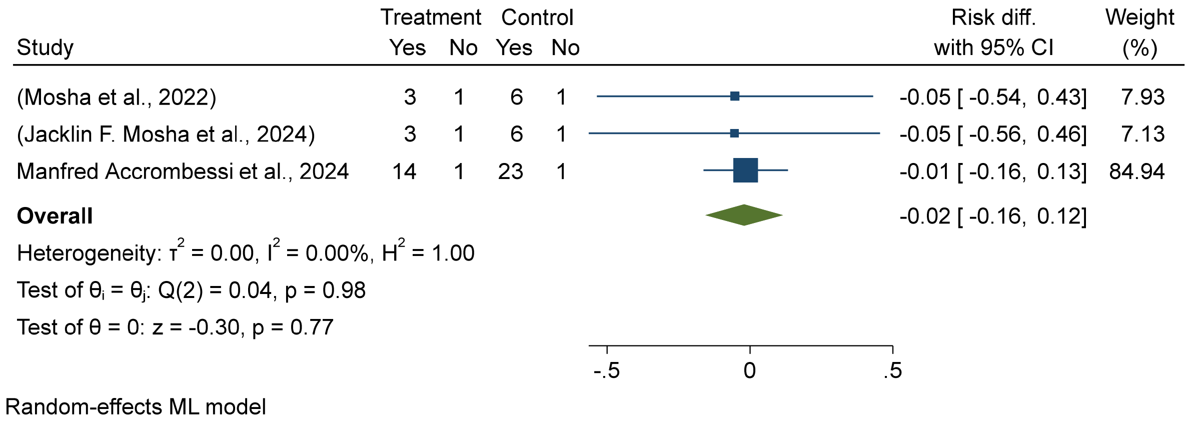

Figure S18: Forest plot shows the effectiveness and efficacy of chlorfenapyr long-lasting insecticidal nets (LLINs) in reducing mean indoor vector density per household per night compared to pyrethroid-only LLINs in Africa in 2024.

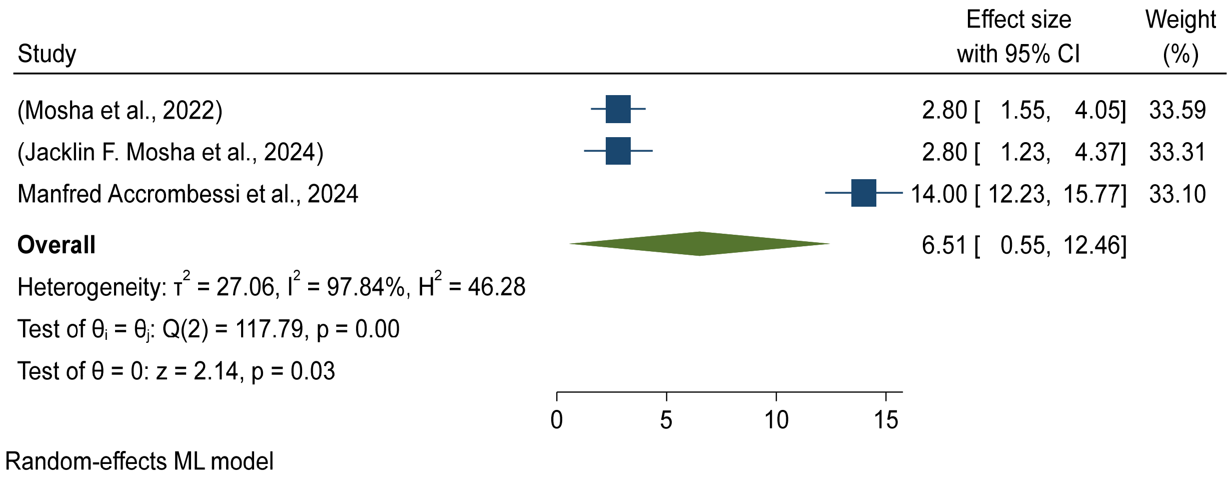

Figure S19: Forest plots showed baseline pooled mean indoor vectors density per household per night using chlorfenapyr LLINs for malaria control in Africa in 2024.

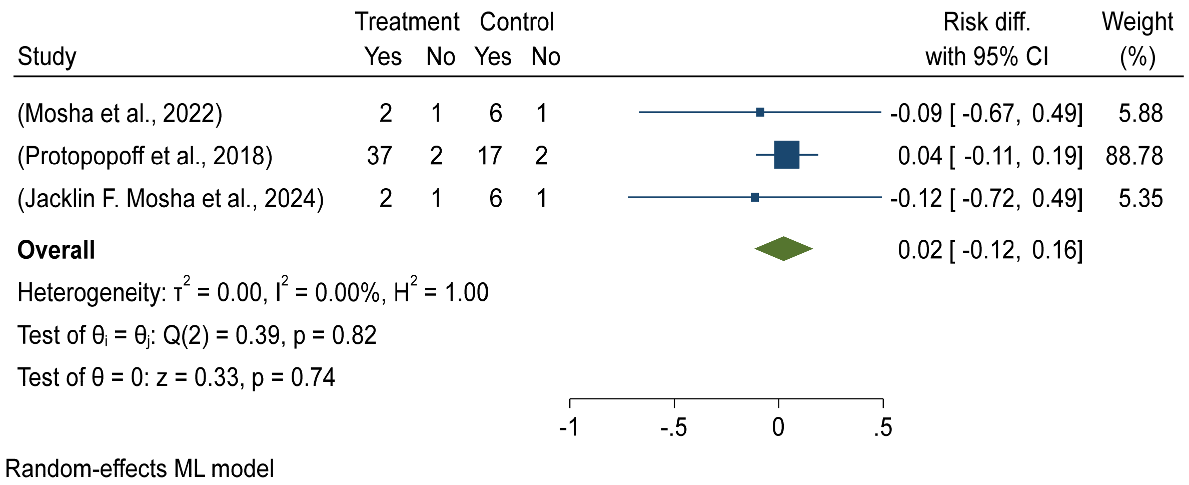

Figure S20: Forest plot shows the effectiveness and efficacy of piperonyl butoxide LLINs in reducing mean indoor vector density per household per night compared to pyrethroid-only LLINs in Africa in 2024

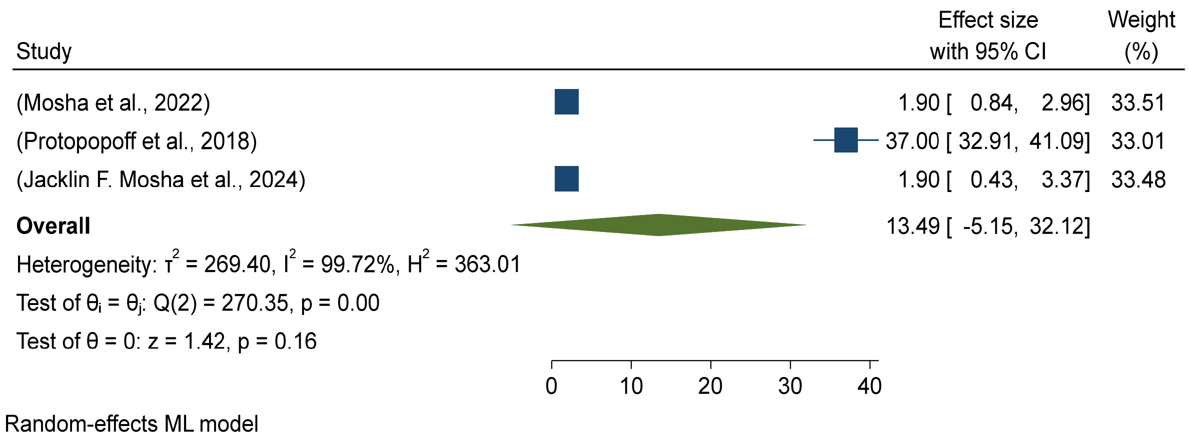

Figure S21: Forest plots showed baseline pooled mean indoor vectors density per household per night using piperonyl butoxide LLINs for malaria control in Africa in 2024.

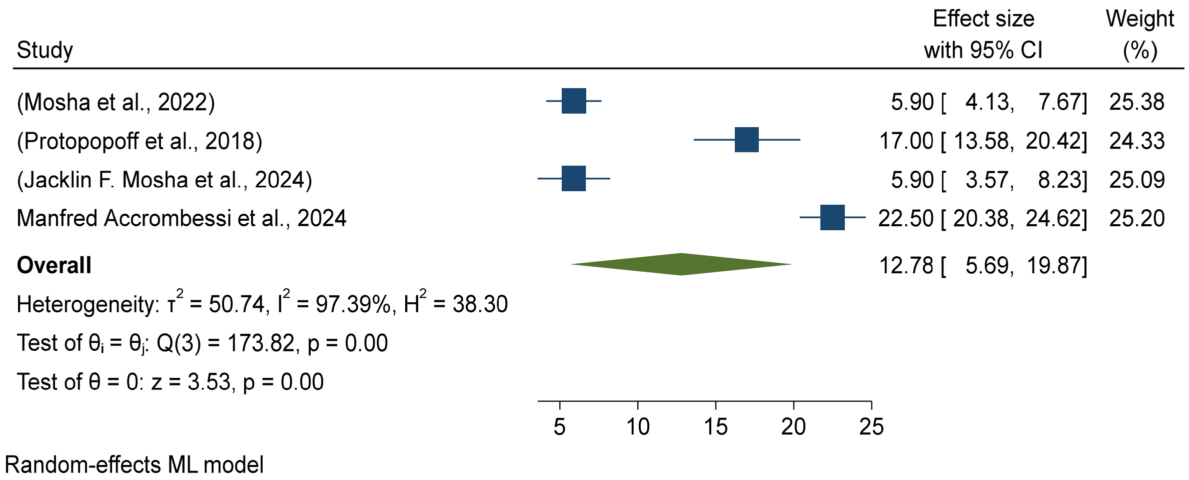

Figure S22: Forest plots showed baseline pooled mean indoor vectors density per household per night using pyrethroid-only LLINs for malaria control in Africa in 2024.

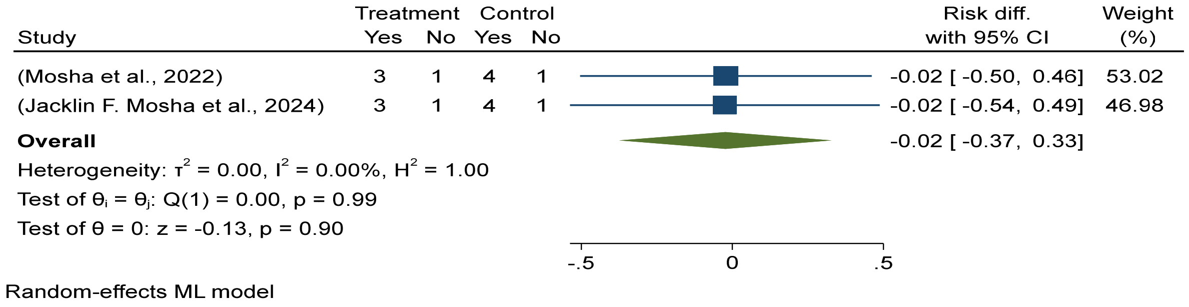

Figure S23: Forest plot shows the effectiveness and efficacy of pyriproxyfen long-lasting insecticidal nets (LLINs) in sporozoite rate reduction compared to pyrethroid-only LLINs in Africa in 2024.

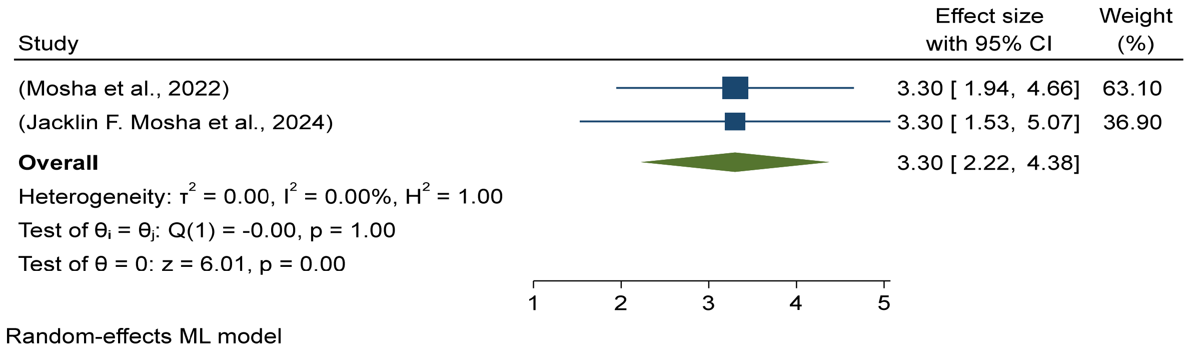

Figure S24: Forest plots showed baseline pooled sporozoite rate among pyriproxyfen LLINs intervention for malaria control in Africa in 2024.

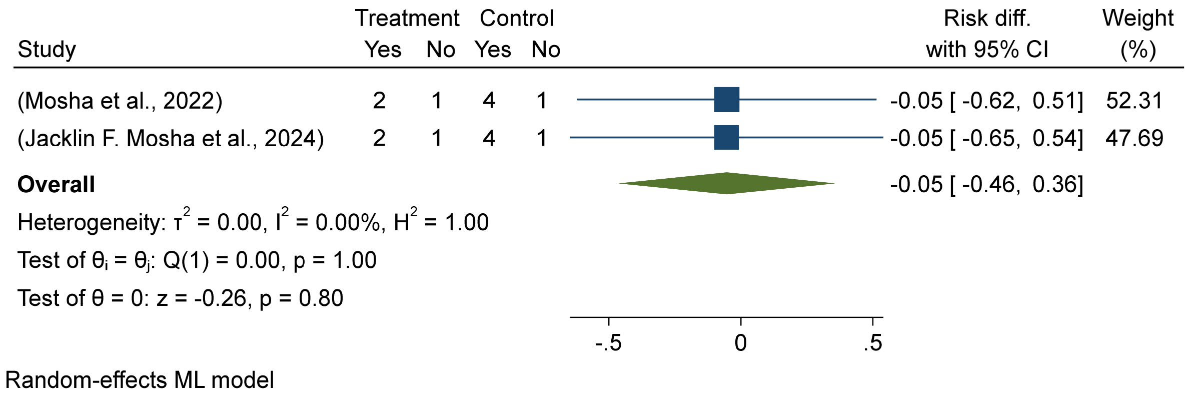

Figure S25: Forest plot shows the effectiveness and efficacy of chlorfenapyr long-lasting insecticidal nets (LLINs) in sporozoite rate reduction compared to pyrethroid-only LLINs in Africa in 2024.

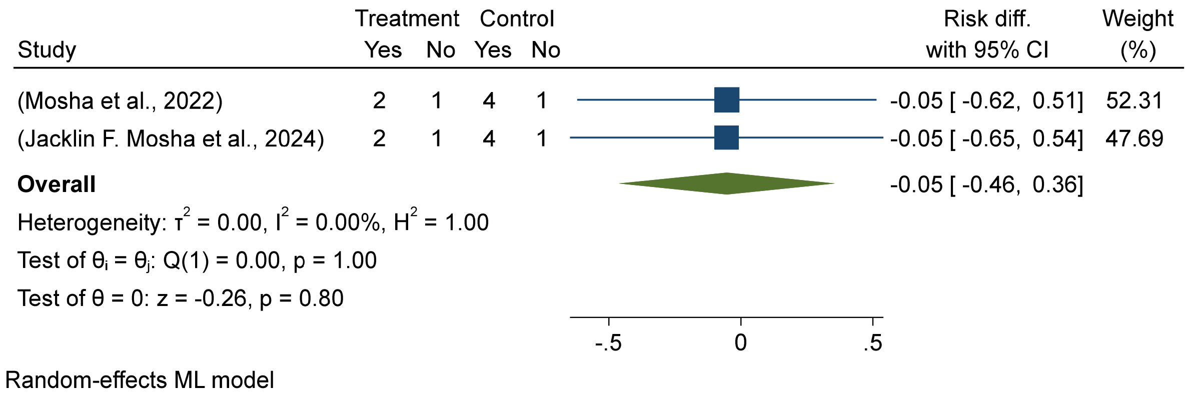

Figure S26: Forest plot shows the effectiveness and efficacy of chlorfenapyr long-lasting insecticidal nets (LLINs) in sporozoite rate reduction compared to pyrethroid-only LLINs in Africa in 2024.

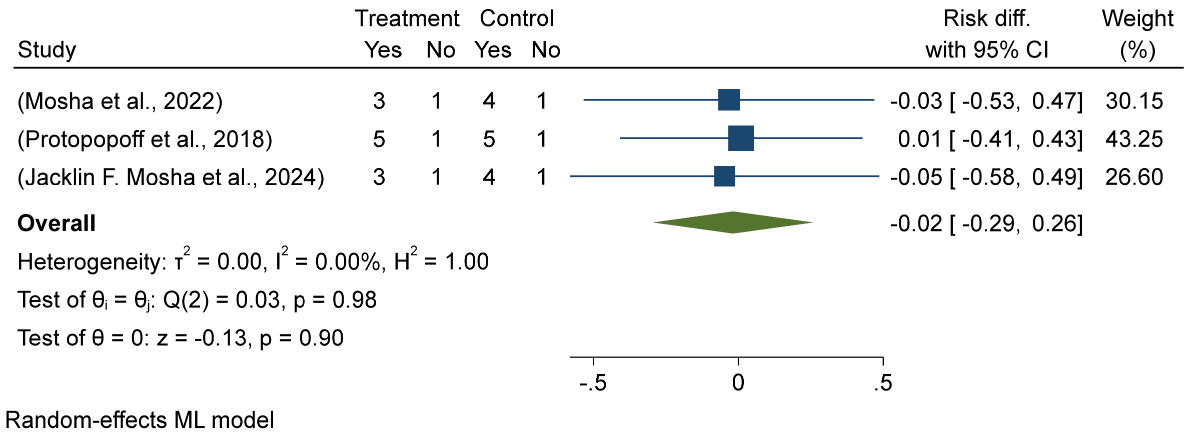

Figure S27: Forest plot shows the effectiveness and efficacy of Piperonyl butoxide long-lasting insecticidal nets (LLINs) in sporozoite rate reduction compared to pyrethroid-only LLINs in Africa in 2024.

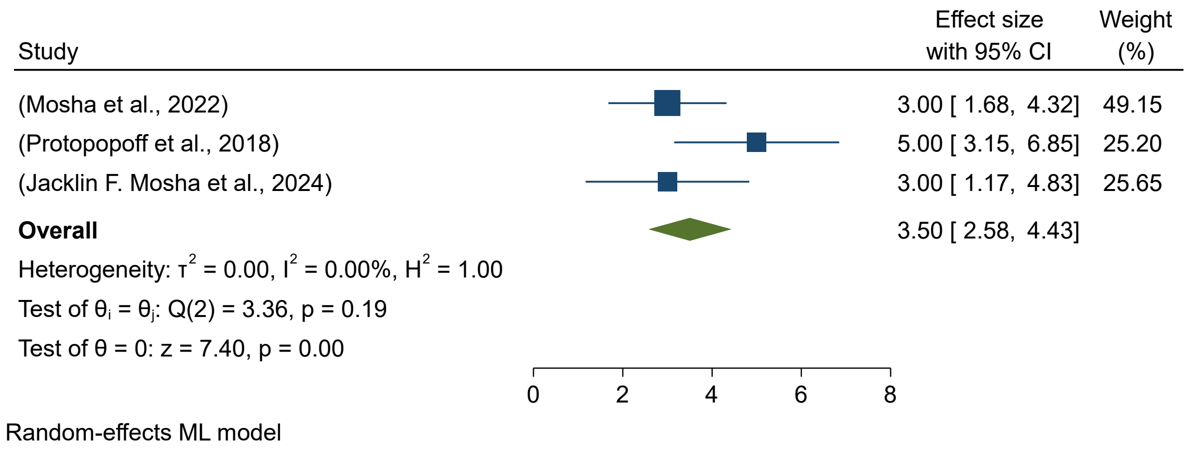

Figure S28: Forest plots showed baseline pooled sporozoite rate among Piperonyl butoxide LLINs intervention for malaria control in Africa in 2024.

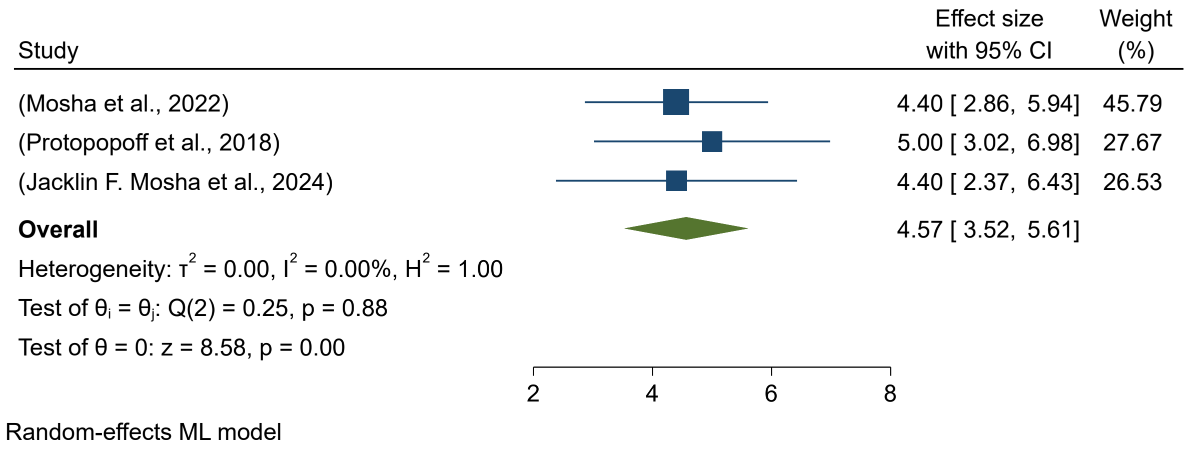

Figure S29: Forest plots showed baseline pooled sporozoite rate among Pyrethroid-only LLINs intervention for malaria control in Africa in 2024.

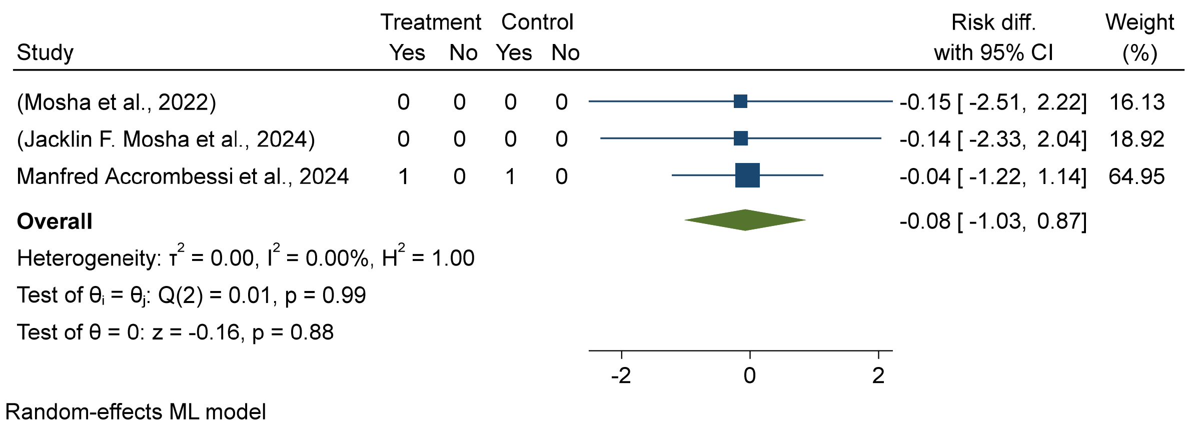

Figure S30: Forest plot shows the effectiveness and efficacy of Pyriproxyfen long-lasting insecticidal nets (LLINs) in mean entomological inoculation rate per household per night (MEIR) reduction compared to pyrethroid-only LLINs in Africa in 2024.

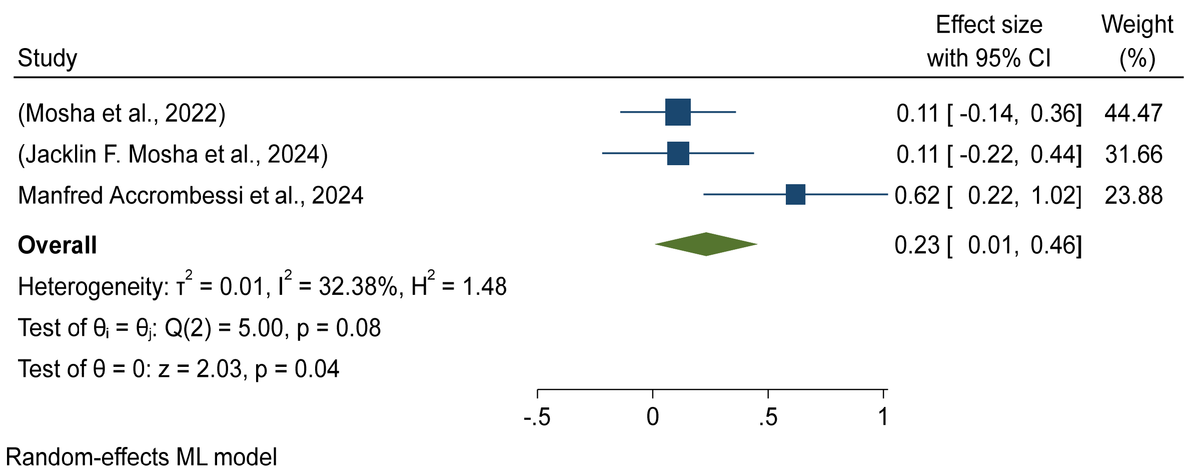

Figure S31: Forest plots showed baseline pooled mean entomological inoculation rate per household per night (MEIR) among Pyriproxyfen long-lasting insecticidal nets (LLINs) intervention for malaria control in Africa in 2024.

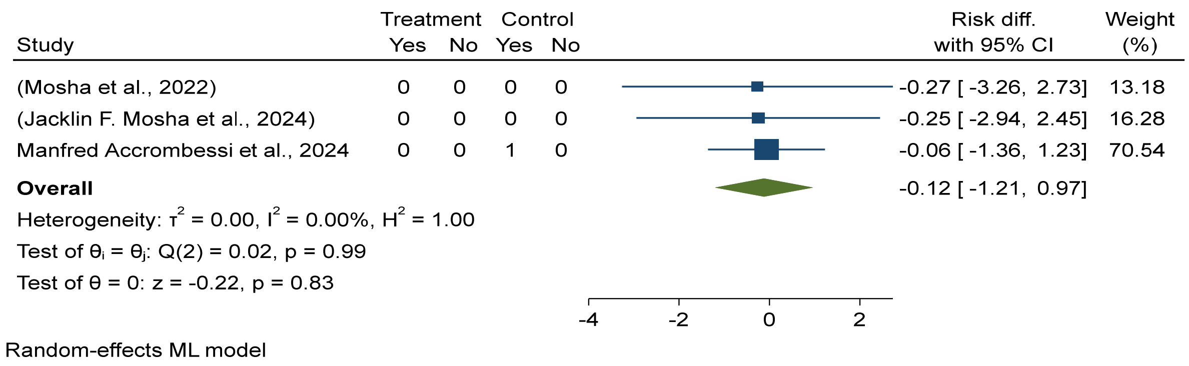

Figure S32: Forest plot shows the effectiveness and efficacy of chlorfenapyr long-lasting insecticidal nets (LLINs) in mean entomological inoculation rate per household per night (MEIR) reduction compared to pyrethroid-only LLINs in Africa in 2024.

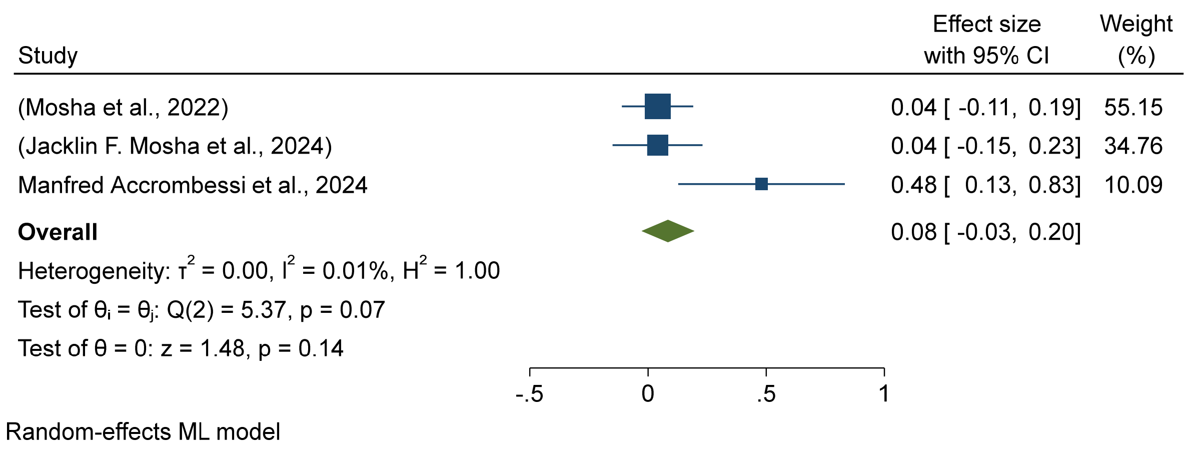

Figure S33: Forest plots showed baseline pooled mean entomological inoculation rate per household per night (MEIR) among Pyriproxyfen long-lasting insecticidal nets (LLINs) intervention for malaria control in Africa in 2024.

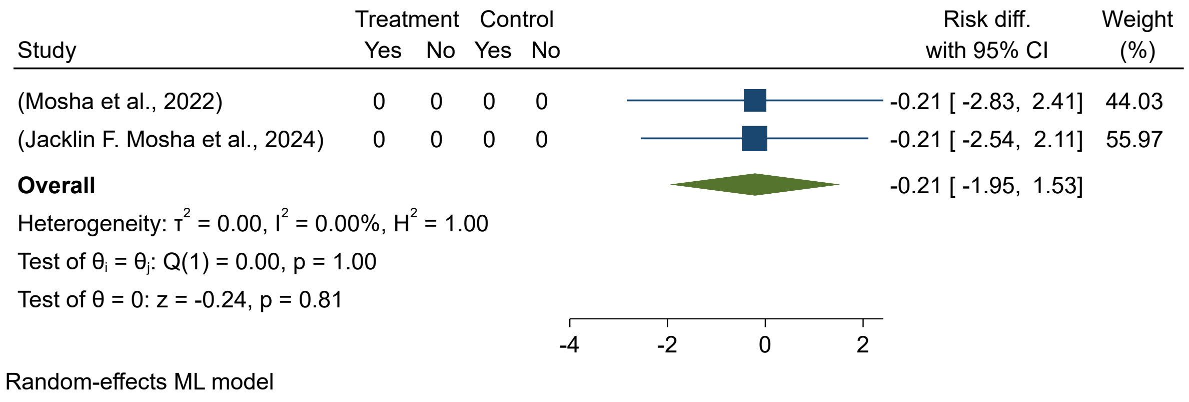

Figure S34: Forest plots showed baseline pooled mean entomological inoculation rate per household per night (MEIR) among Piperonyl butoxide long-lasting insecticidal nets (LLINs) intervention for malaria control in Africa in 2024

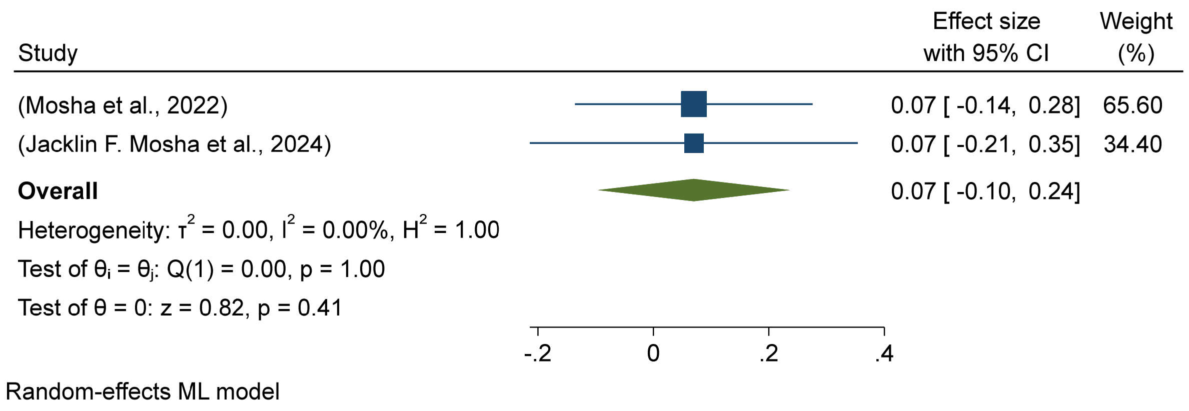

Figure S35: Forest plots showed baseline pooled mean entomological inoculation rate per household per night (MEIR) among Piperonyl butoxide long-lasting insecticidal nets (LLINs) intervention for malaria control in Africa in 2024.

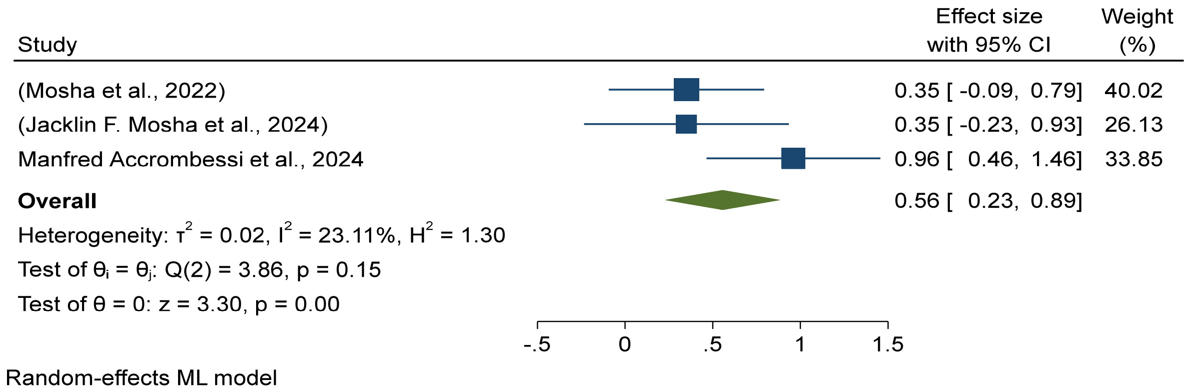

Figure S36: Forest plots showed baseline pooled mean entomological inoculation rate per household per night (MEIR) among pyrethroid-only long-lasting insecticidal nets (LLINs) intervention for malaria control in Africa in 2024.

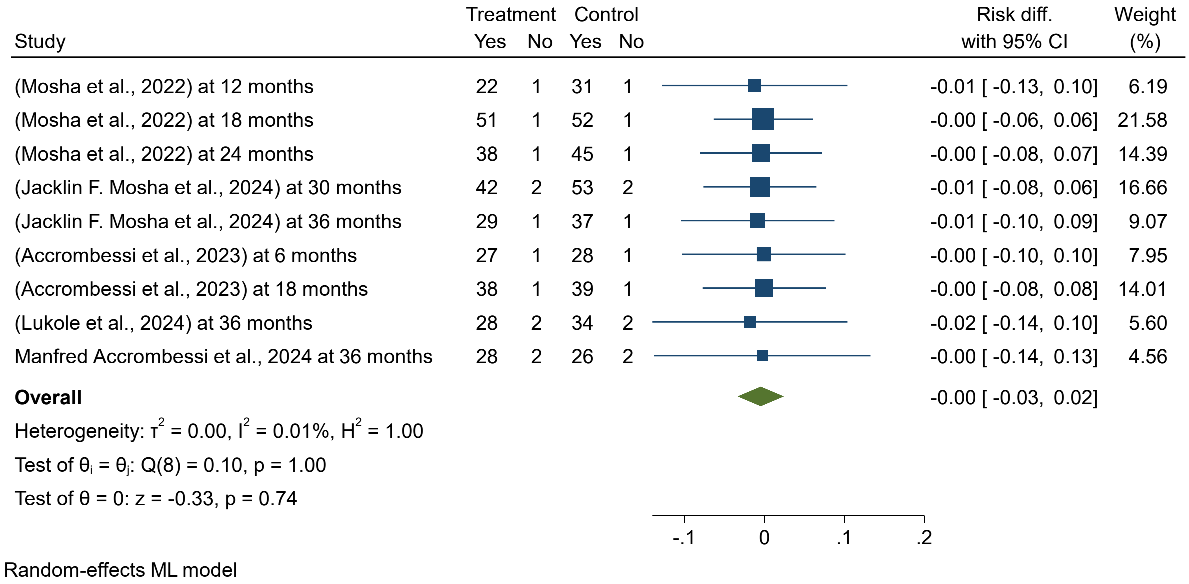

Figure S37: Forest plots shows Pooled post-intervention effectiveness and efficacy of Pyriproxyfen long-lasting insecticidal nets (LLINs) versus pyrethroid-only LLINs malaria infection reduction in Africa 2024.

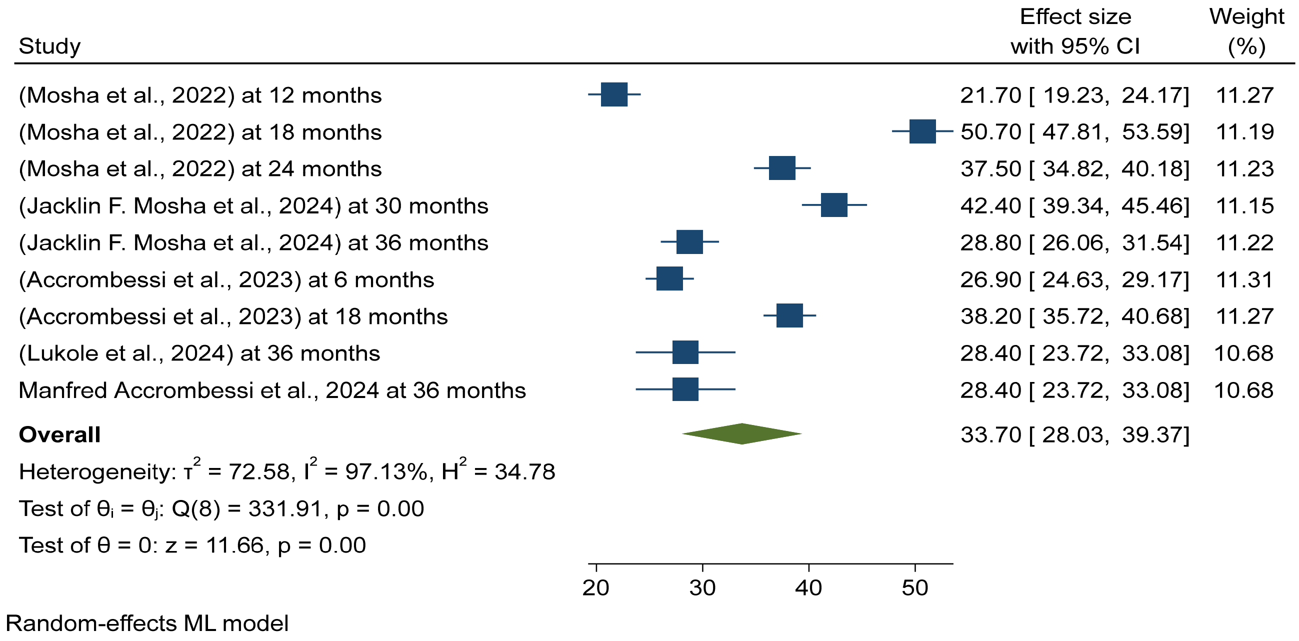

Figure S38: Forest plots shows Pooled post-intervention malaria infection prevalence in selected children using pyriproxyfen long-lasting insecticidal nets (LLINs) as malaria control in Africa 2024

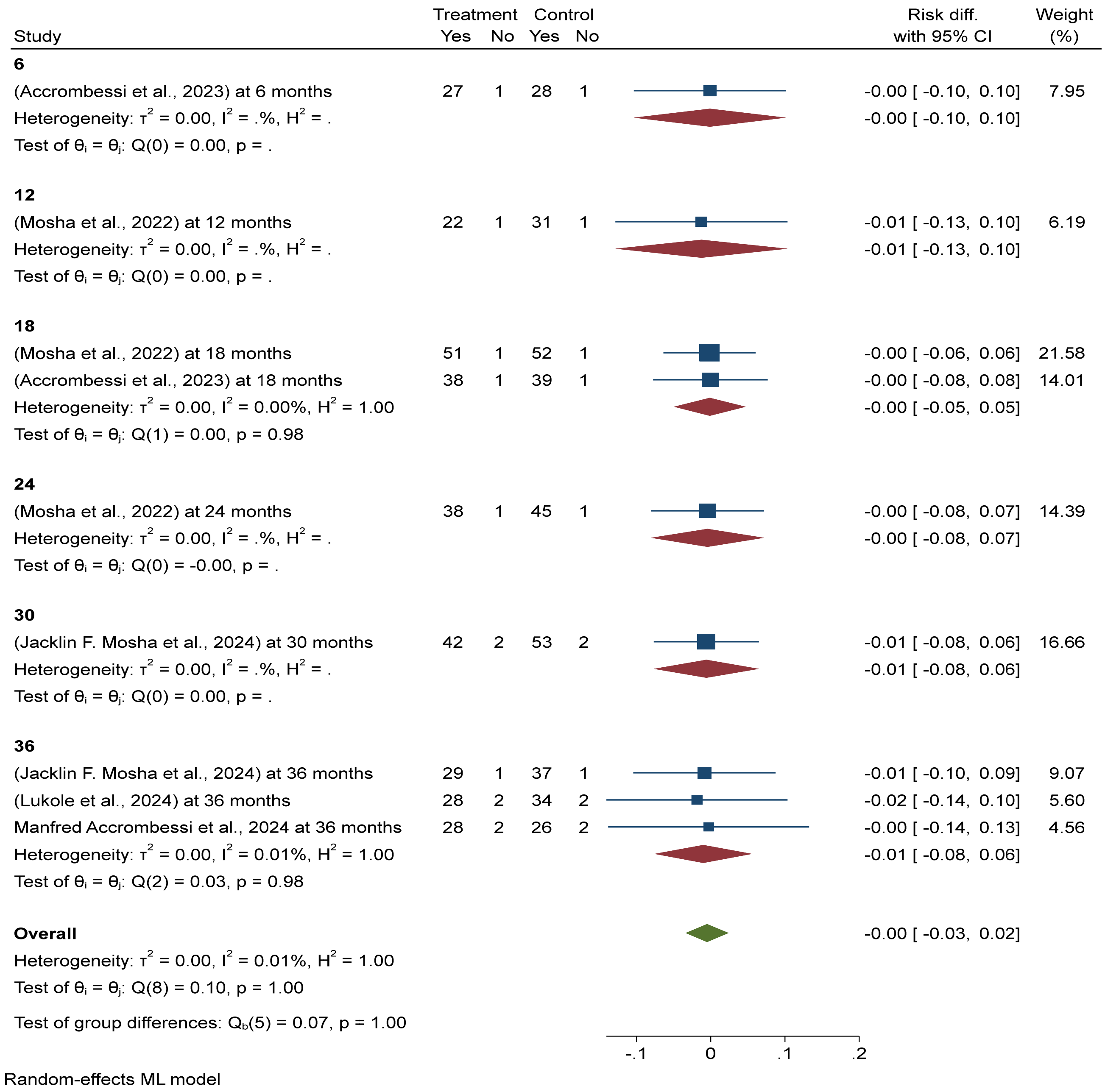

Figure S39: Forest plots shows subgroup analysis of post-intervention follow up effectiveness and efficacy of Pyriproxyfen long-lasting insecticidal nets (LLINs) versus pyrethroid-only LLINs malaria infection reduction in Africa 2024.

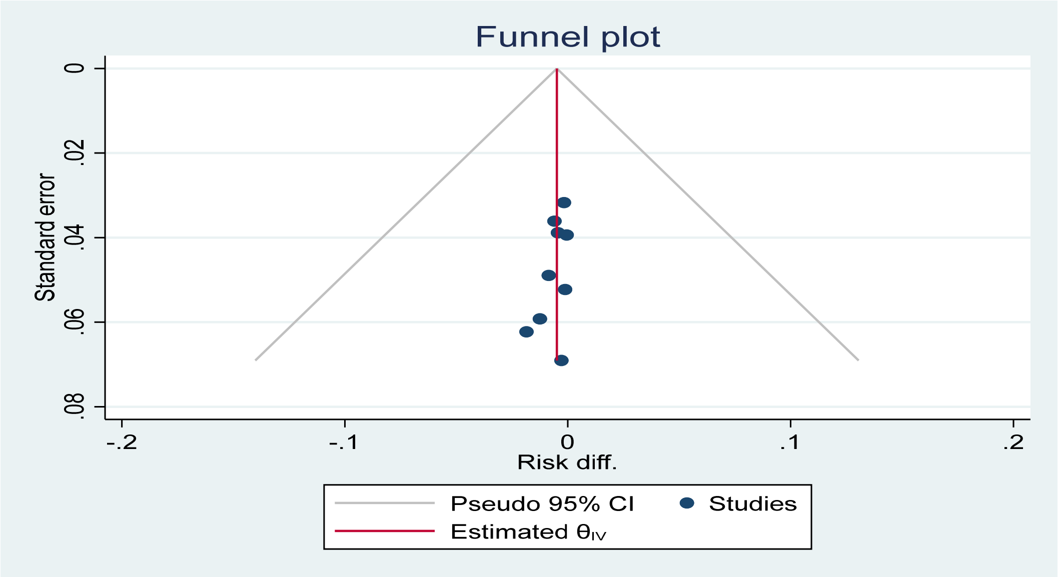

Figure S40**:** Funnel plot showing the distribution of included studies pooled malaria infection risk reduction among children using Pyriproxyfen long-lasting insecticidal nets (LLINs) versus pyrethroid-only LLINs for malaria control in Africa in 2024.

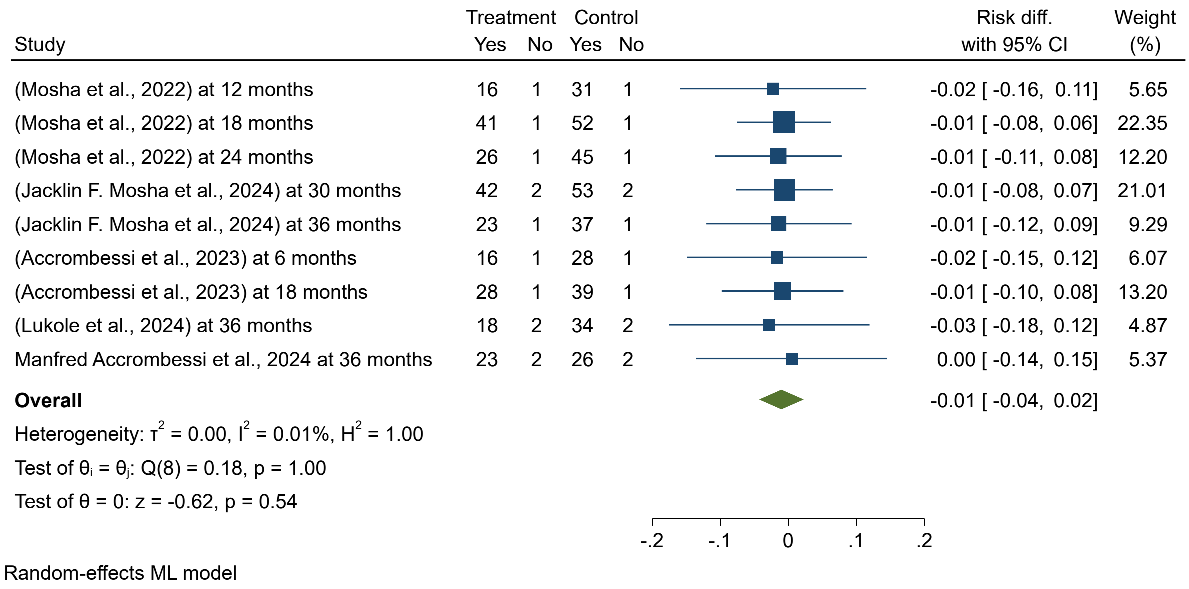

Figure S41: Forest plots shows Pooled post-intervention effectiveness and efficacy of chlorfenapyr long-lasting insecticidal nets (LLINs) versus pyrethroid-only LLINs malaria infection reduction in Africa 2024

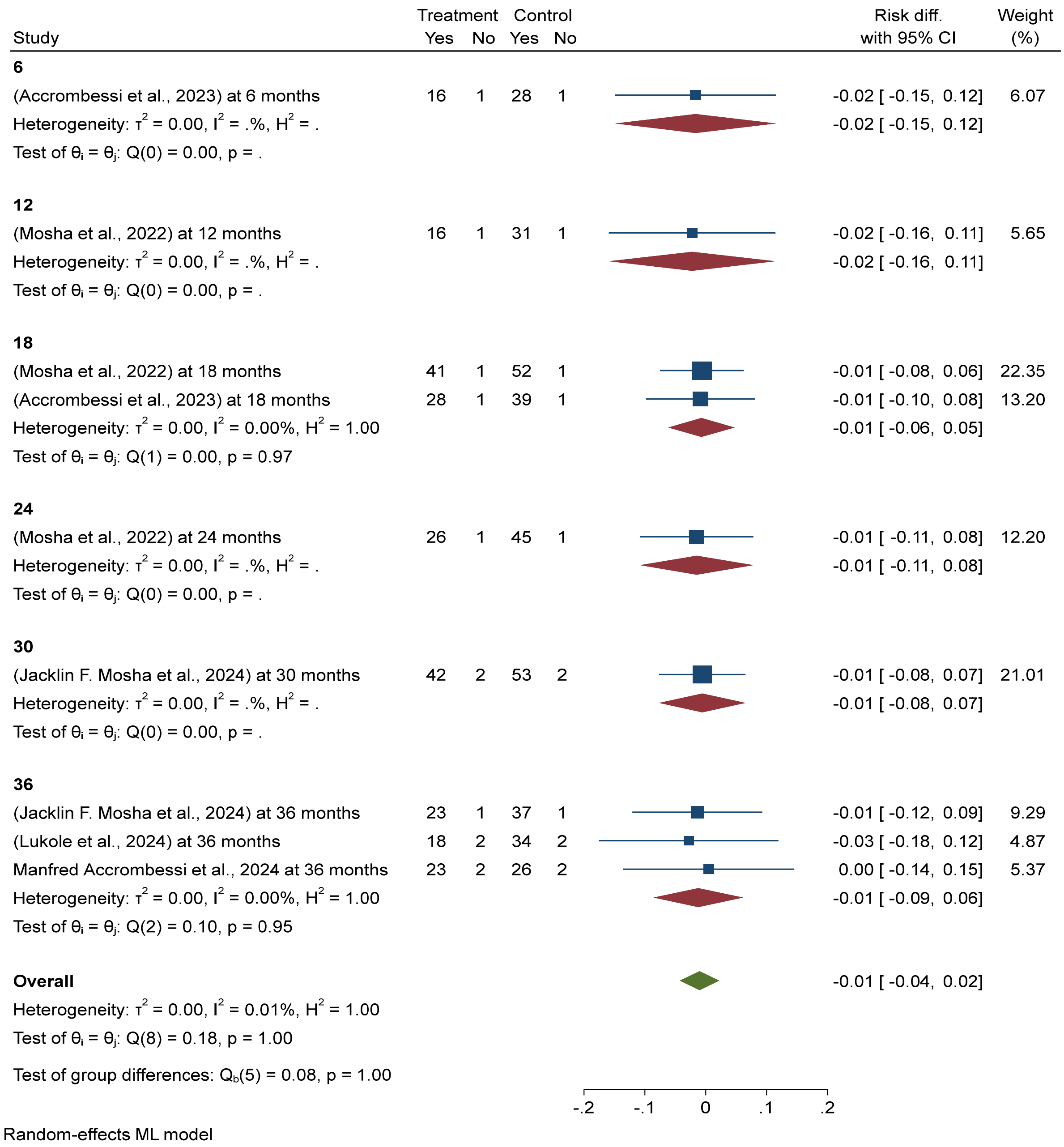

Figure S42: Forest plots shows subgroup analysis of post-intervention follow up effectiveness and efficacy of chlorfenapyr long-lasting insecticidal nets (LLINs) versus pyrethroid-only LLINs malaria infection reduction in Africa 2024.

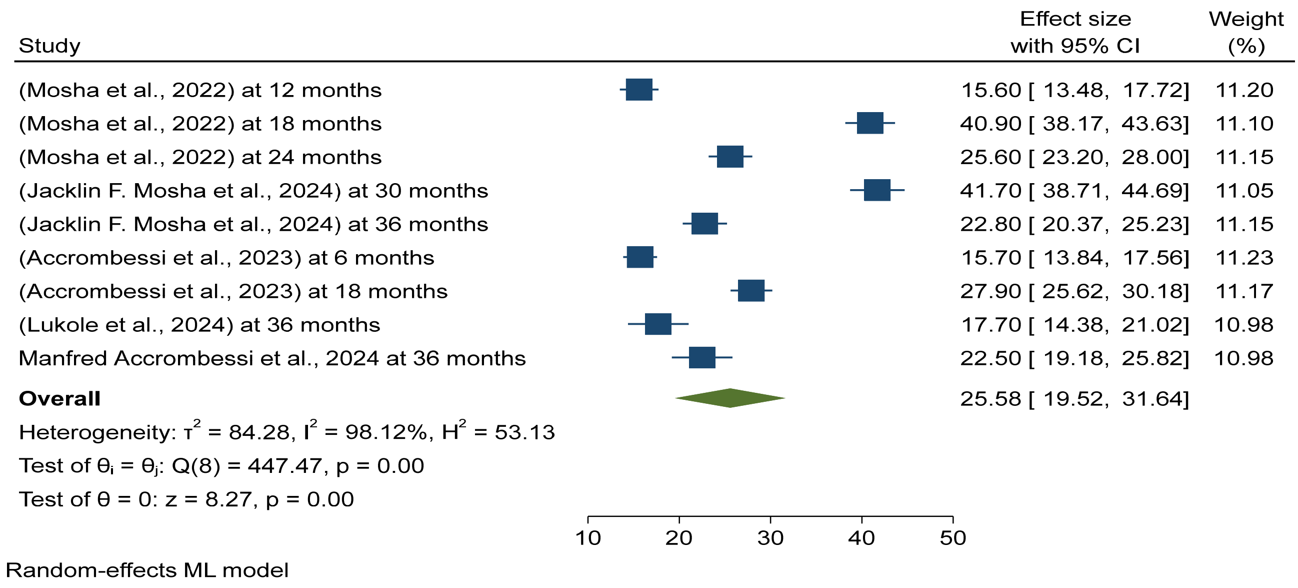

Figure S43: Forest plots shows Pooled post-intervention malaria infection prevalence in selected children using chlorfenapyr long-lasting insecticidal nets (LLINs) as malaria control in Africa 2024

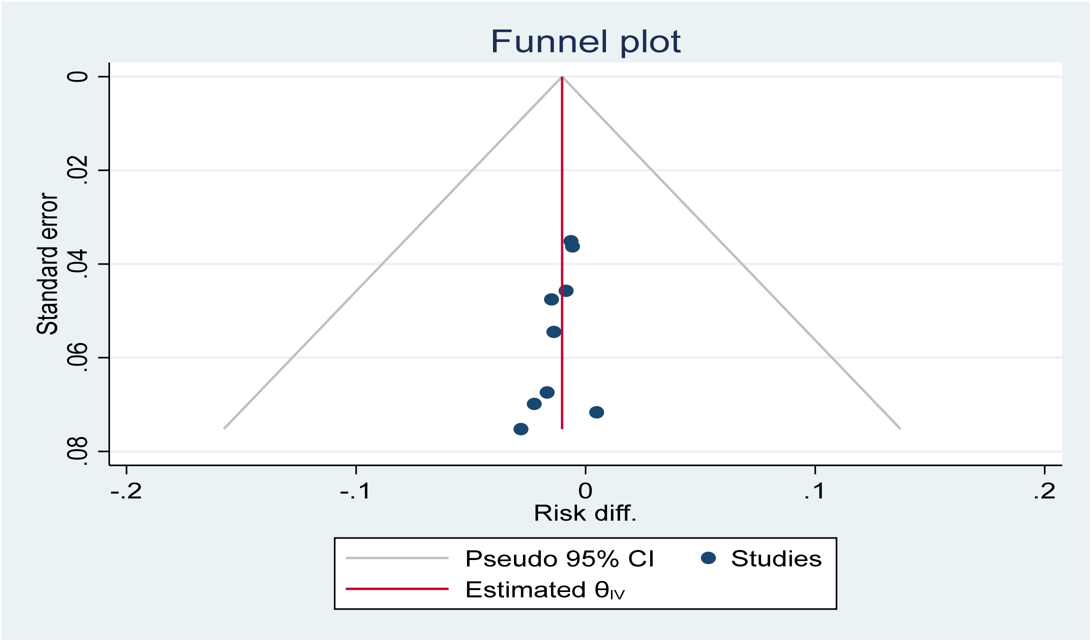

Figure S44: Funnel plot showing the distribution of included studies pooled malaria infection risk reduction among children using chlorfenapyr long-lasting insecticidal nets (LLINs) versus pyrethroid-only LLINs for malaria control in Africa in 2024.

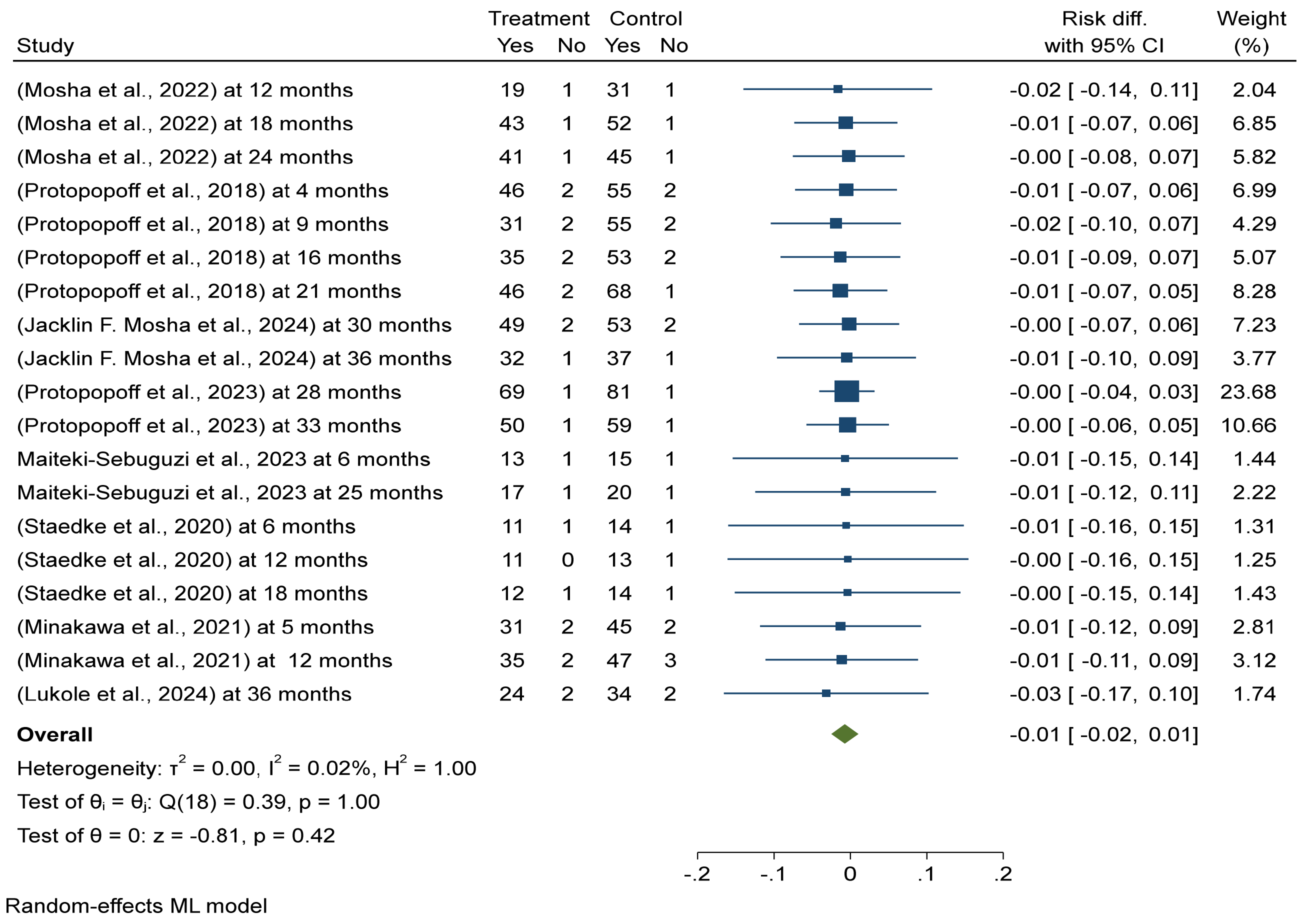

Figure S45: Forest plots shows Pooled post-intervention effectiveness and efficacy of Piperonyl butoxide long-lasting insecticidal nets (LLINs) versus pyrethroid-only LLINs malaria infection reduction in Africa 2024

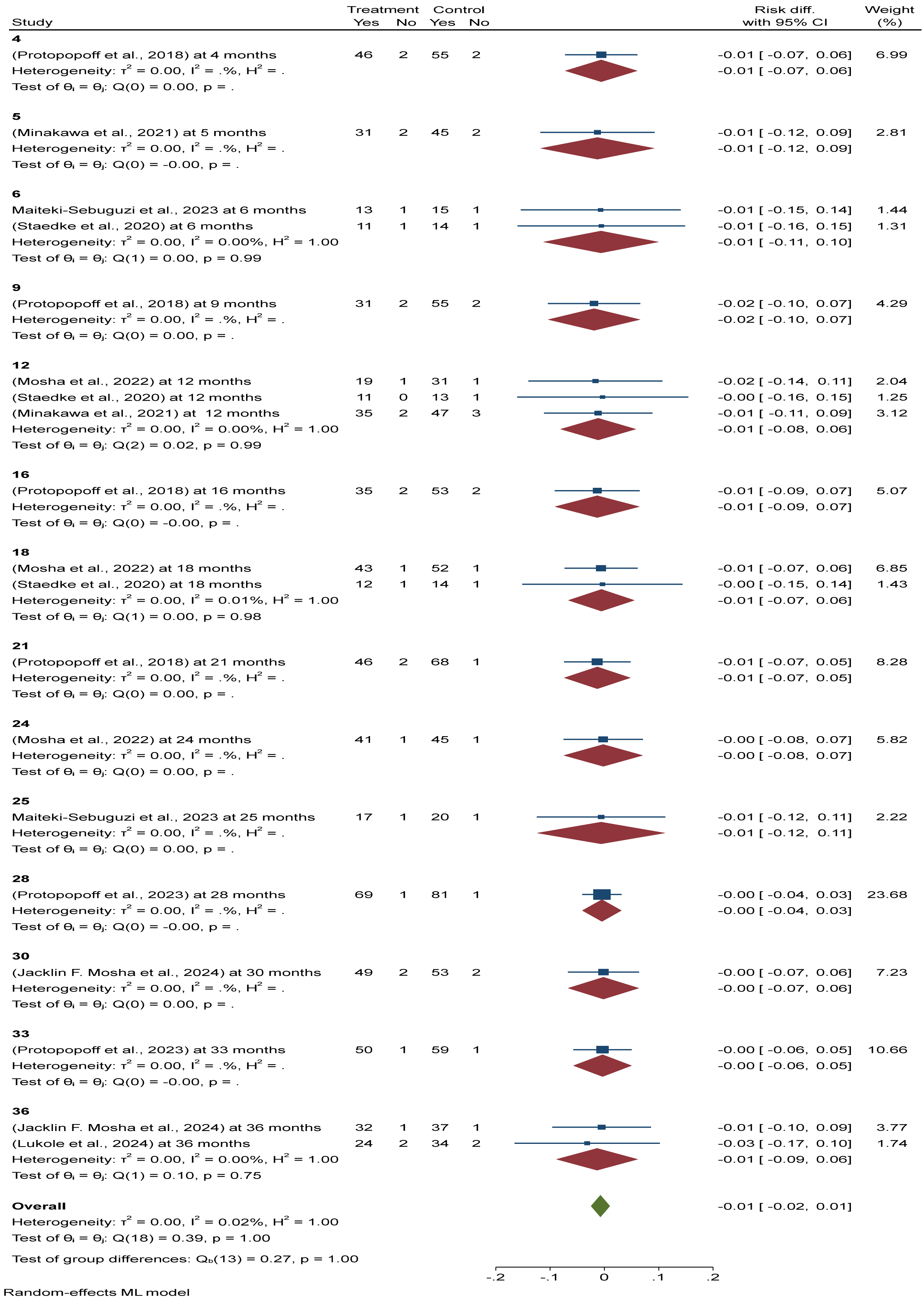

Figure S46: Forest plots shows subgroup analysis of post-intervention follow up effectiveness and efficacy of piperonyl butoxide long-lasting insecticidal nets (LLINs) versus pyrethroid-only LLINs malaria infection reduction in Africa 2024.

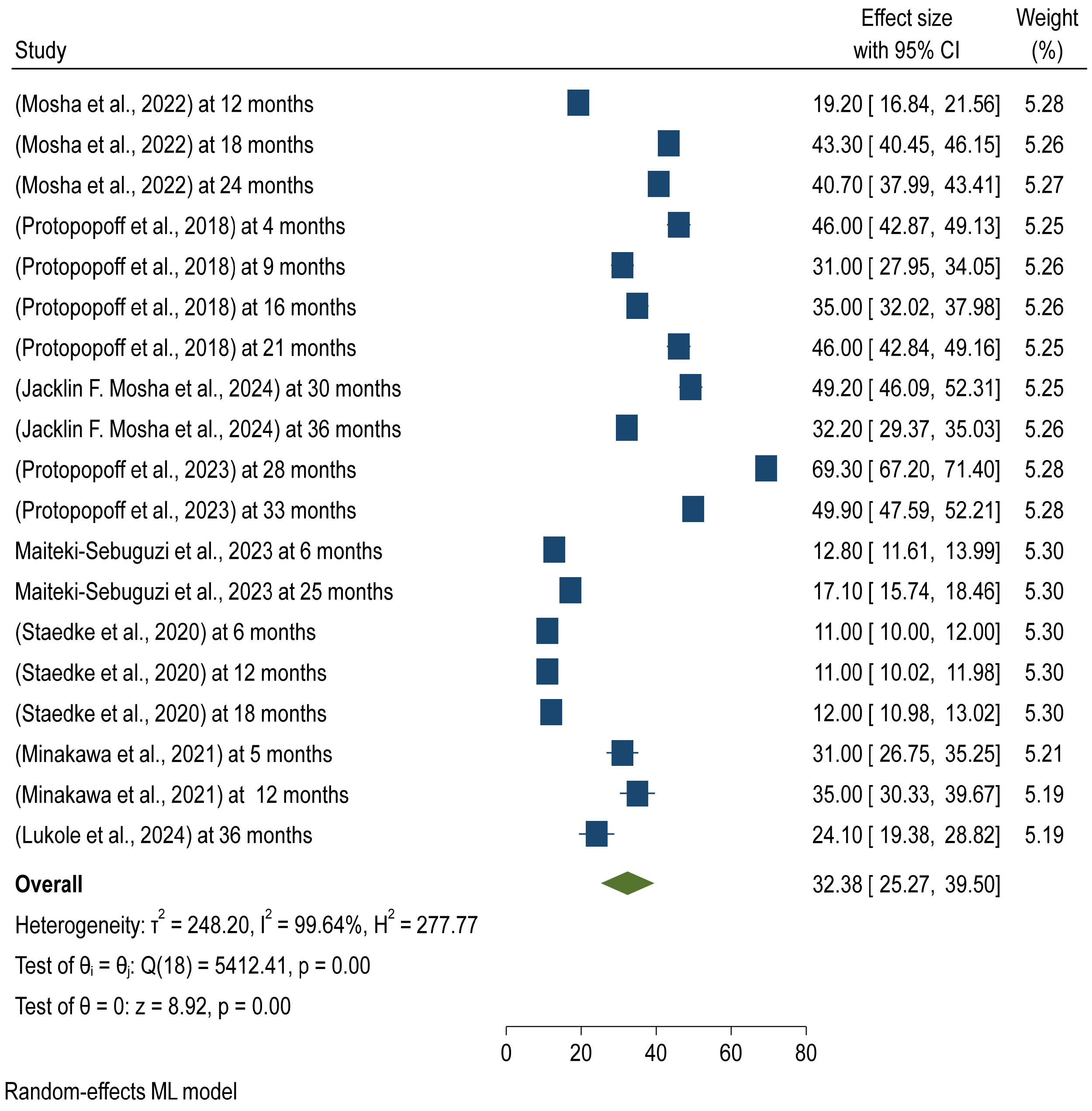

Figure S47: Forest plots shows Pooled post-intervention malaria infection prevalence in selected children using piperonyl butoxide long-lasting insecticidal nets (LLINs) in Africa 2024

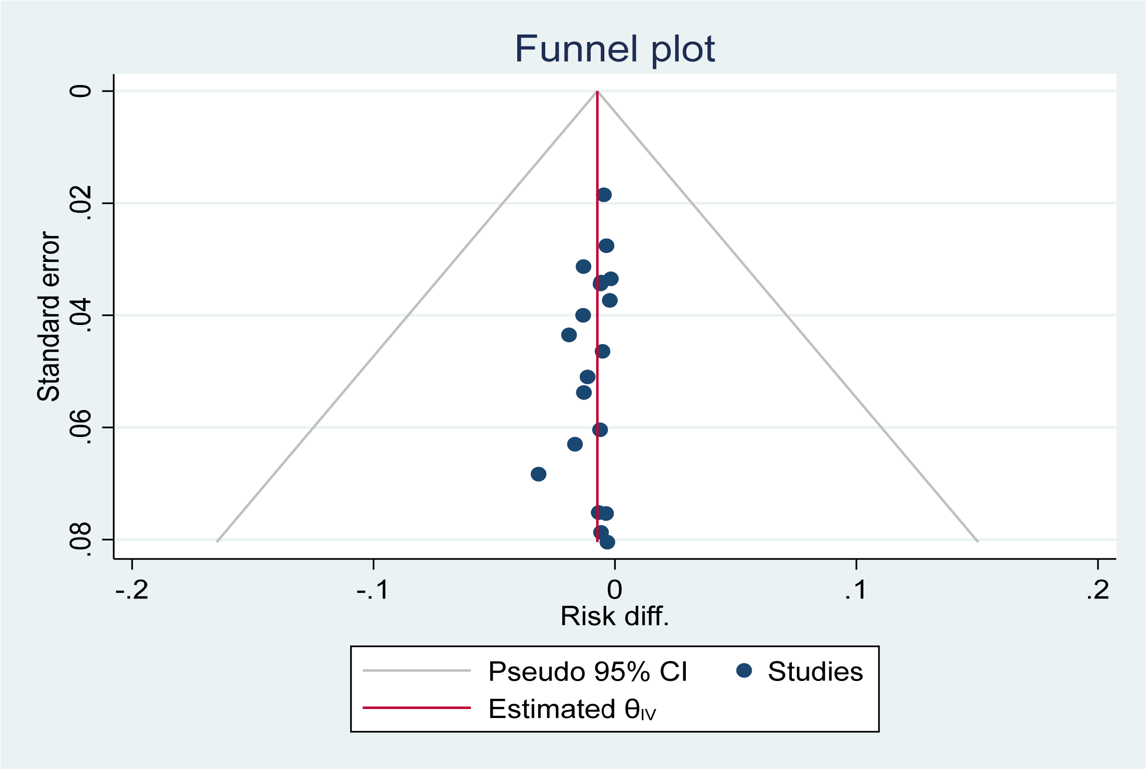

Figure S48: Funnel plot showing the distribution of included studies pooled malaria infection risk reduction among children using piperonyl butoxide long-lasting insecticidal nets (LLINs) versus pyrethroid-only LLINs for malaria control in Africa in 2024.

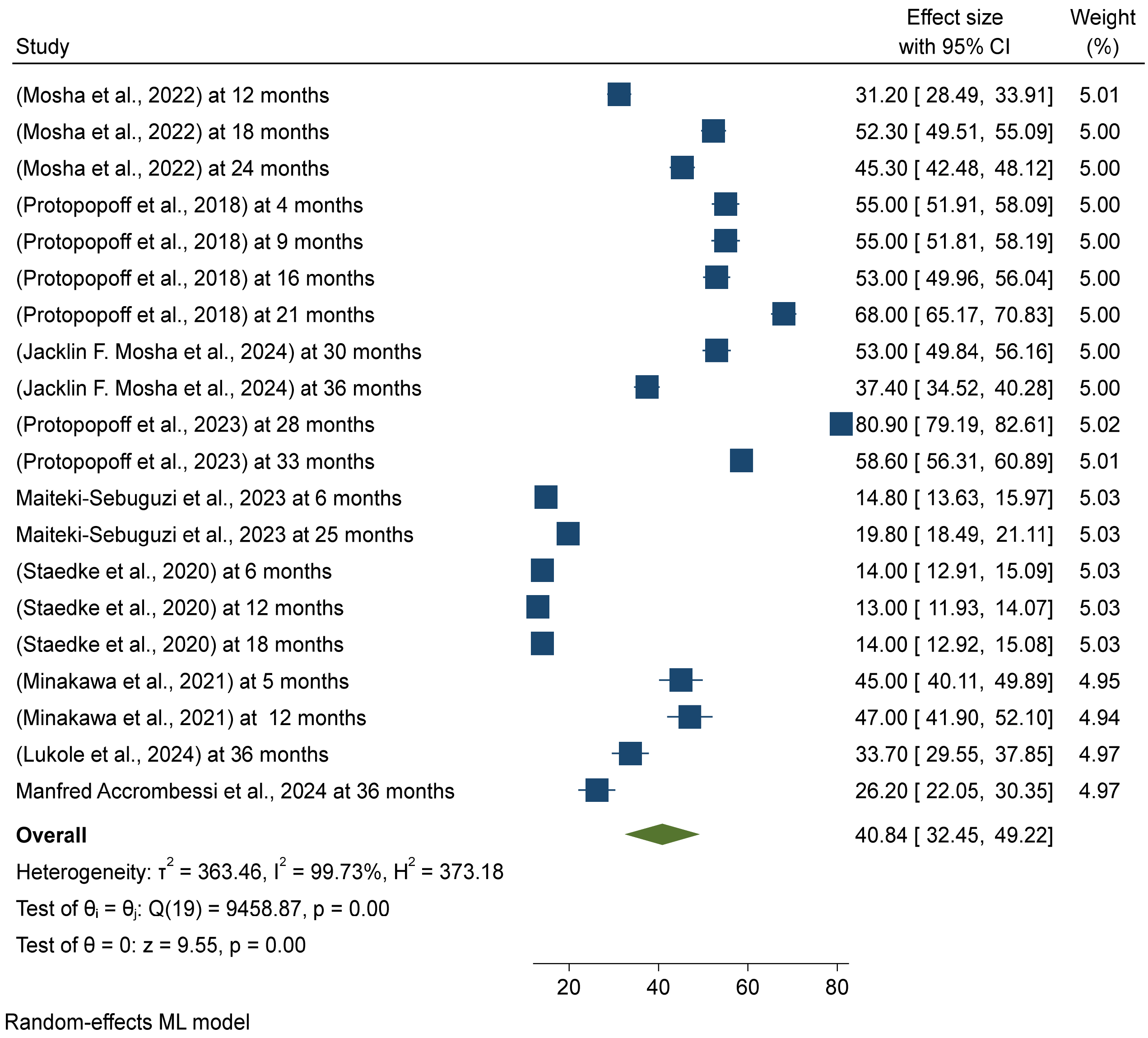

Figure S49: Forest plots shows pooled post-intervention malaria infection prevalence in selected children using pyrethroid-only long-lasting insecticidal nets (LLINs) or control group in Africa 2024

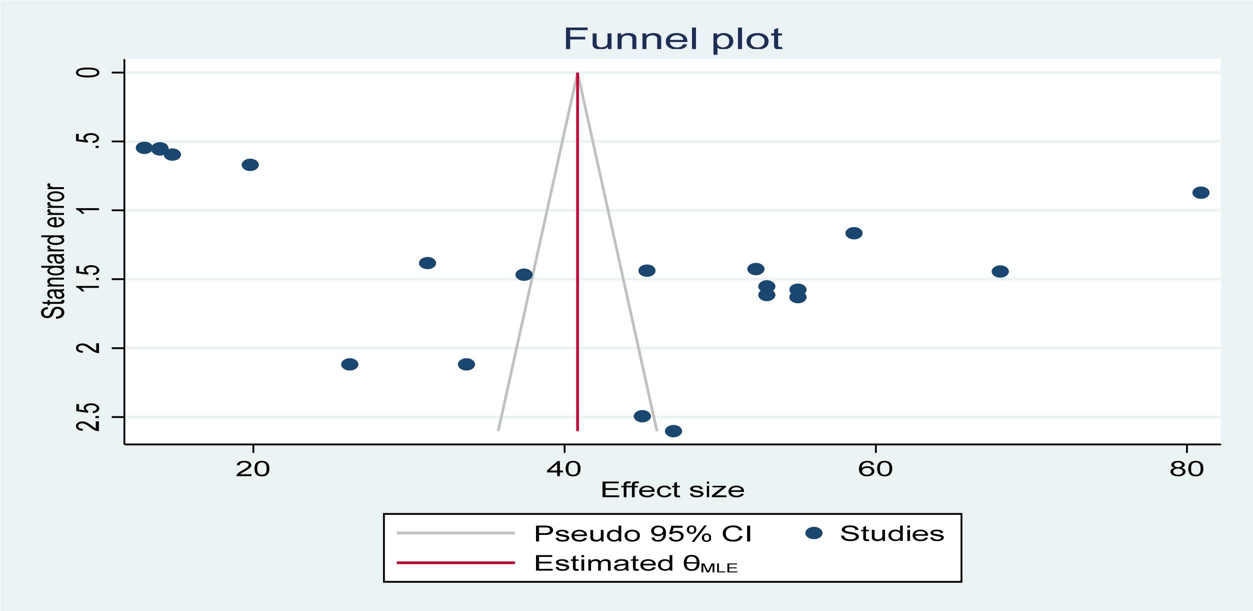

Figure S50: Funnel plot showing the distribution of included studies pooled malaria infection risk reduction among children using pyrethroid-only long-lasting insecticidal nets (LLINs) for malaria control in Africa in 2024

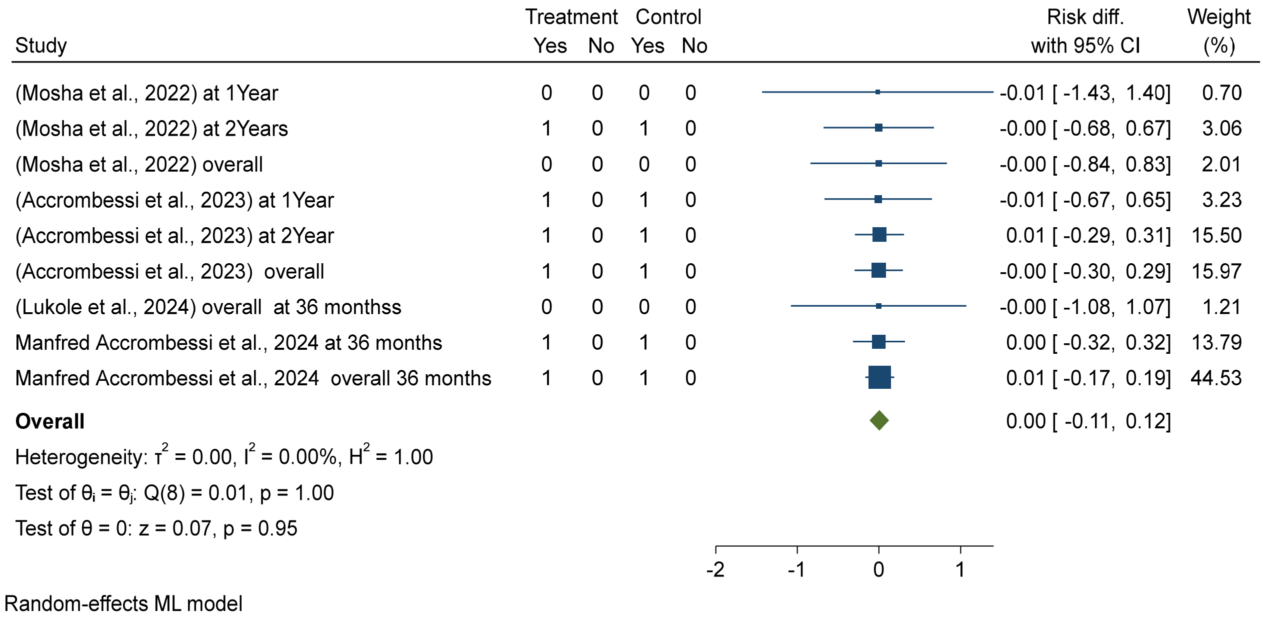

Figure S51: Forest plots shows Pooled post-intervention effectiveness and efficacy of Pyriproxyfen long-lasting insecticidal nets (LLINs) versus pyrethroid-only LLINs Malaria case incidence reduction in children (aged 6 months to 10 years in Africa

Publication bias was checked using funnel plots looking at symmetrical distribution, and it was objectively verified using Egger's regre Sion test, which revealed that there was no publication bias ($p < 0.066$) (Figure S52).

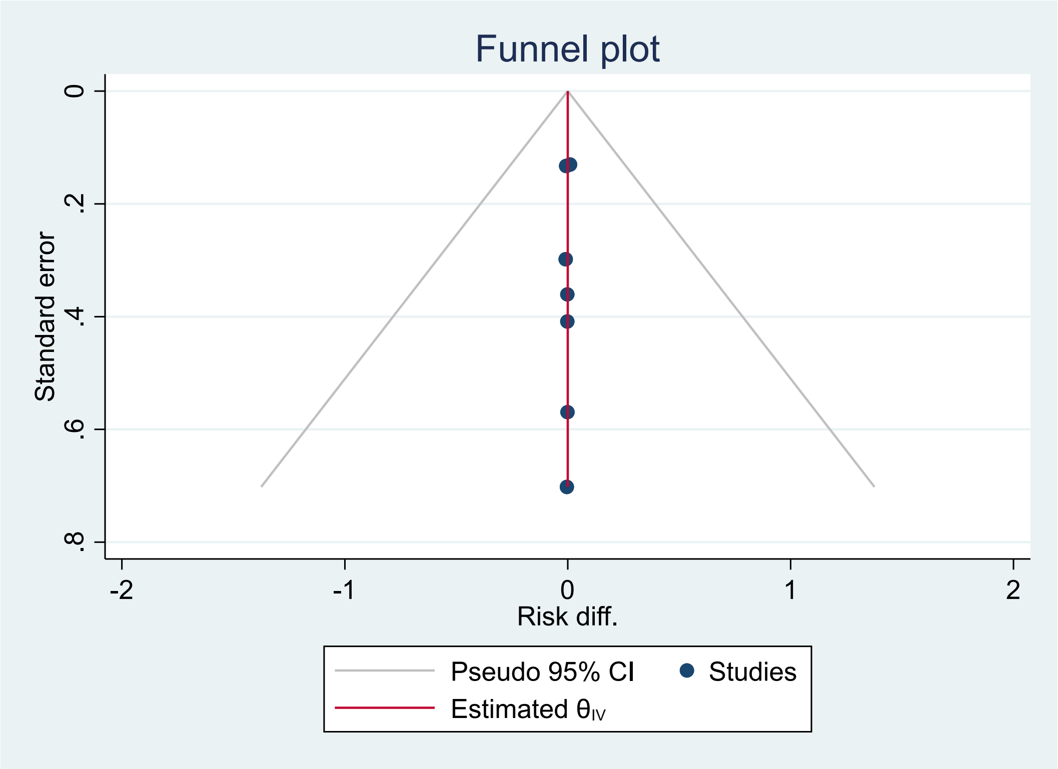

Figure S52: Funnel plot showing the distribution of included studies pooled effectiveness and efficacy of Pyriproxyfen long-lasting insecticidal nets (LLINs) versus pyrethroid-only LLINs Malaria case incidence reduction in children (aged 6 months to 10 years in Africa in 2024

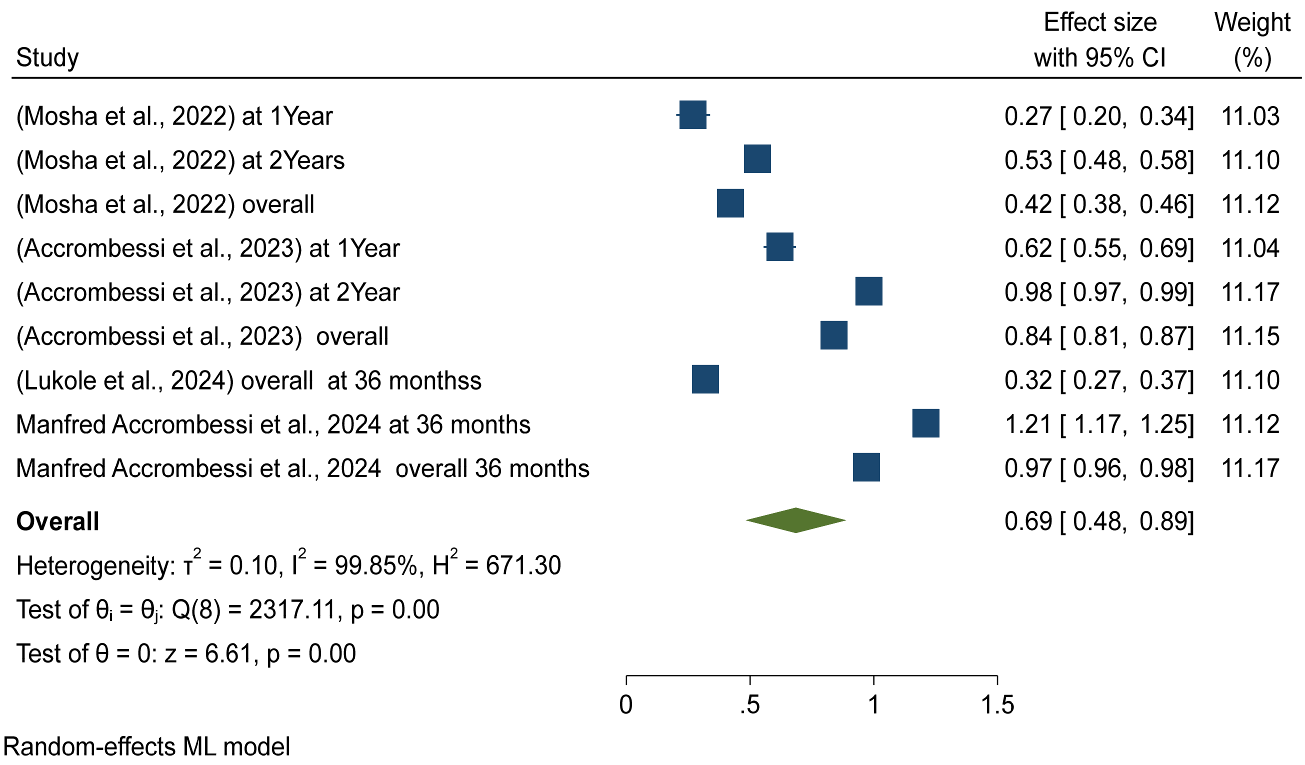

Figure S53: Forest plots shows pooled post-intervention Malaria case incidence reduction in children (aged 6 months to 10 years using Pyriproxyfen long-lasting insecticidal nets (LLINs) versus pyrethroid-only LLINs in Africa 2024

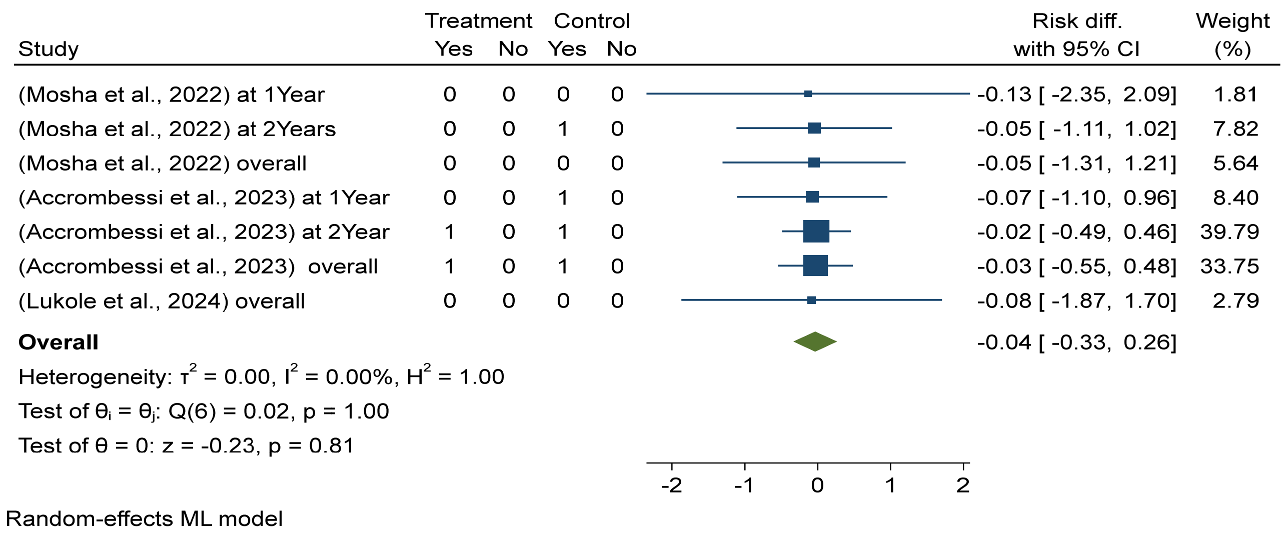

Figure S54: Forest plots shows pooled post-intervention effectiveness and efficacy of chlorfenapyr long-lasting insecticidal nets (LLINs) versus pyrethroid-only LLINs malaria case incidence reduction in children aged 6 months to 10 years in Africa 2024

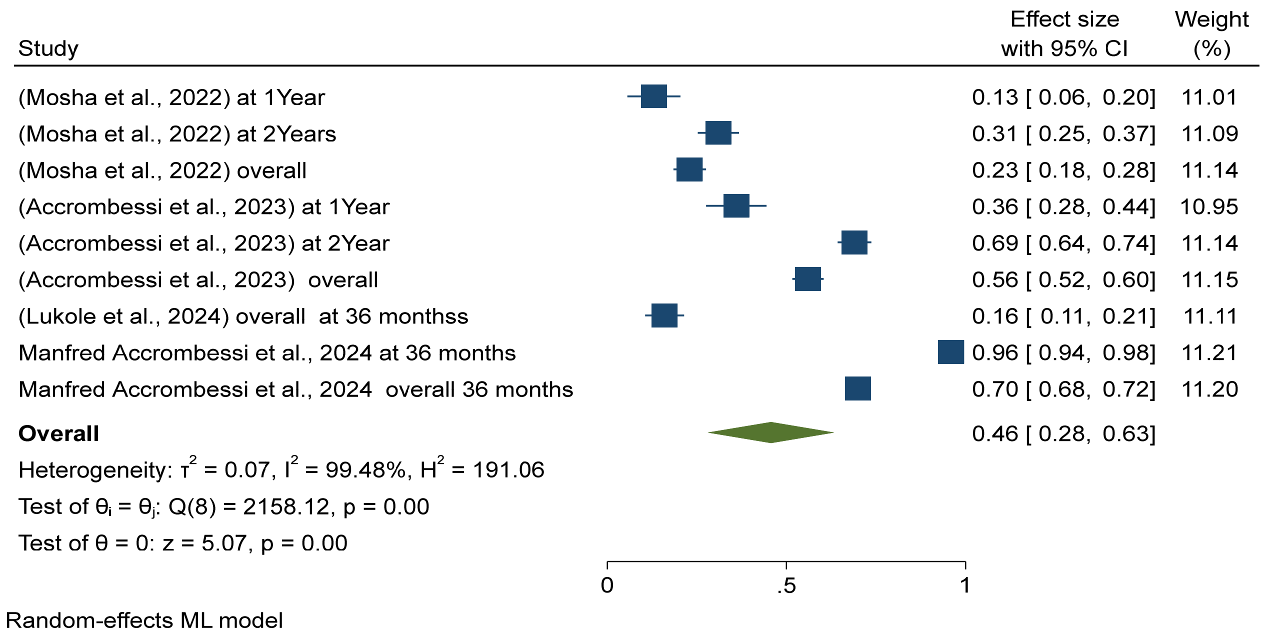

Figure S55: Forest plots shows pooled post-intervention Malaria case incidence reduction in children (aged 6 months to 10 years using chlorfenapyr long-lasting insecticidal nets (LLINs) for malaria control in Africa 2024

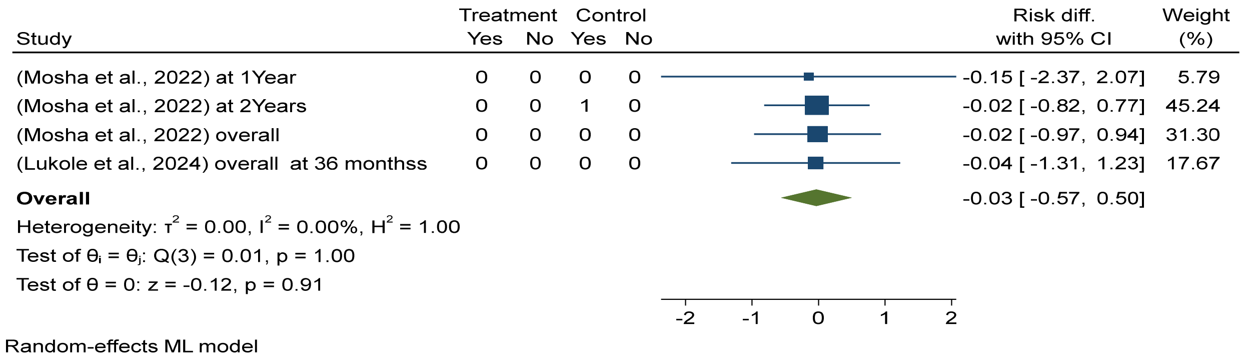

Figure S56: Forest plots shows pooled post-intervention effectiveness and efficacy of Piperonyl butoxide long-lasting insecticidal nets (LLINs) versus pyrethroid-only LLINs malaria case incidence reduction in children aged 6 months to 10 years in Africa 2024

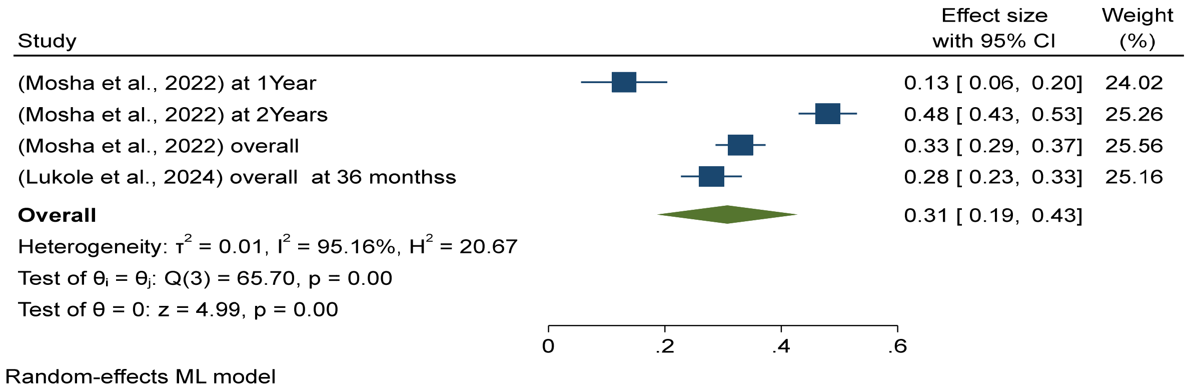

Figure S57: Forest plots shows pooled post-intervention Malaria case incidence reduction in children (aged 6 months to 10 years using piperonyl butoxide long-lasting insecticidal nets (LLINs) for malaria control in Africa 2024

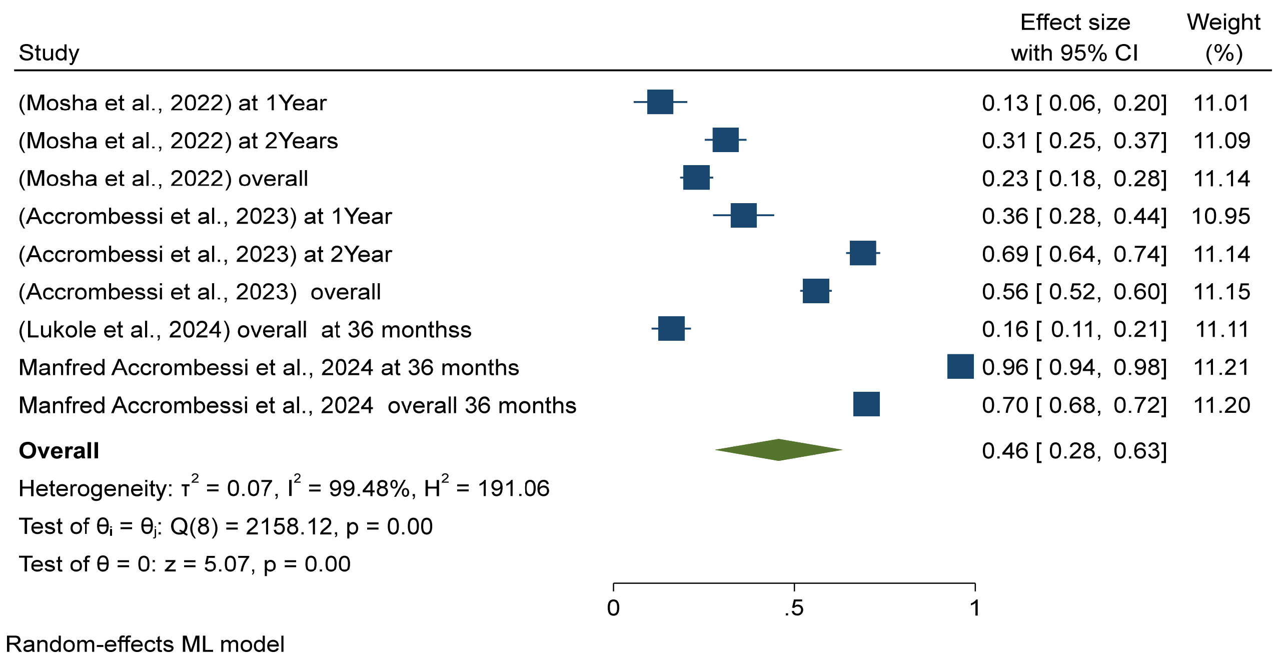

Figure S58: Forest plots shows pooled post-intervention Malaria case incidence reduction in children (aged 6 months to 10 years using pyrethroid-only long-lasting insecticidal nets (LLINs) for malaria control in Africa 2024

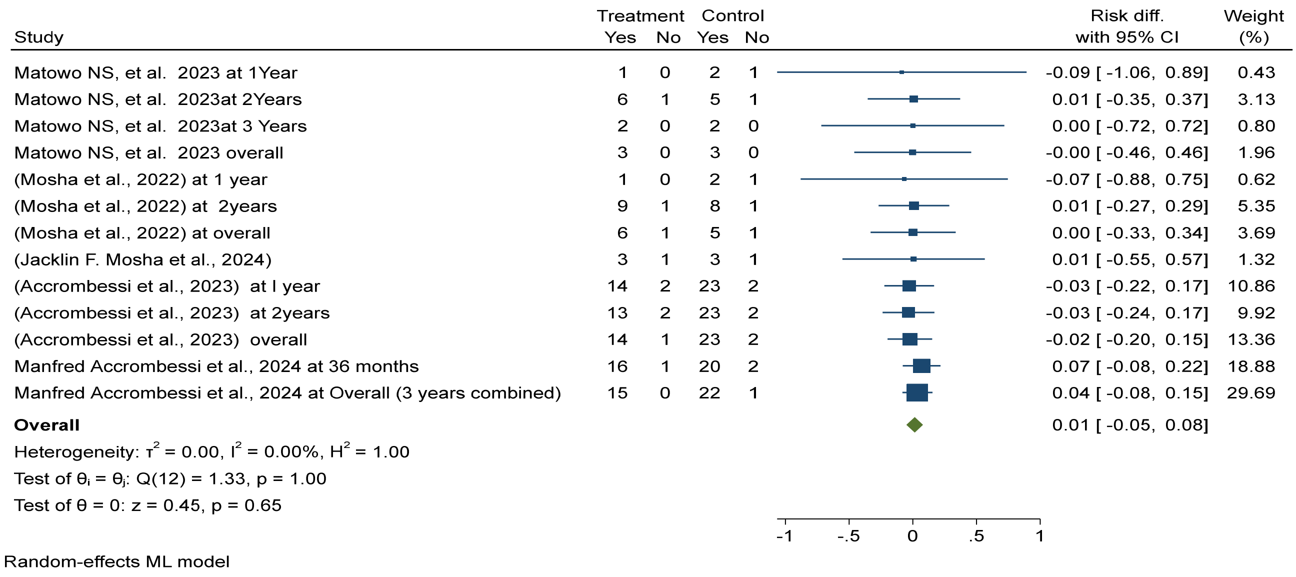

Figure S59: Forest plot shows the effectiveness and efficacy of pyriproxyfen long-lasting insecticidal nets (LLINs) in pooled mean indoor vector density reduction compared to pyrethroid-only LLINs in Africa in 2024.

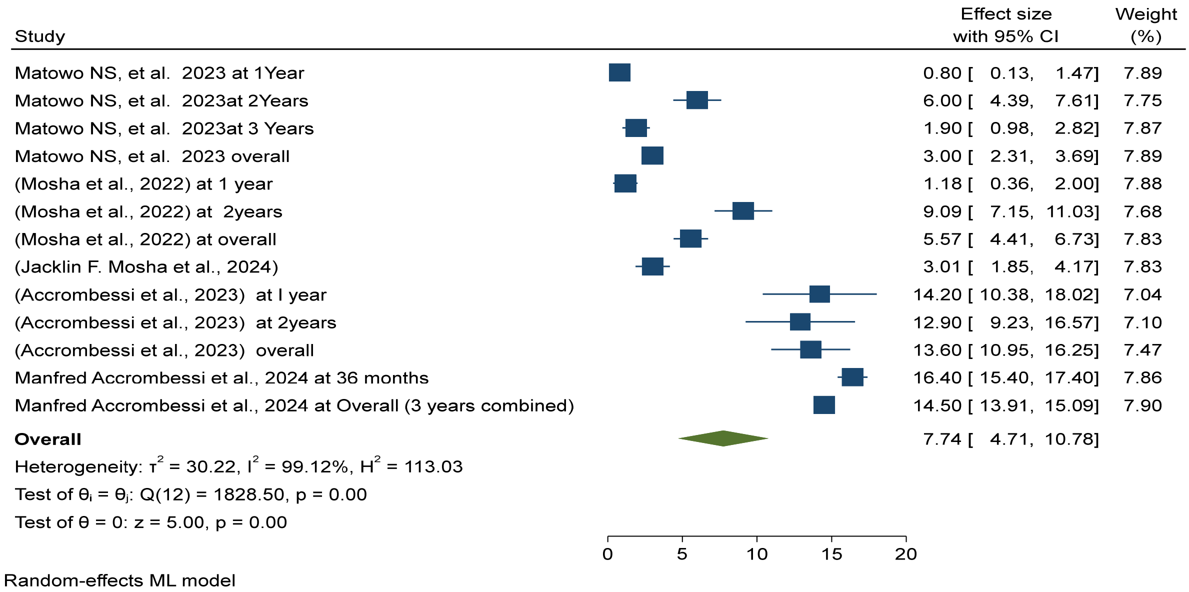

Figure S60: Forest plot shows post-intervention pooled mean indoor vector density per household per nights among pyriproxyfen long-lasting insecticidal nets intervention in Africa 2024

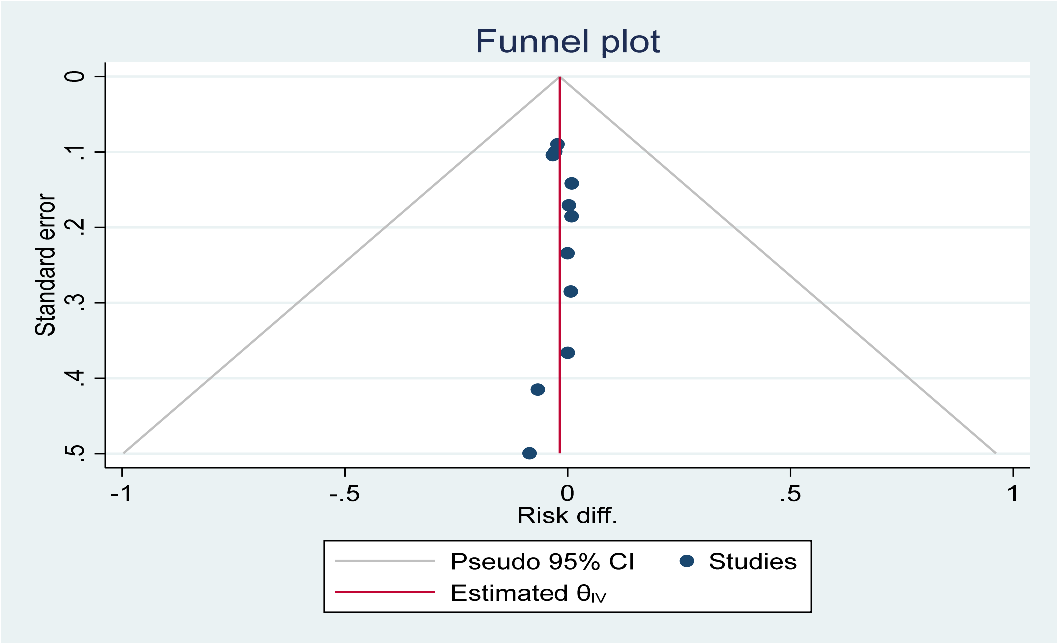

Figure S61: Funnel plot showing the distribution of included studies post-intervention pooled mean indoor vector density per household per nights among pyriproxyfen long-lasting insecticidal nets intervention in Africa 2024

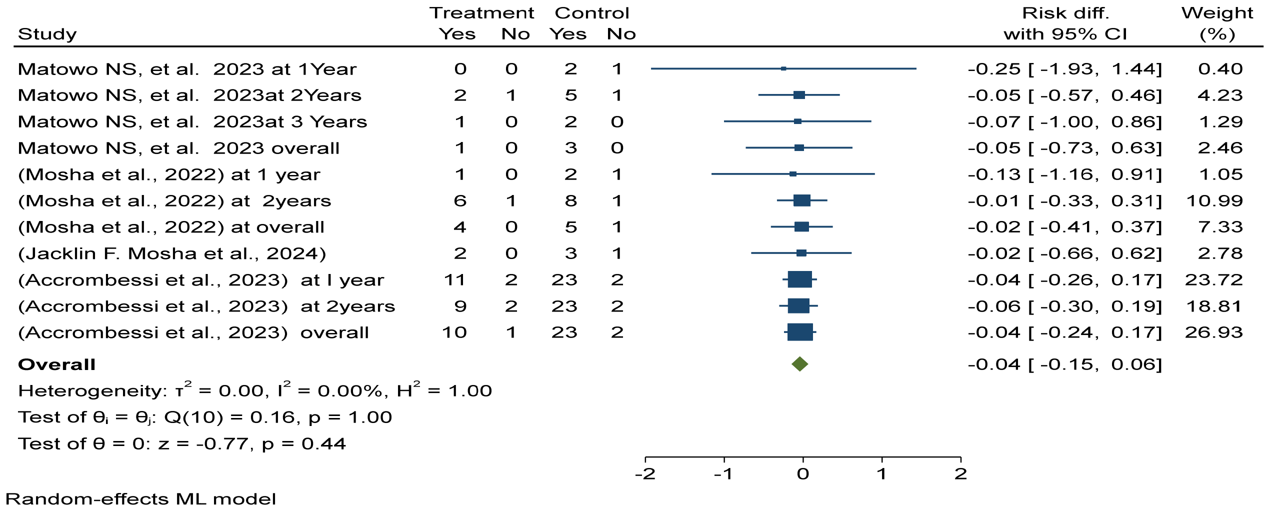

Figure S62: Forest plot shows the effectiveness and efficacy of chlorfenapyr long-lasting insecticidal nets (LLINs) in pooled mean indoor vector density reduction compared to pyrethroid-only LLINs in Africa in 2024.

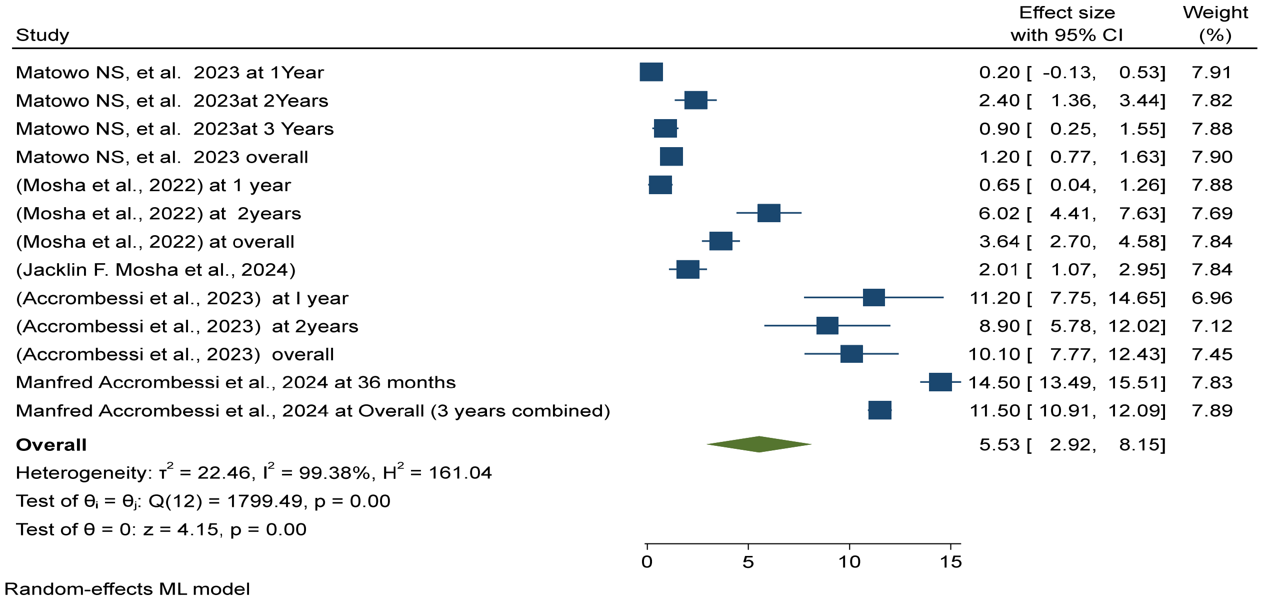

Figure S63: Forest plot shows post-intervention pooled mean indoor vector density per household per nights among chlorfenapy long-lasting insecticidal nets intervention in Africa 2024

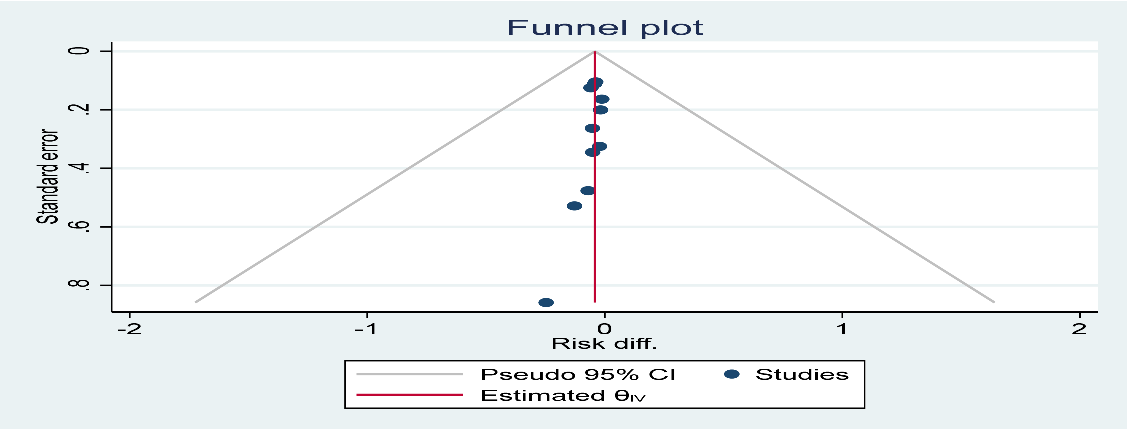

Figure S64: Funnel plot showing the distribution of included studies post-intervention pooled mean indoor vector density per household per nights among chlorfenapy long-lasting insecticidal nets intervention in Africa 2024

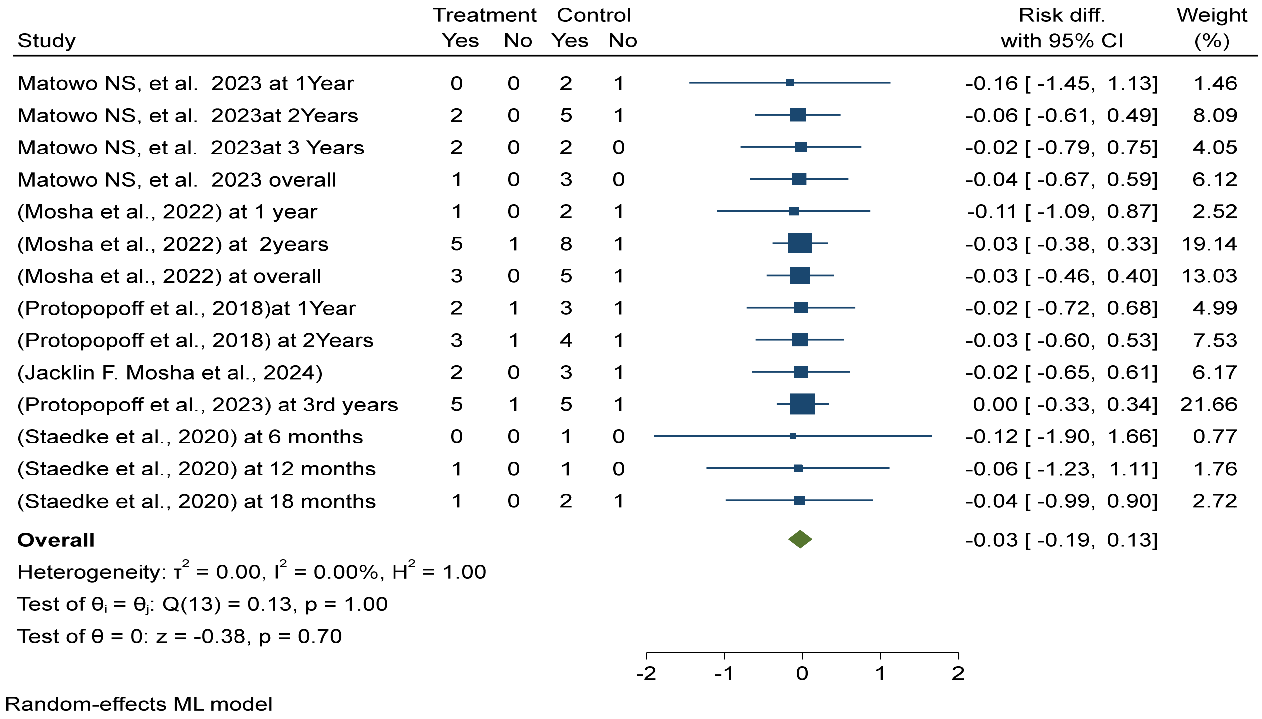

Figure S65: Forest plot shows the effectiveness and efficacy of Piperonyl butoxide long-lasting insecticidal nets (LLINs) in pooled mean indoor vector density reduction compared to pyrethroid-only LLINs in Africa in 2024.

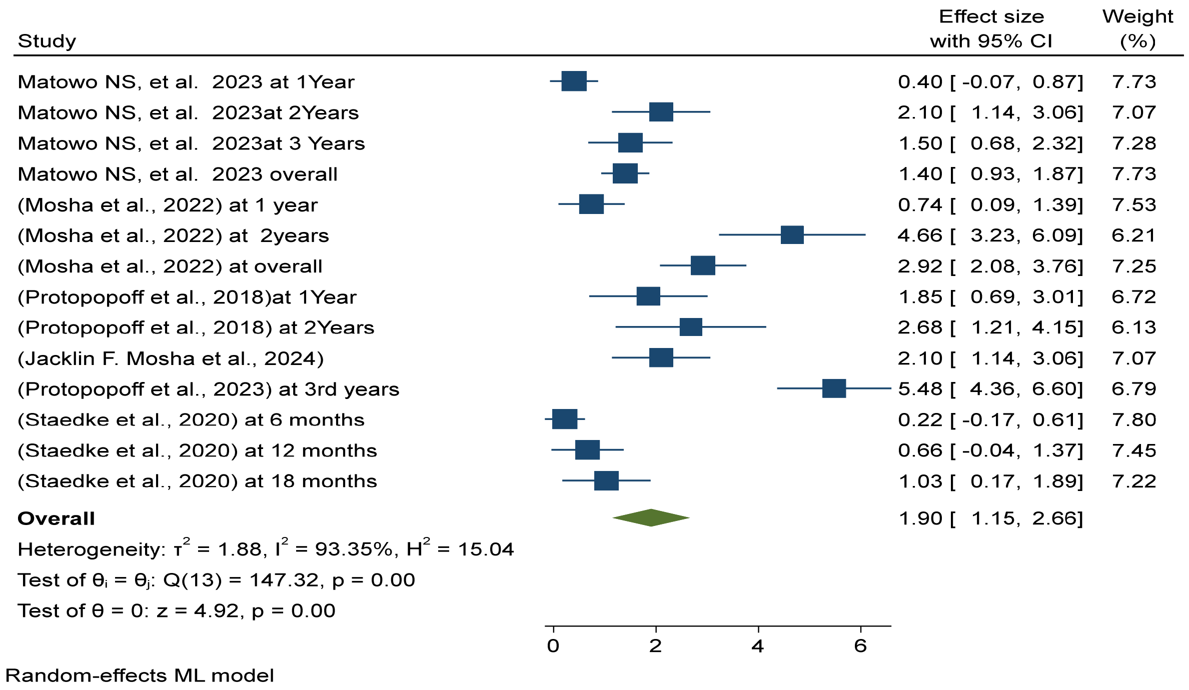

Figure S66: Forest plot shows post-intervention pooled mean indoor vector density per household per nights among Piperonyl butoxide long-lasting insecticidal nets intervention in Africa 2024

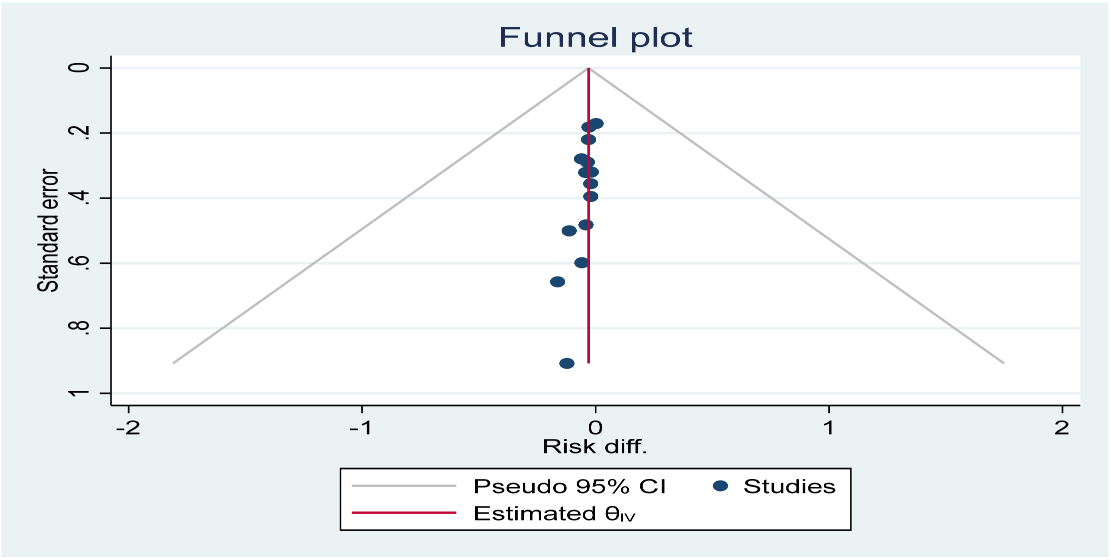

Figure S67: Funnel plot showing the distribution of included studies post-intervention pooled mean indoor vector density per household per nights among Piperonyl butoxide long-lasting insecticidal nets intervention in Africa 2024

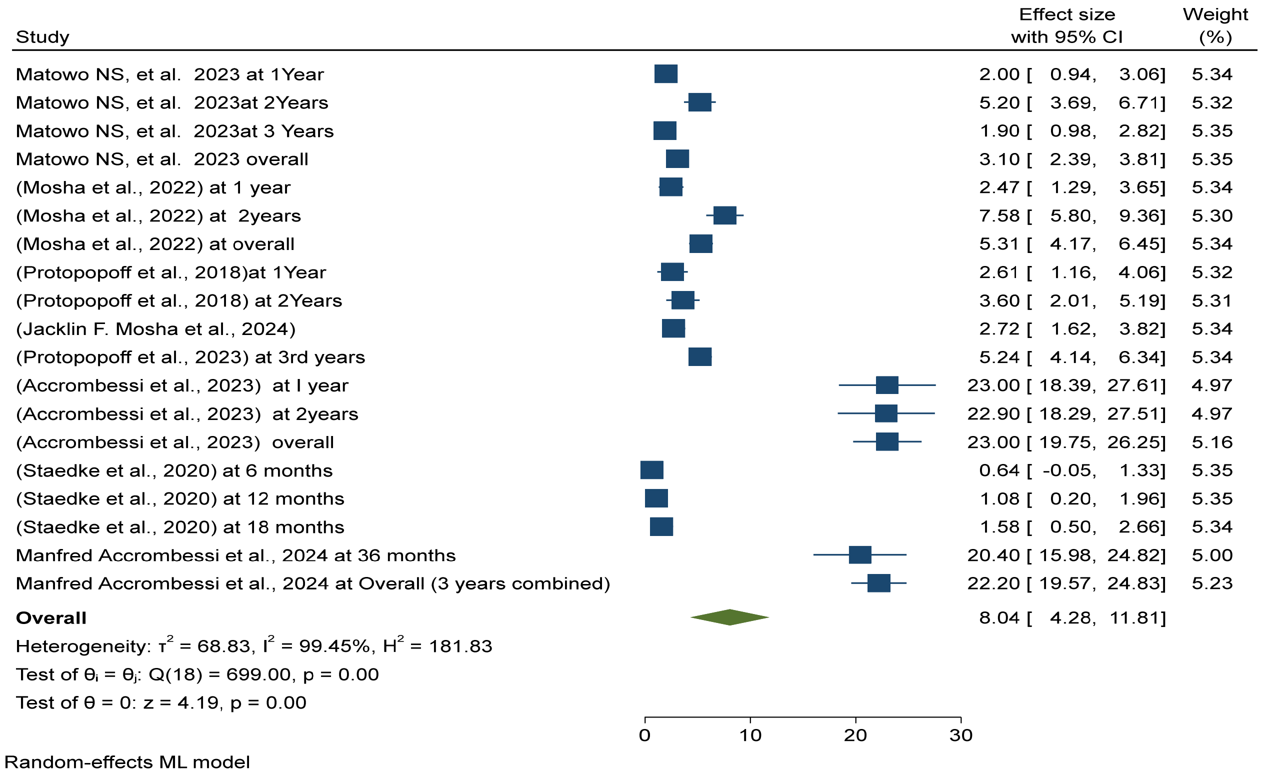

Figure S68: Forest plot shows post-intervention pooled mean indoor vector density per household per nights among pyrethroid-only long-lasting insecticidal nets intervention in Africa 2024

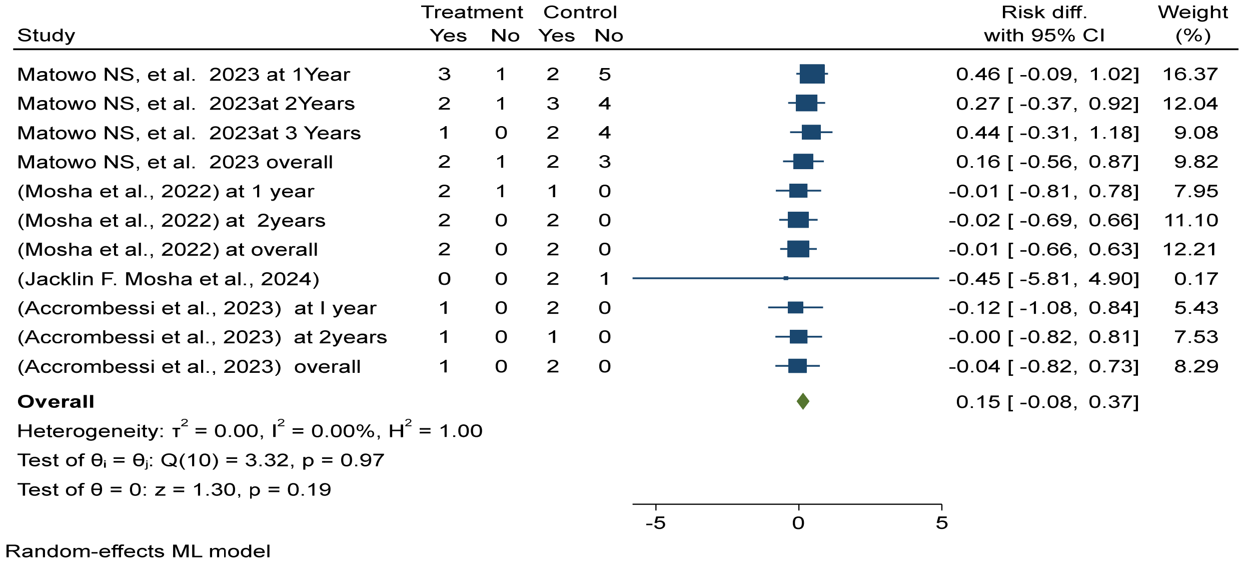

Figure S69: Forest plot shows post-intervention pooled effectiveness and efficacy of pyriproxyfen long-lasting insecticidal nets (LLINs) versus pyrethroid-only LLINs for sporozoite rate reduction in Africa 2024

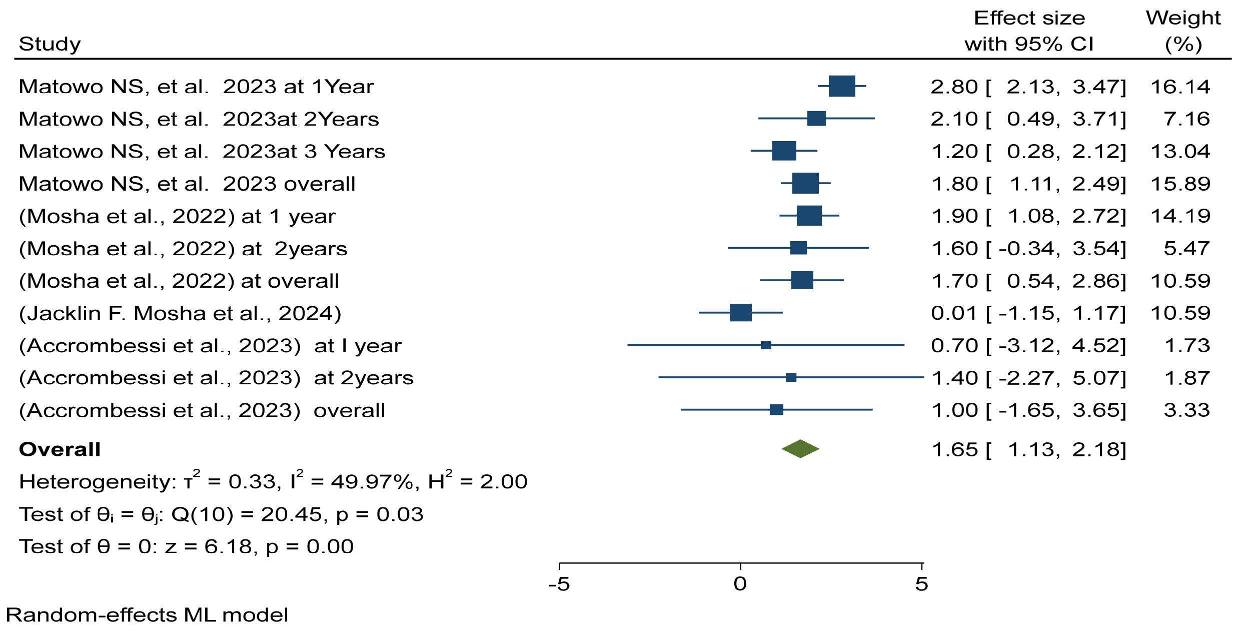

Figure S70: Forest plot shows post-intervention pooled prevalence of sporozoite rate among using pyriproxyfen long-lasting insecticidal nets (LLINs) versus pyrethroid-only LLINs for sporozoite rate reduction in Africa 2024

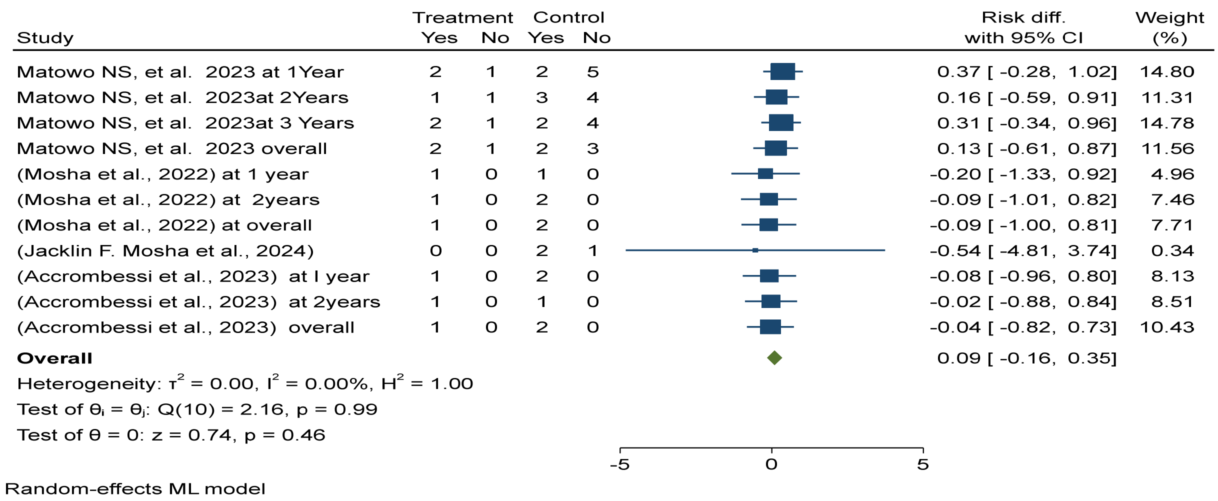

Figure S71: Forest plot shows post-intervention pooled effectiveness and efficacy of chlorfenapyr long-lasting insecticidal nets (LLINs) versus pyrethroid-only LLINs for sporozoite rate reduction in Africa 2024

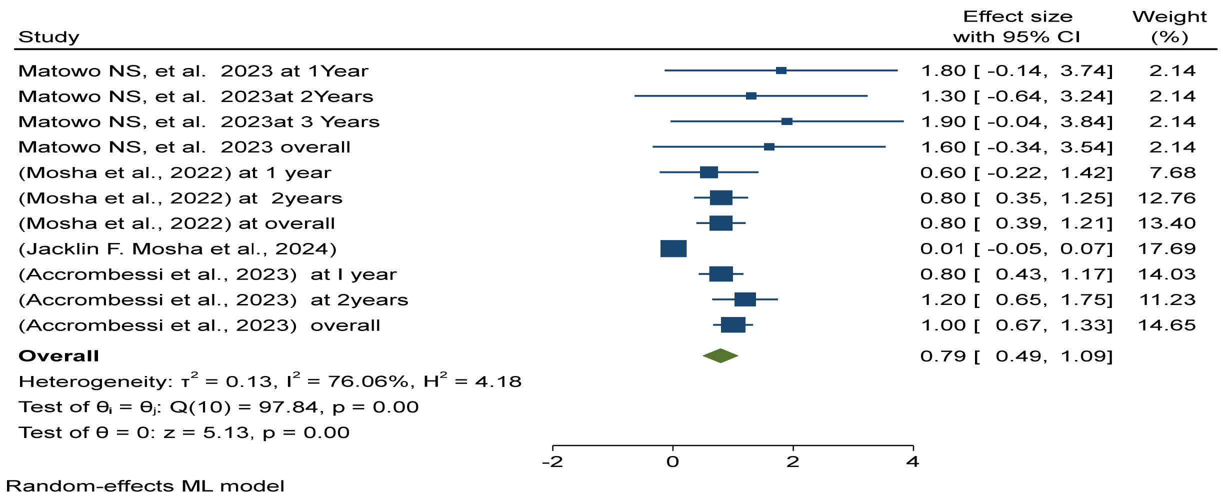

Figure S72: Forest plot shows post-intervention pooled prevalence of sporozoite rate among using chlorfenapyr long-lasting insecticidal nets (LLINs) versus pyrethroid-only LLINs for sporozoite rate reduction in Africa 2024

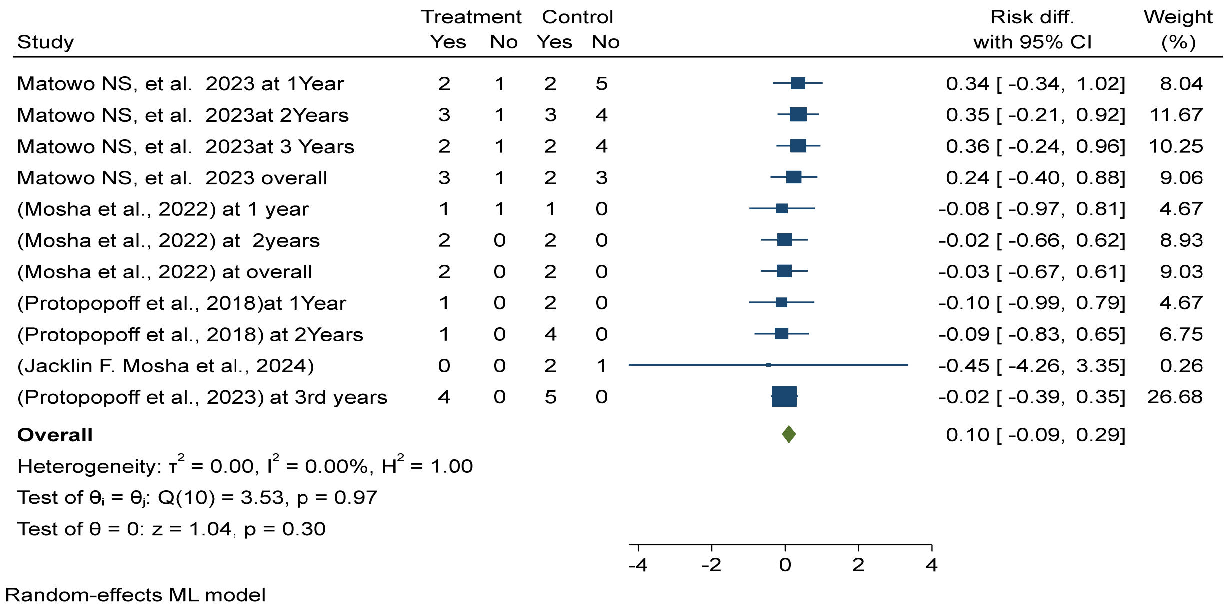

Figure S73: Forest plot shows post-intervention pooled effectiveness and efficacy of Piperonyl butoxide long-lasting insecticidal nets (LLINs) versus pyrethroid-only LLINs for sporozoite rate reduction in Africa 2024

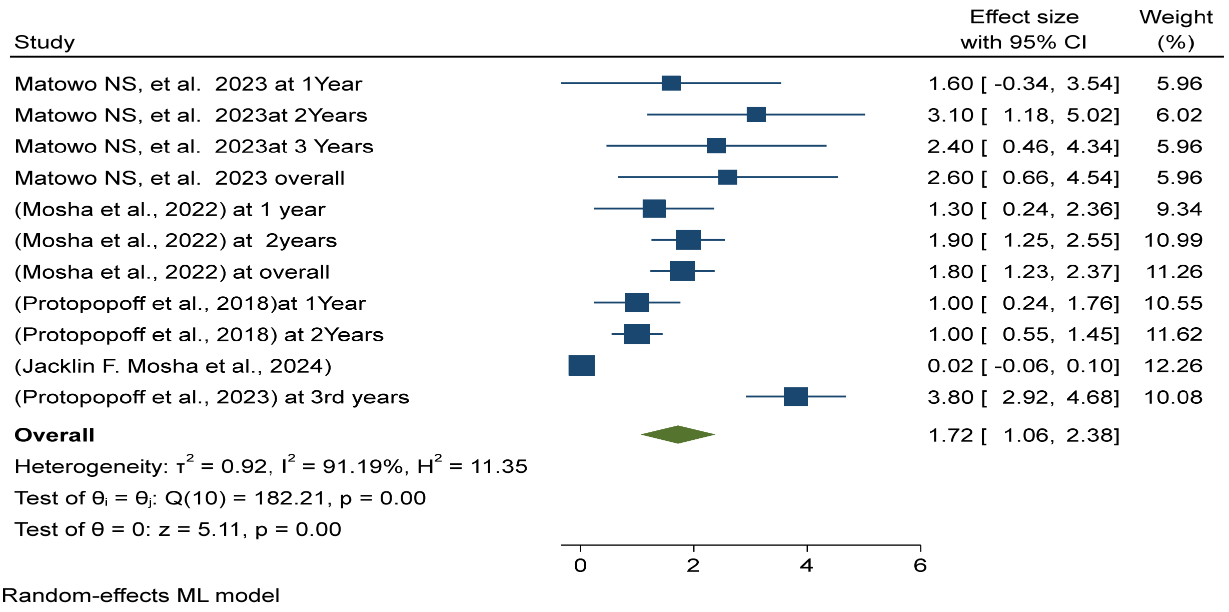

Figure S74: Forest plot shows post-intervention pooled prevalence of sporozoite rate among using Piperonyl butoxide long-lasting insecticidal nets (LLINs) versus pyrethroid-only LLINs for sporozoite rate reduction in Africa 2024.

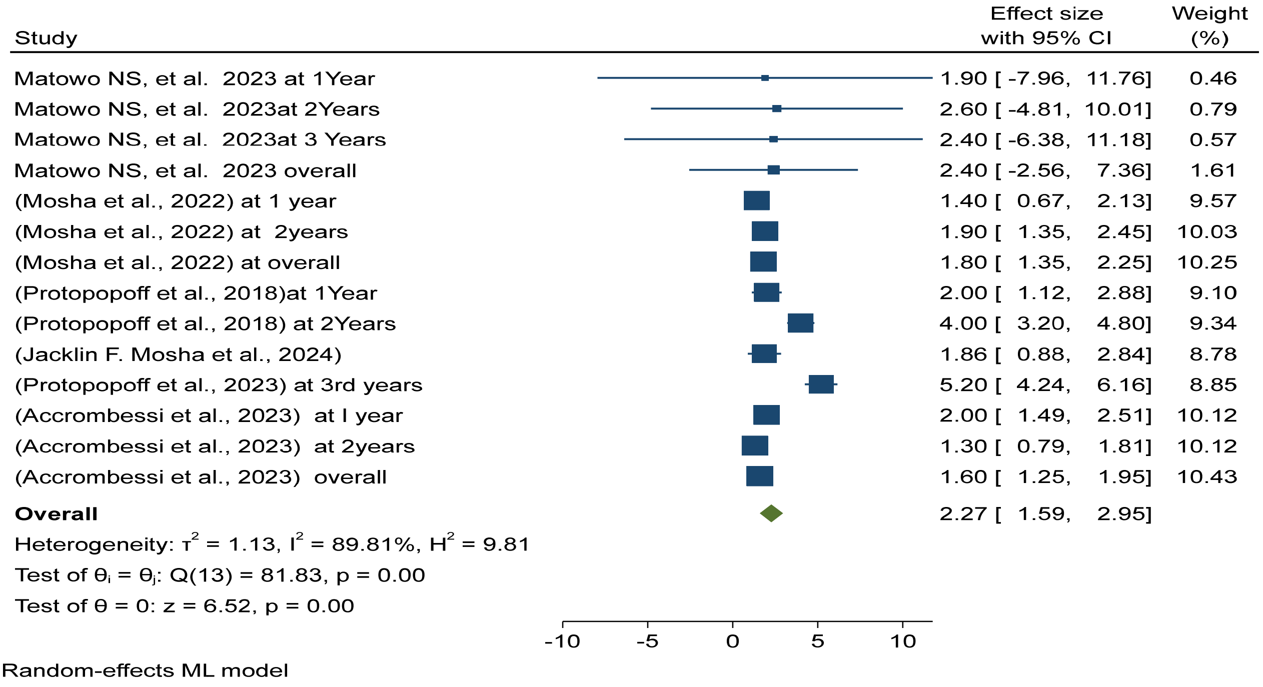

Figure S75: Forest plot shows post-intervention pooled prevalence of sporozoite rate among using pyrethroid-only long-lasting insecticidal nets (LLINs) for sporozoite rate reduction in Africa 2024

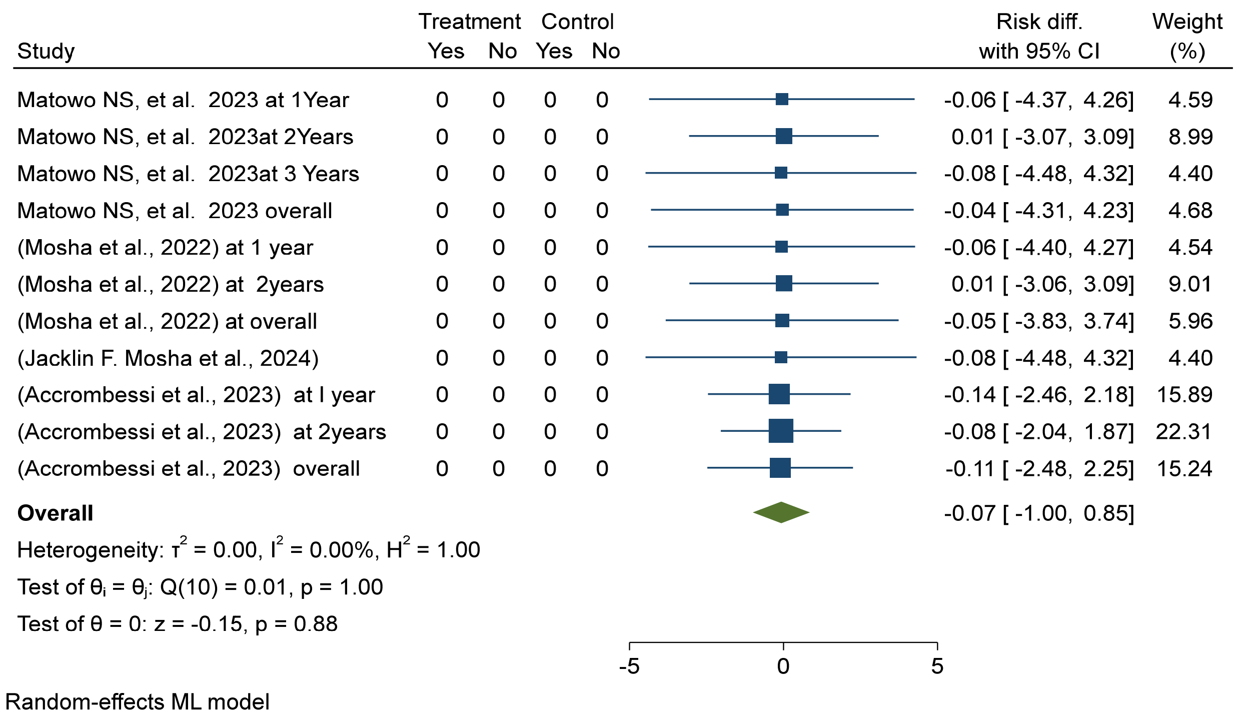

Figure S76: Forest plot shows post-intervention pooled effectiveness and efficacy of Pyriproxyfen long-lasting insecticidal nets (LLINs) versus pyrethroid-only LLINs for mean entomological inoculation rate reduction in Africa 2024

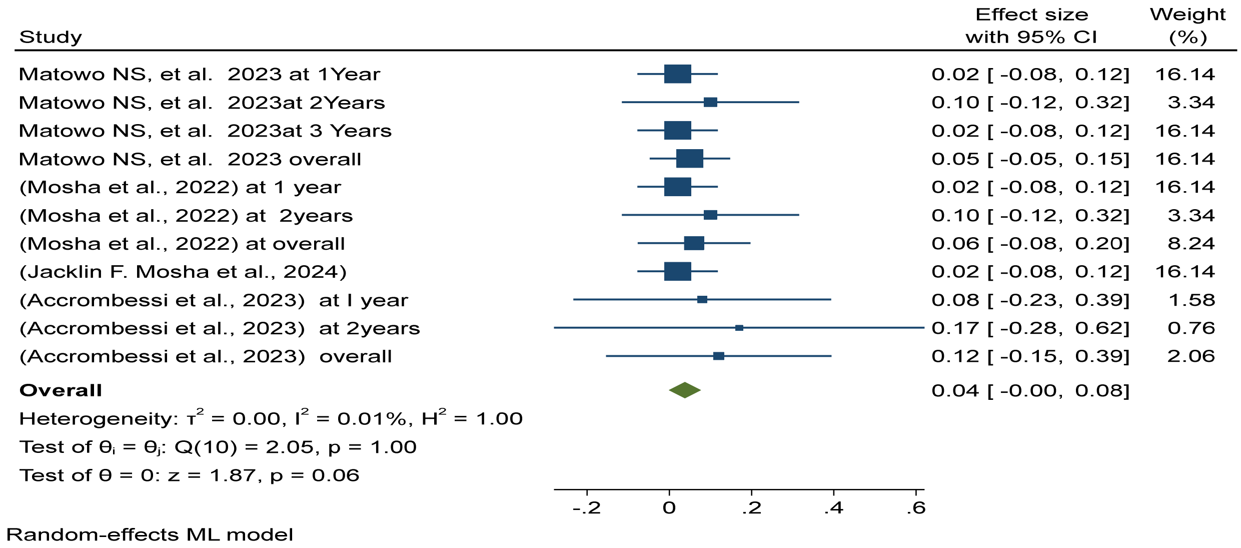

Figure S77: Forest plot shows post-intervention pooled mean entomological inoculation rate per household per night among using the Pyriproxyfen long-lasting insecticidal nets (LLINs) for sporozoite rate reduction in Africa 2024.

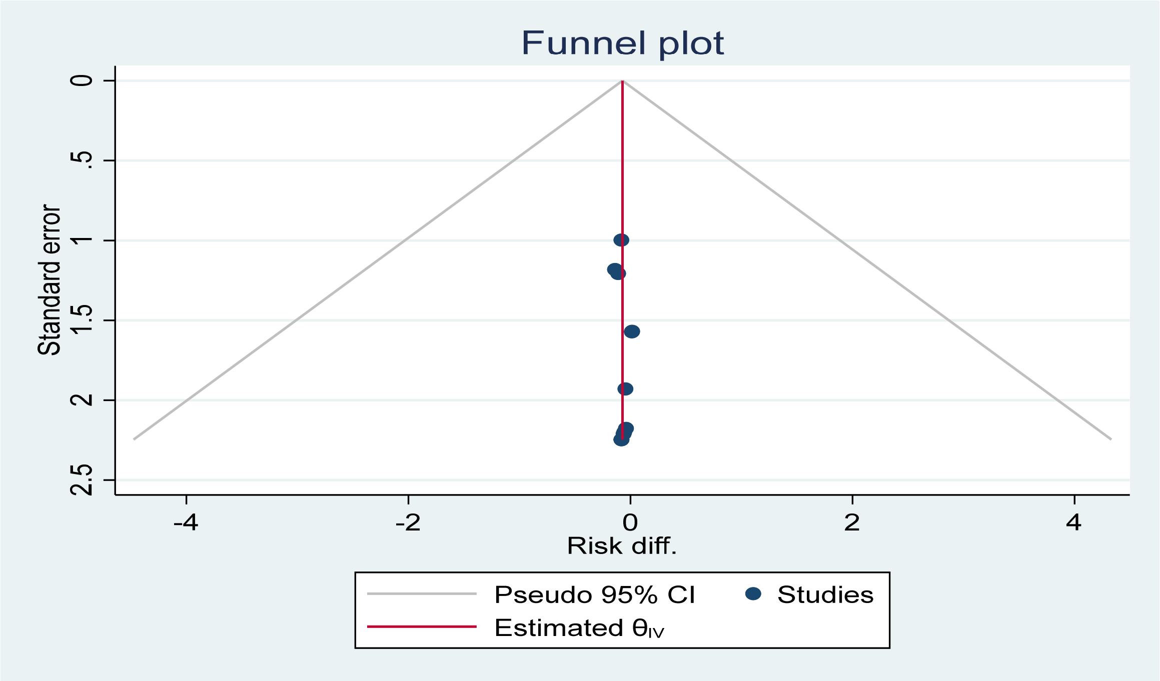

Figure S78: Funnel plot showing the distribution of included studies post-intervention pooled mean entomological inoculation rate per household per nights among using Pyriproxyfen long-lasting insecticidal nets intervention in Africa 2024

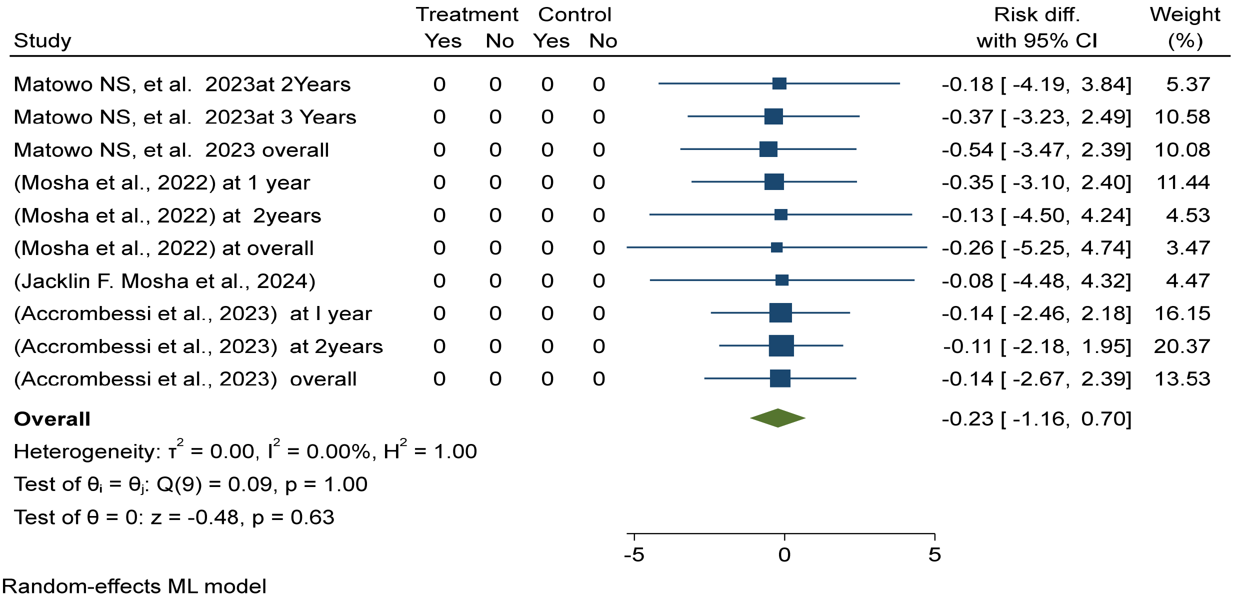

Figure S79: Forest plot shows post-intervention pooled effectiveness and efficacy of chlorfenapyr long-lasting insecticidal nets (LLINs) versus pyrethroid-only LLINs for mean entomological inoculation rate reduction in Africa 2024

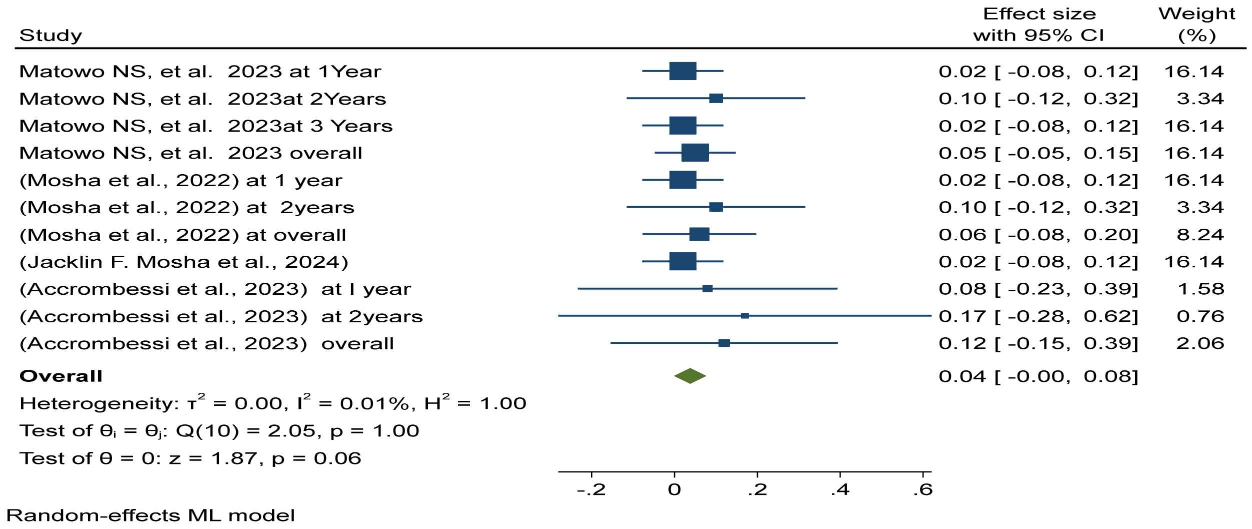

Figure S80: Forest plot shows post-intervention pooled mean entomological inoculation rate per household per night among using the chlorfenapyr long-lasting insecticidal nets (LLINs) for sporozoite rate reduction in Africa 2024.

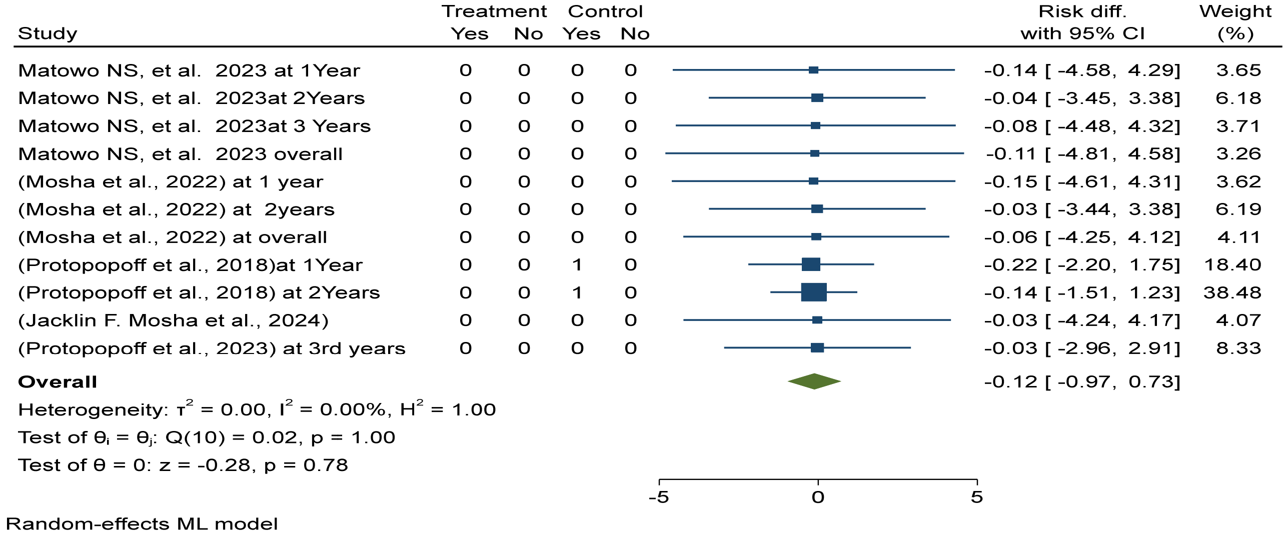

Figure S81: Forest plot shows post-intervention pooled effectiveness and efficacy of Piperonyl butoxide long-lasting insecticidal nets (LLINs) versus pyrethroid-only LLINs for mean entomological inoculation rate reduction in Africa 2024

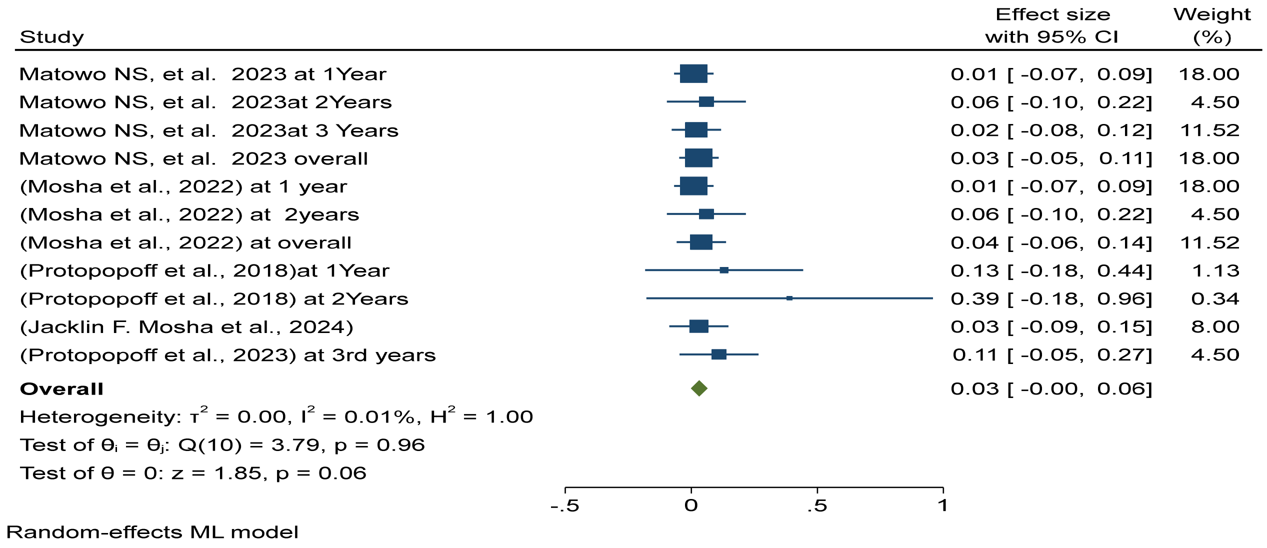

Figure S82: Forest plot shows post-intervention pooled mean entomological inoculation rate per household per night among using the Piperonyl butoxide long-lasting insecticidal nets (LLINs) in Africa 2024.

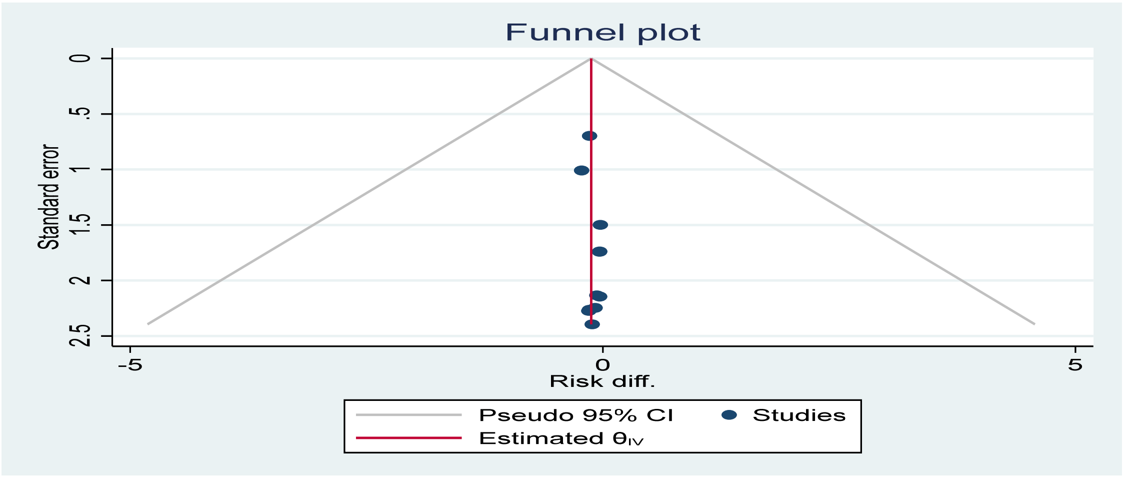

Figure S83: Funnel plot showing the distribution of included studies pooled mean entomological inoculation rate per household per night among using piperonyl butoxide long-lasting insecticidal nets (LLINs) versus pyrethroid-only LLINs for malaria control in Africa in 2024.

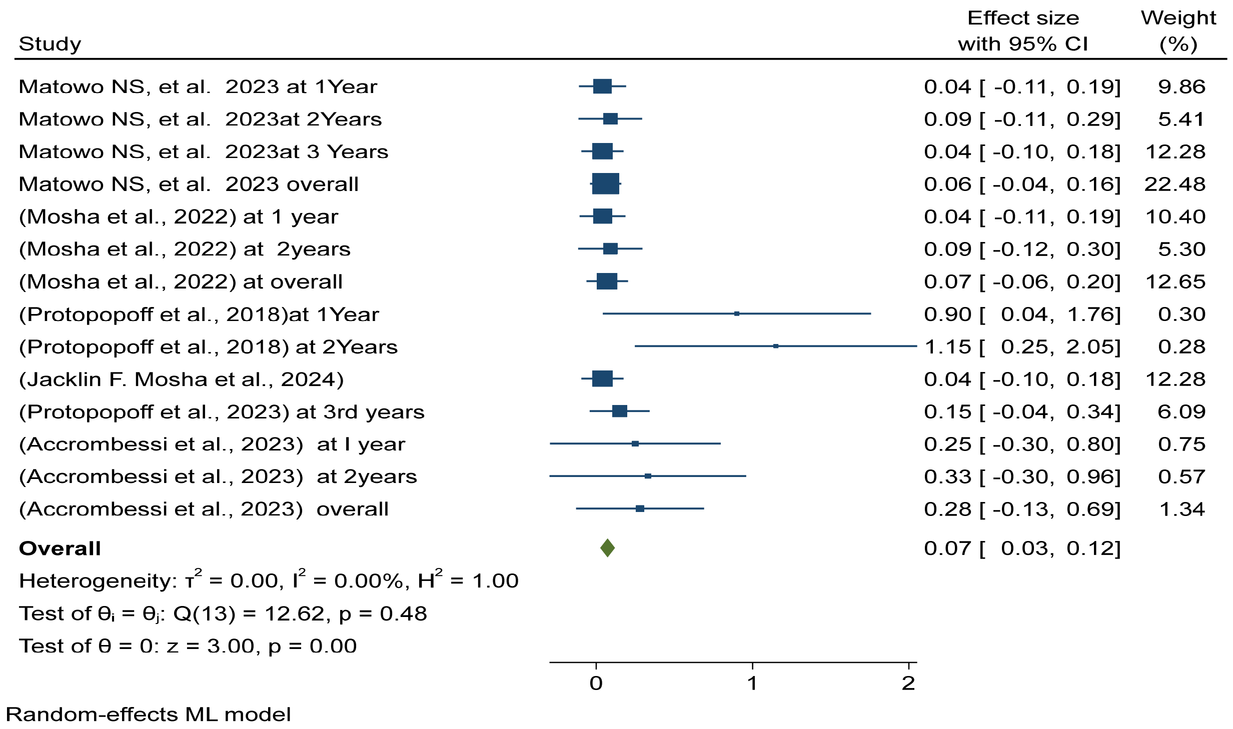

Figure S84: Forest plot shows post-intervention pooled mean entomological inoculation rate per household per night among using the Pyrethroid-only long-lasting insecticidal nets (LLINs) in Africa 2024.

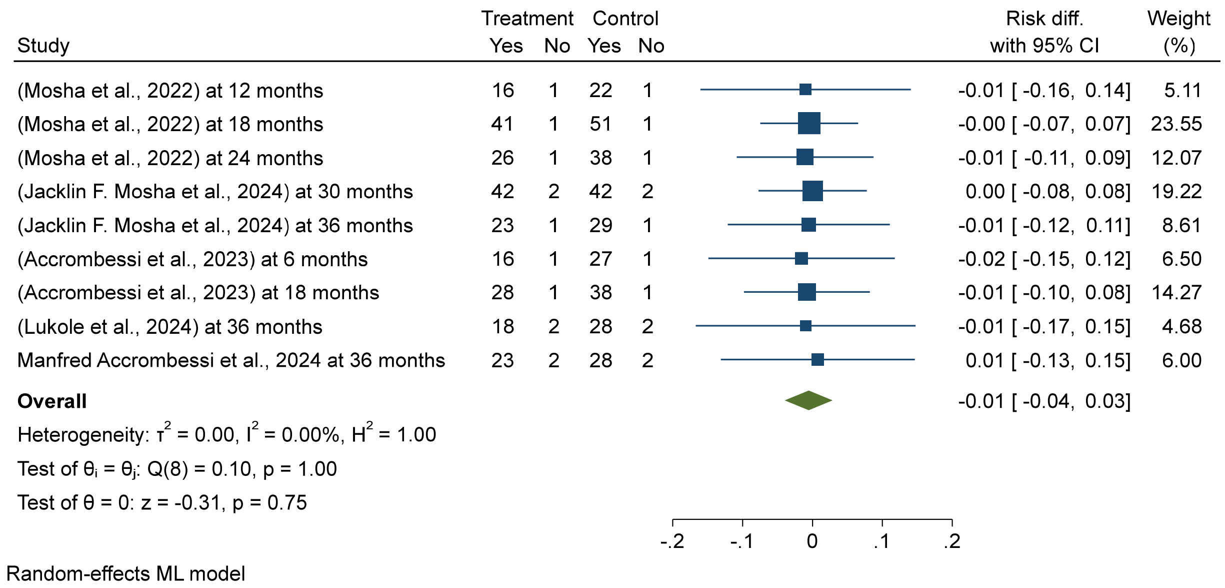

Figure S85: Forest plot shows pooled post-intervention effectiveness and efficacy of chlorfenapyr long-lasting insecticidal nets (LLINs) versus pyriproxyfen LLINs malaria infection reduction in Africa 2024

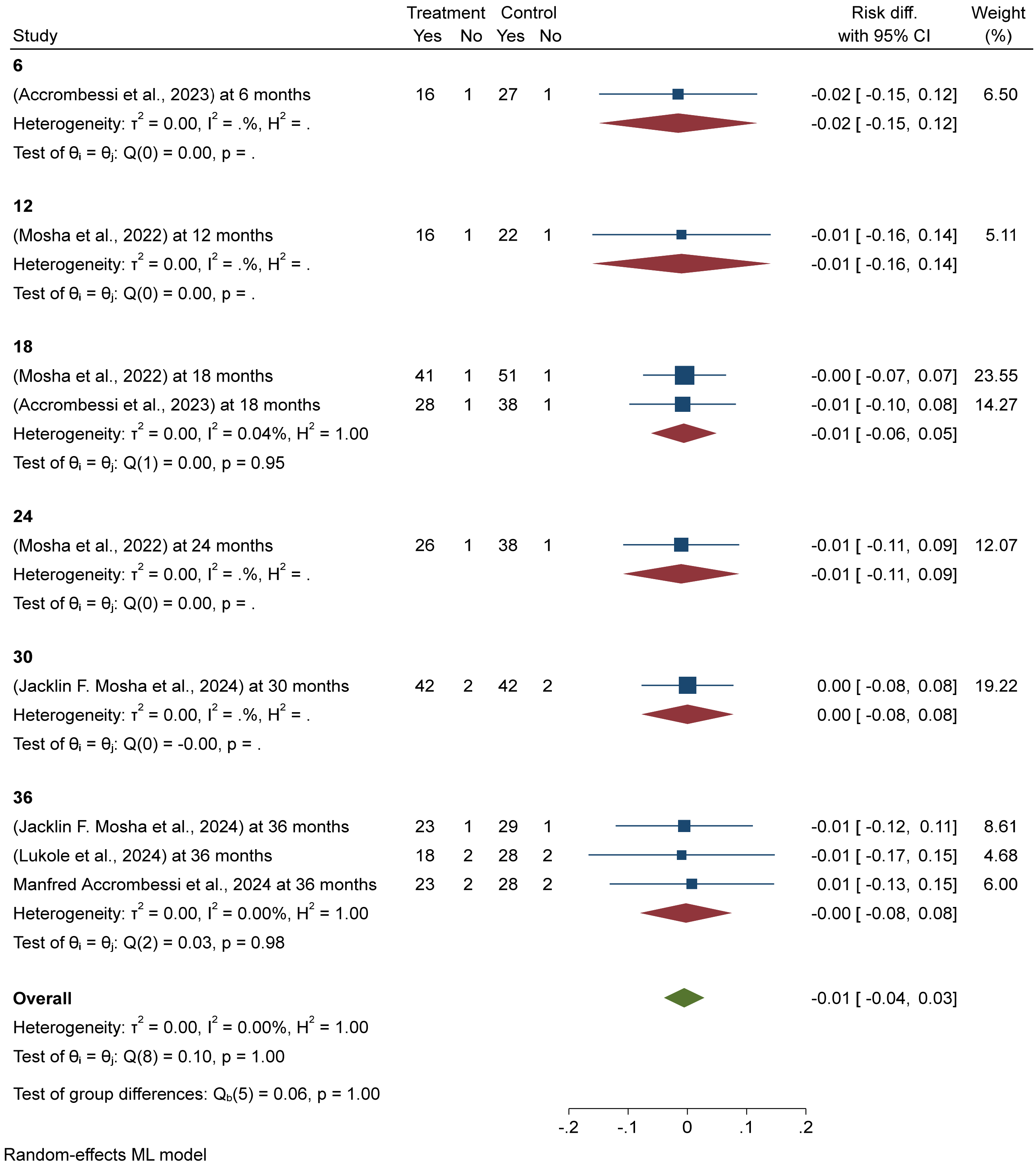

Figure S86: Forest plot shows subgroup analysis of post-intervention follow up effectiveness and efficacy of chlorfenapyr long-lasting insecticidal nets (LLINs) versus pyriproxyfen LLINs malaria infection reduction in Africa 2024

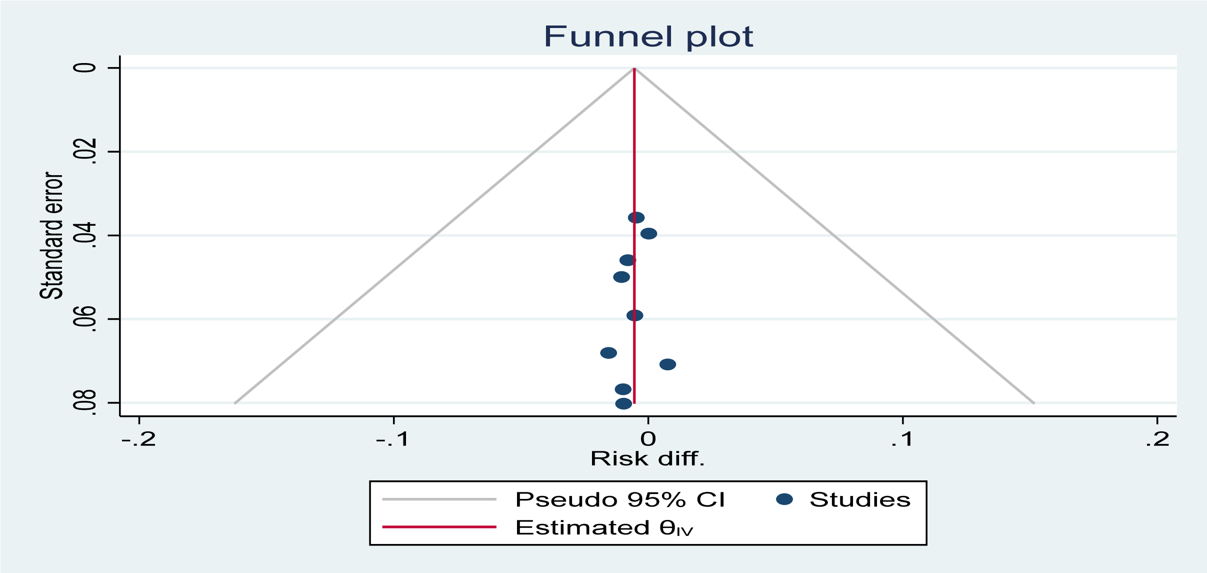

Figure S87**:** Funnel plot showing the distribution of included studies pooled malaria infection risk reduction among children using chlorfenapyr long-lasting insecticidal nets (LLINs) versus pyriproxyfen LLINs for malaria control in Africa in 2024.

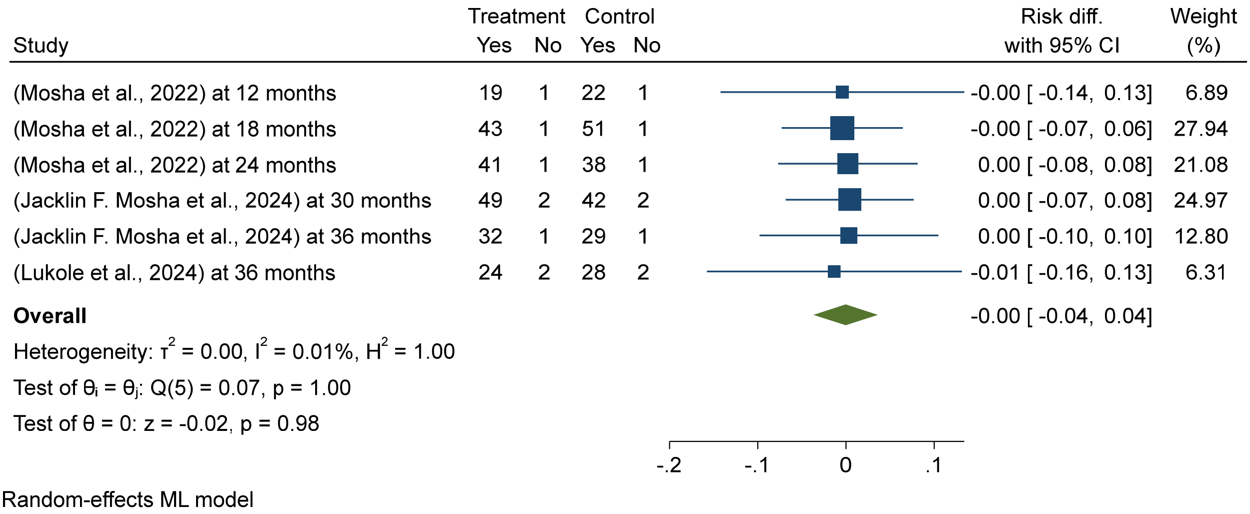

Figure S88: Forest plot shows pooled post-intervention effectiveness and efficacy of Piperonyl butoxide long-lasting insecticidal nets (LLINs) versus pyriproxyfen LLINs malaria infection reduction in Africa 2024

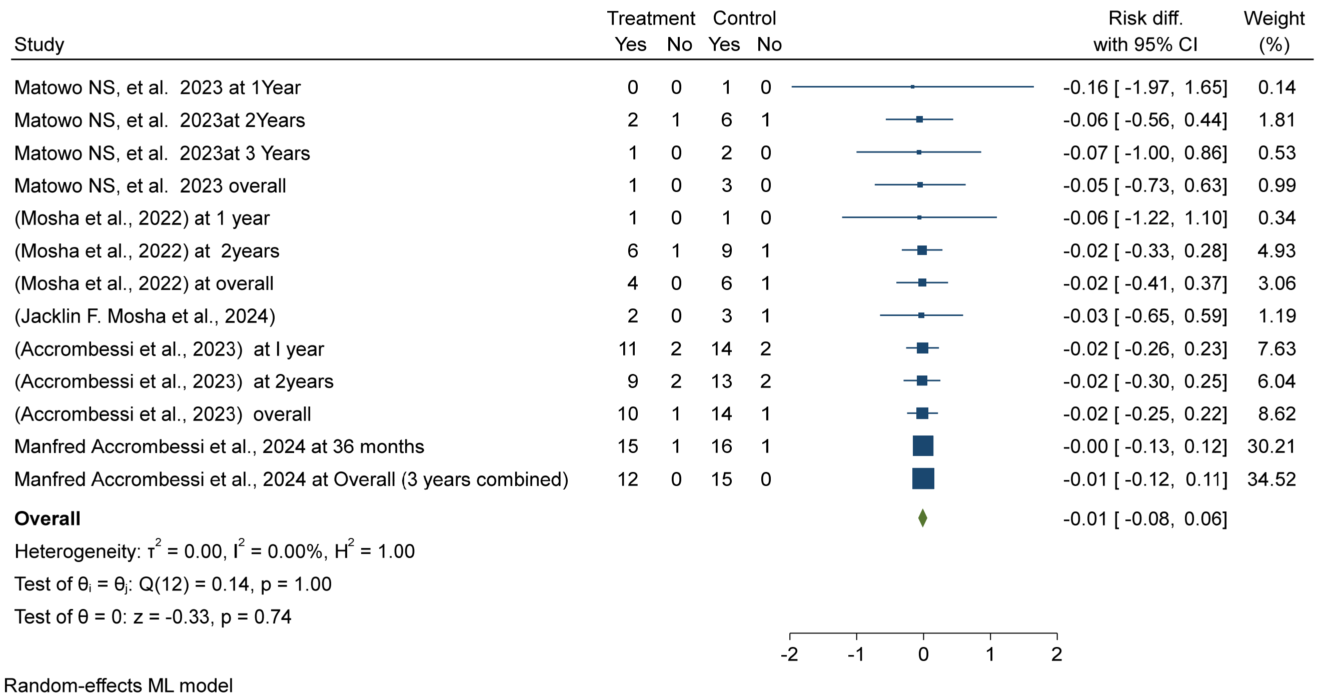

Figure S89: Forest plot shows pooled post-intervention effectiveness and efficacy of chlorfenapyr versus pyriproxyfen long-lasting insecticidal nets (LLINs) reduce mean indoor vector density per household per night in Africa 2024

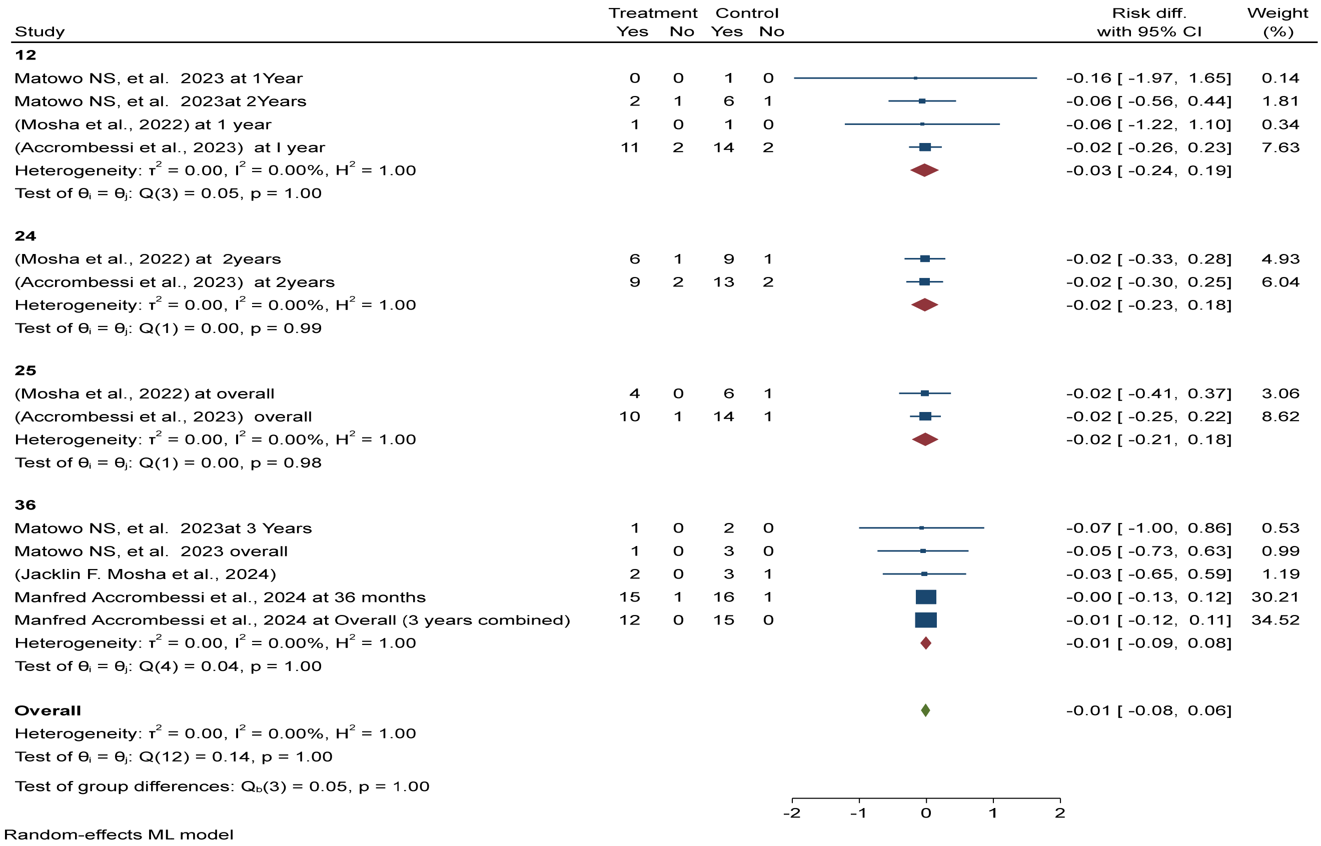

Figure S90: Forest plot shows subgroup analysis post-intervention follow-up effectiveness and efficacy of chlorfenapyr versus pyriproxyfen long-lasting insecticidal nets (LLINs) reduce mean indoor vector density per household per night in Africa 2024

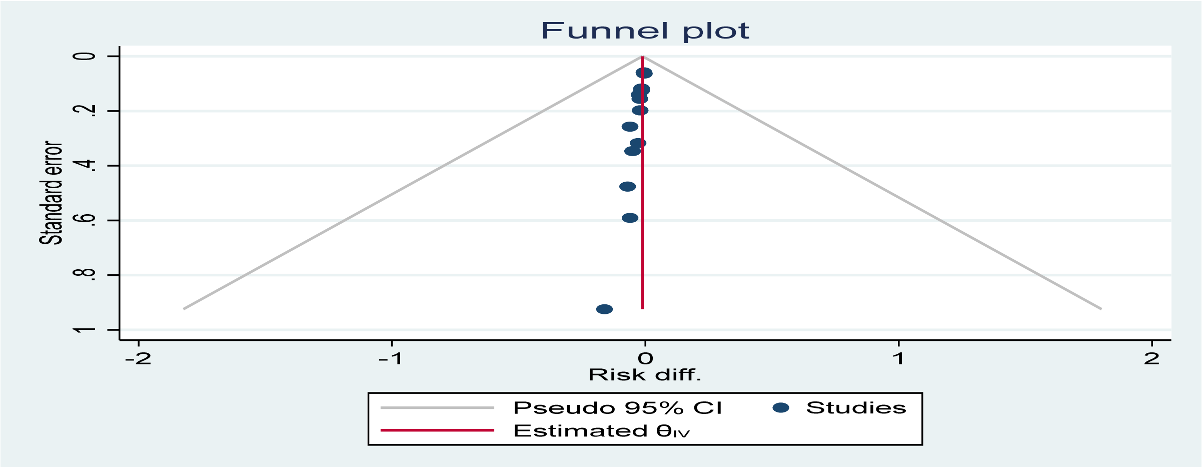

Figure S91: Funnel plot showing the distribution of included studies pooled mean indoor vector density per household per night among using chlorfenapyr long-lasting insecticidal nets (LLINs) versus pyriproxyfen LLINs for malaria control in Africa in 2024

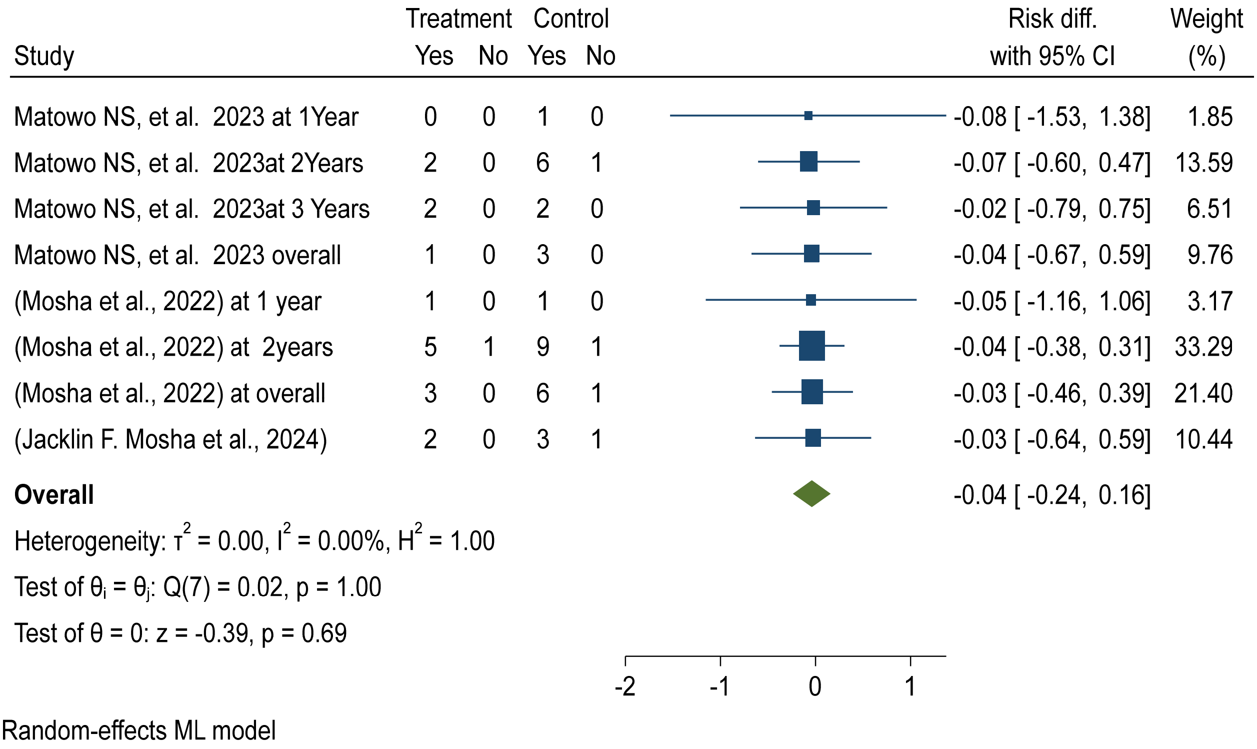

Figure S92: Forest plot shows post-intervention effectiveness and efficacy of Piperonyl butoxide versus pyriproxyfen long-lasting insecticidal nets (LLINs) reduce mean indoor vector density per household per night in Africa 2024

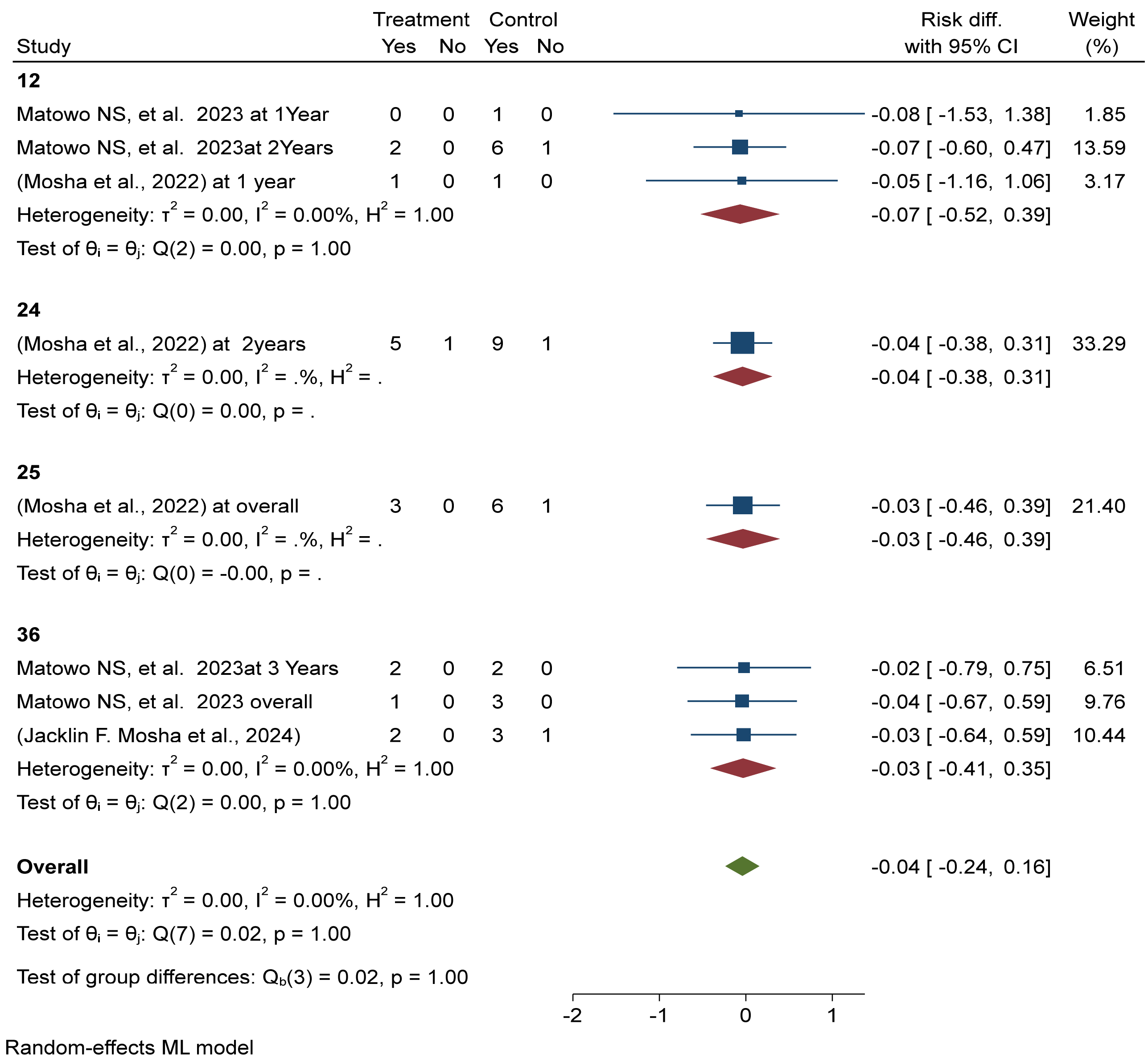

Figure S93: Forest plot shows subgroup analysis post-intervention follow-up effectiveness and efficacy of Piperonyl butoxide versus pyriproxyfen long-lasting insecticidal nets (LLINs) reduce mean indoor vector density per household per night in Africa 2024

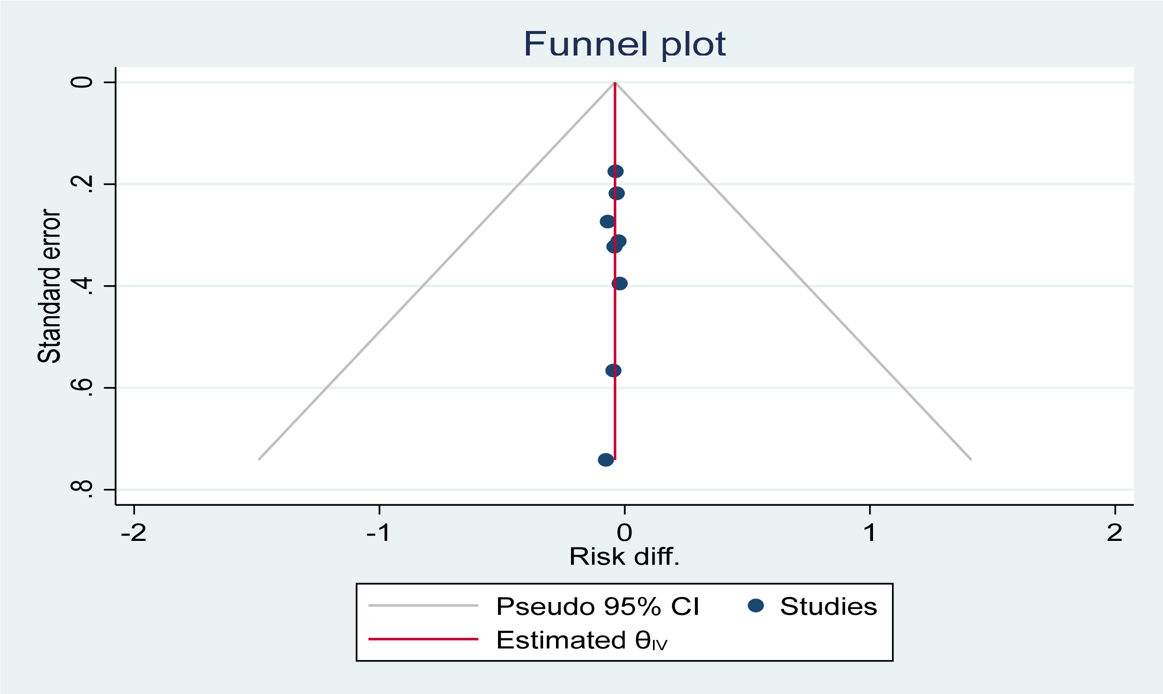

Figure S94: Funnel plot showing the distribution of included studies pooled mean indoor vector density per household per night among using Piperonyl butoxide long-lasting insecticidal nets (LLINs) versus pyriproxyfen LLINs for malaria control in Africa in 2024

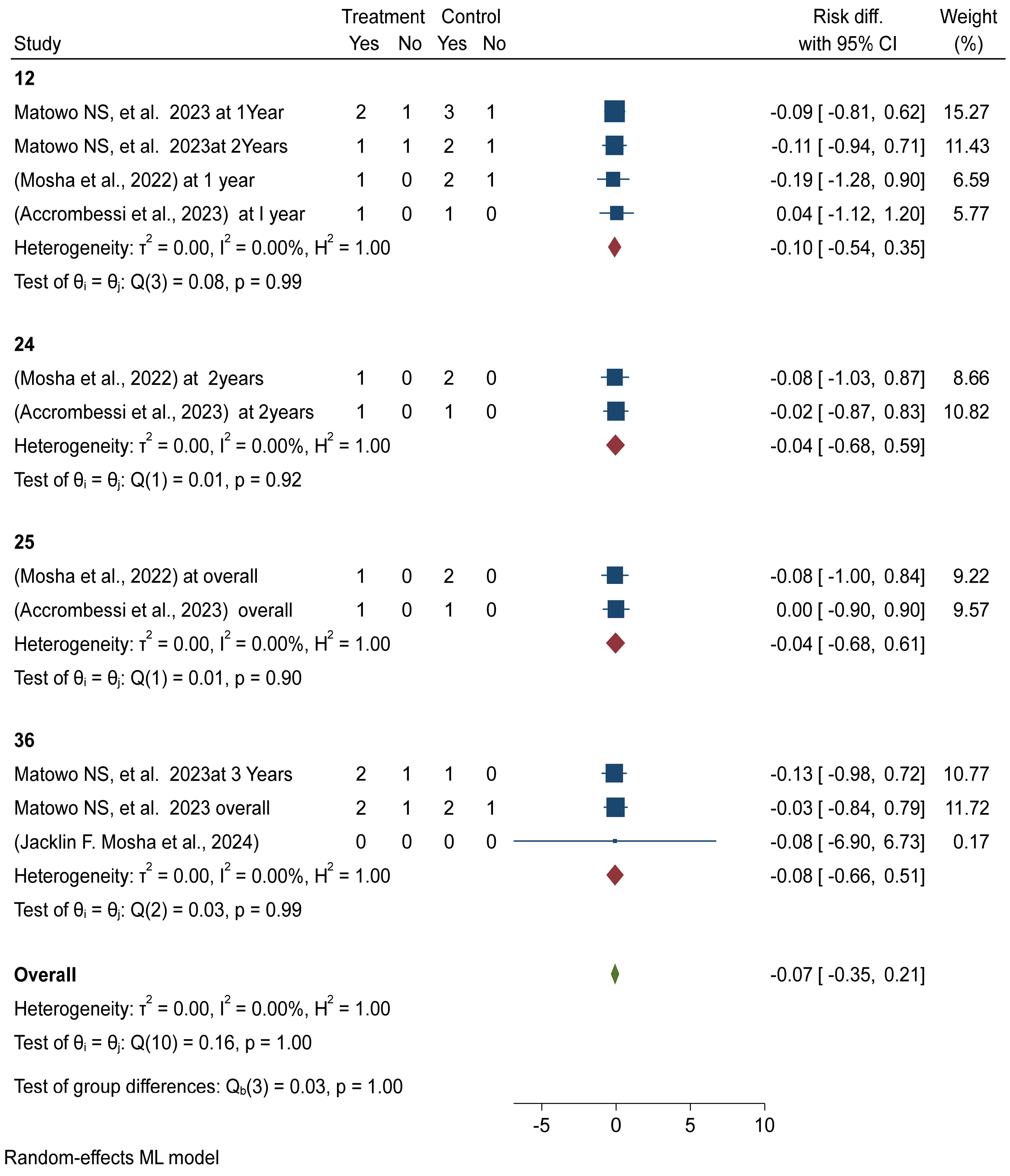

Figure S95: Forest plot shows subgroup analysis post-intervention follow-up pooled post-intervention effectiveness and efficacy of chlorfenapyr versus pyriproxyfen long-lasting insecticidal nets (LLINs) reduce pooled sporozoite rate in Africa 2024

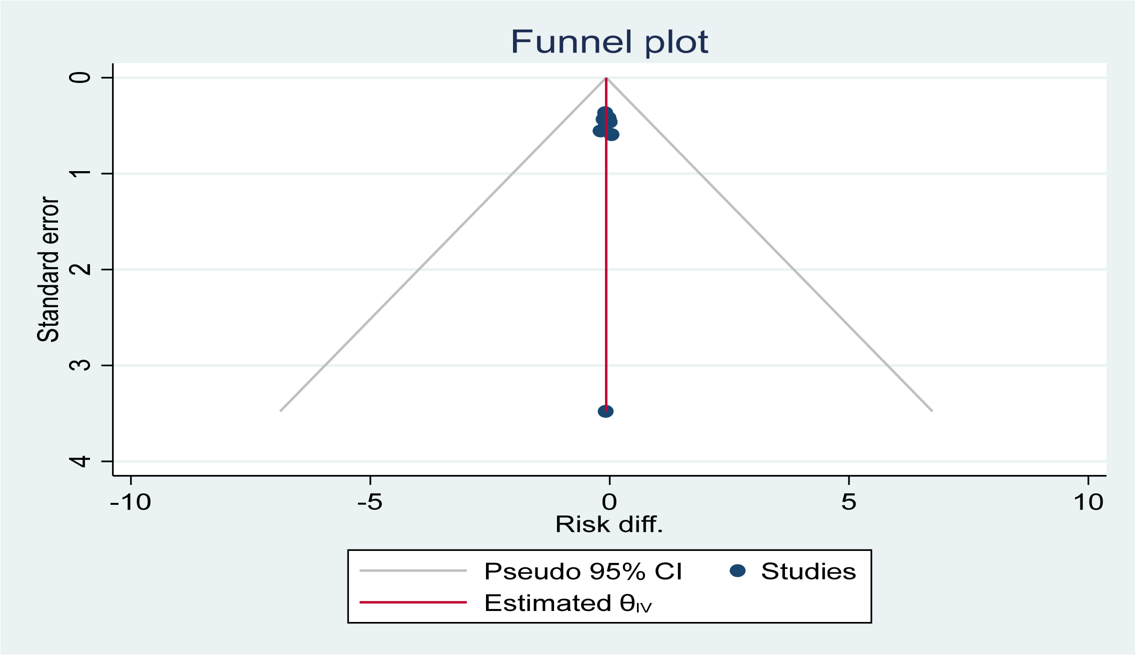

Figure S96: Funnel plot showing the distribution of included studies pooled sporozoite rate among using piperonyl butoxide long-lasting insecticidal nets (LLINs) versus pyriproxyfen LLINs for malaria control in Africa in 2024.

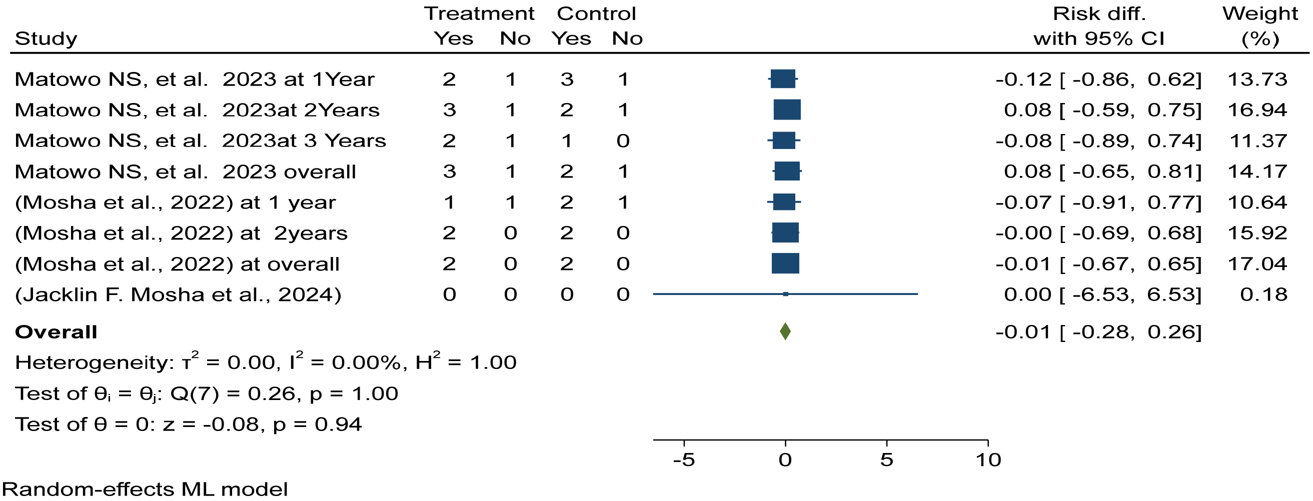

Figure S97: Forest plot shows pooled post-intervention effectiveness and efficacy of Piperonyl butoxid versus pyriproxyfen long-lasting insecticidal nets (LLINs) reduce pooled sporozoite rate in Africa 2024

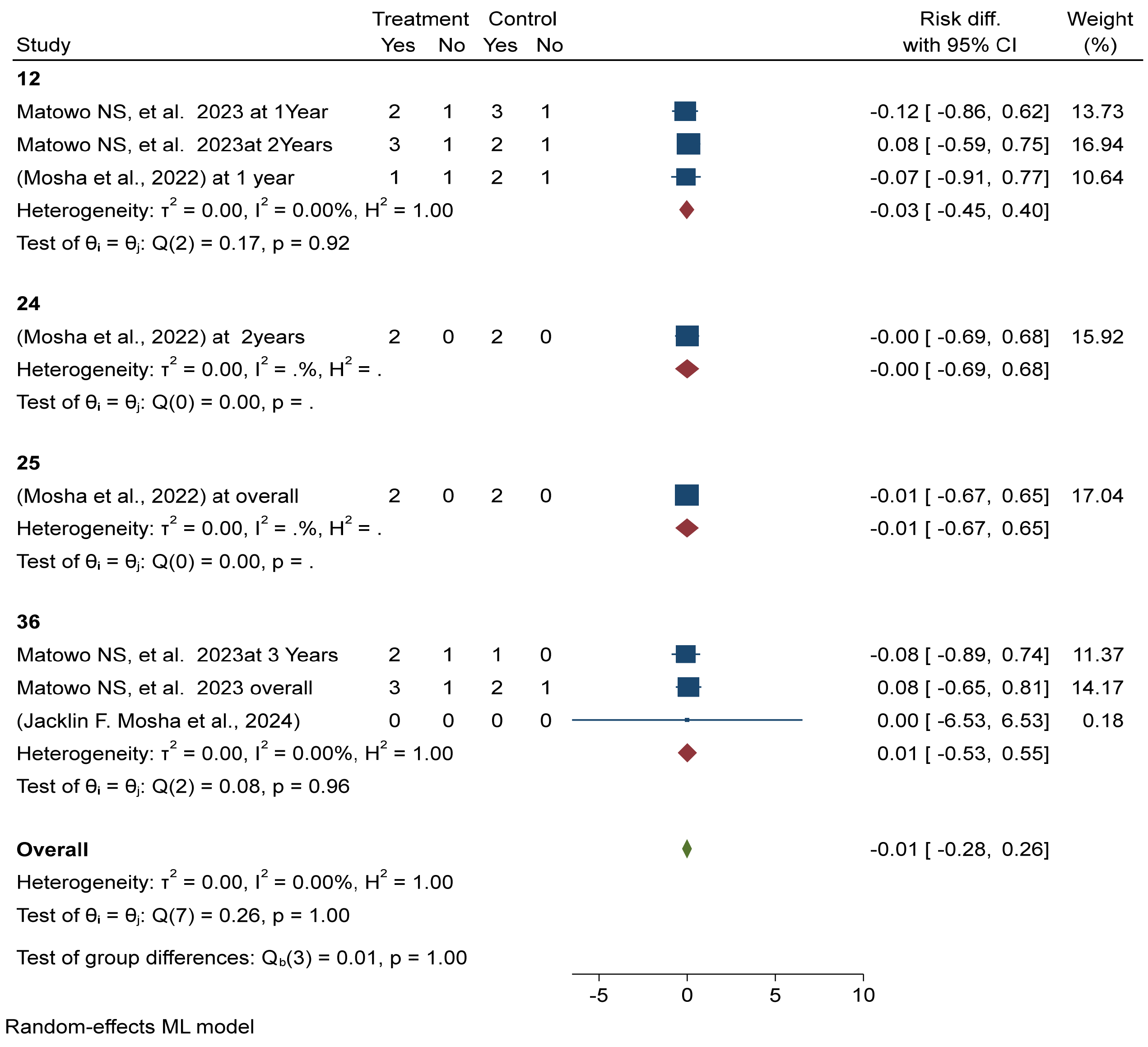

Figure S98: Forest plot shows subgroup analysis of post-intervention follow-up effectiveness and efficacy of Piperonyl butoxid versus pyriproxyfen long-lasting insecticidal nets (LLINs) reduce pooled sporozoite rate in Africa 2024

.

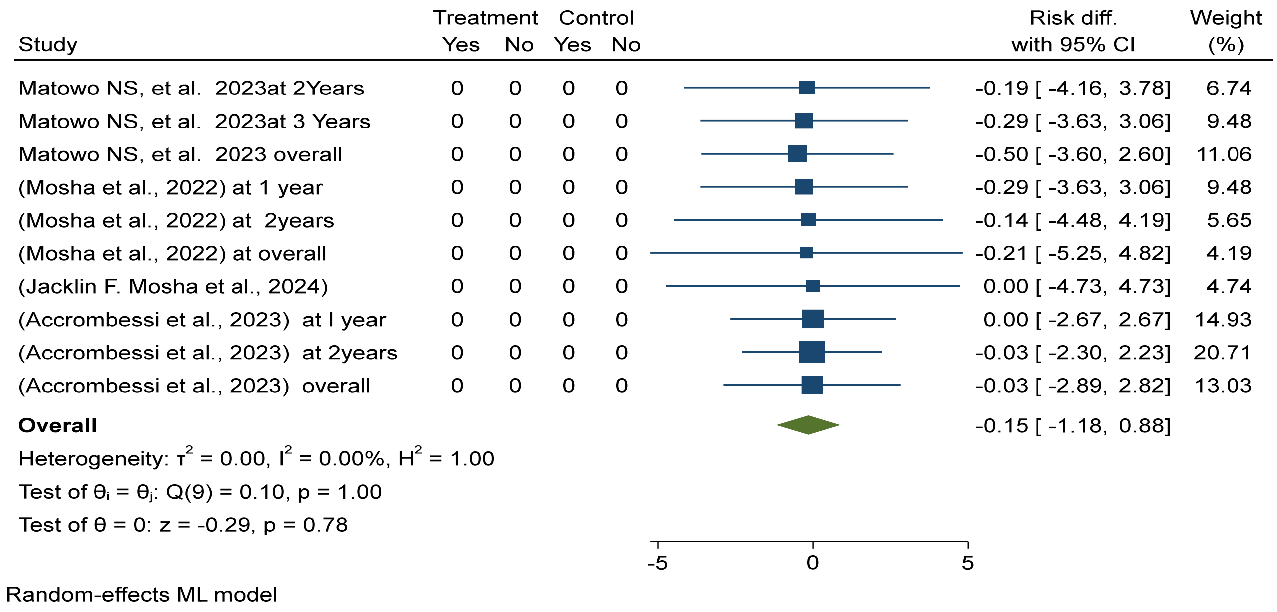

Figure S99: Forest plot shows pooled post-intervention effectiveness and efficacy of chlorfenapyr versus pyriproxyfen long-lasting insecticidal nets (LLINs) reduce pooled mean entomological inoculation rate per household per night in Africa 2024

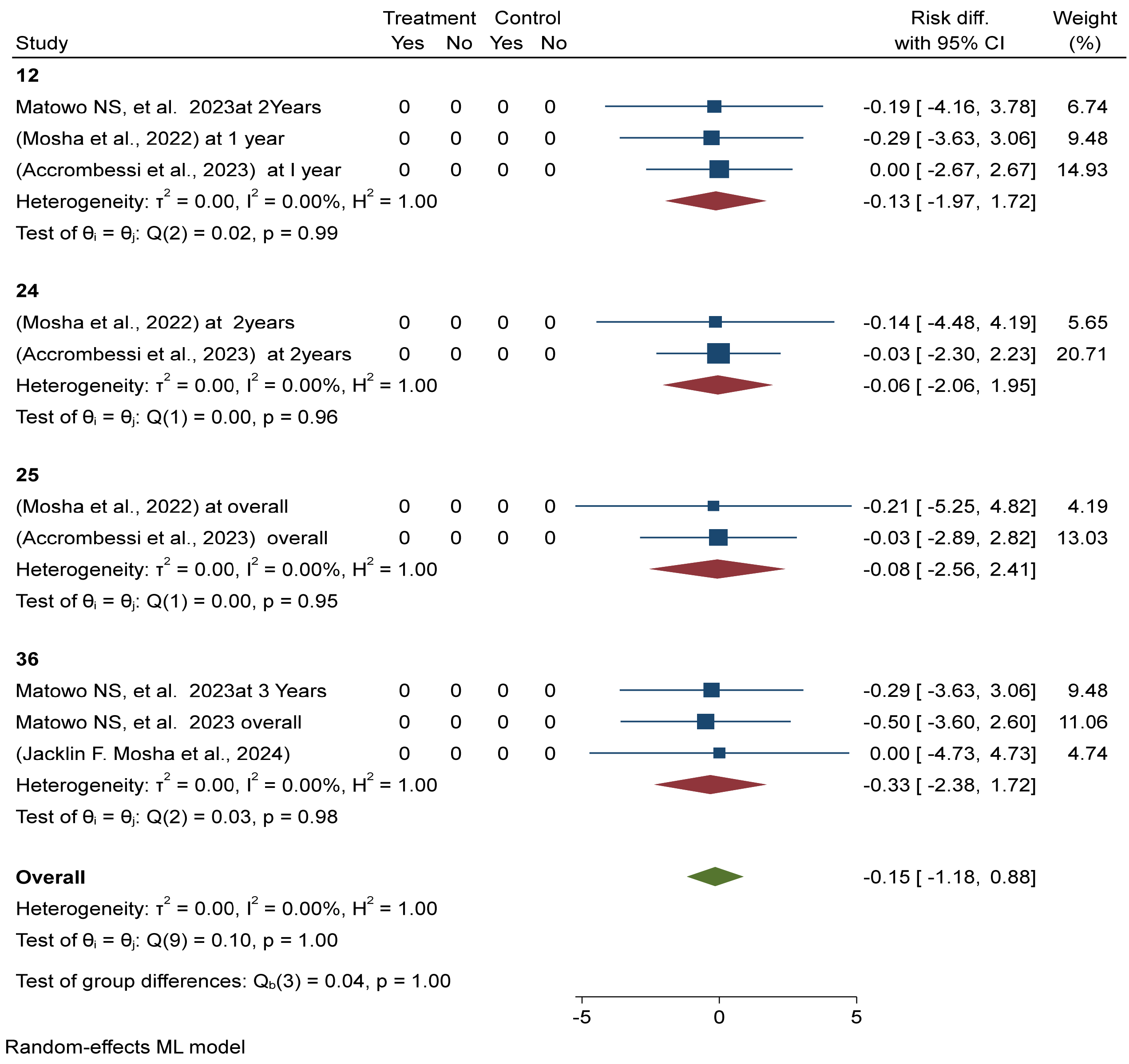

Figure S100: Forest plot shows subgroup analysis of post-intervention follow-up effectiveness and efficacy of chlorfenapyr versus pyriproxyfen long-lasting insecticidal nets (LLINs) reduce pooled mean entomological inoculation rate per household per night in Africa 2024

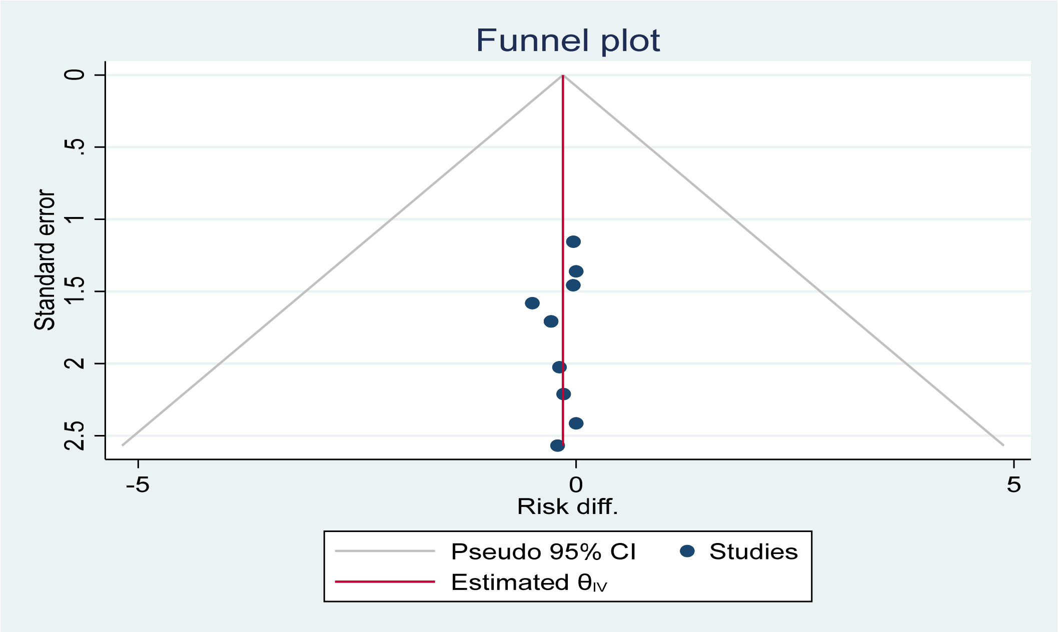

Figure S101: Funnel plot showing the distribution of included studies pooled mean entomological inoculation rate per household per night among using chlorfenapyr long-lasting insecticidal nets (LLINs) versus pyriproxyfen LLINs for malaria control in Africa in 2024.

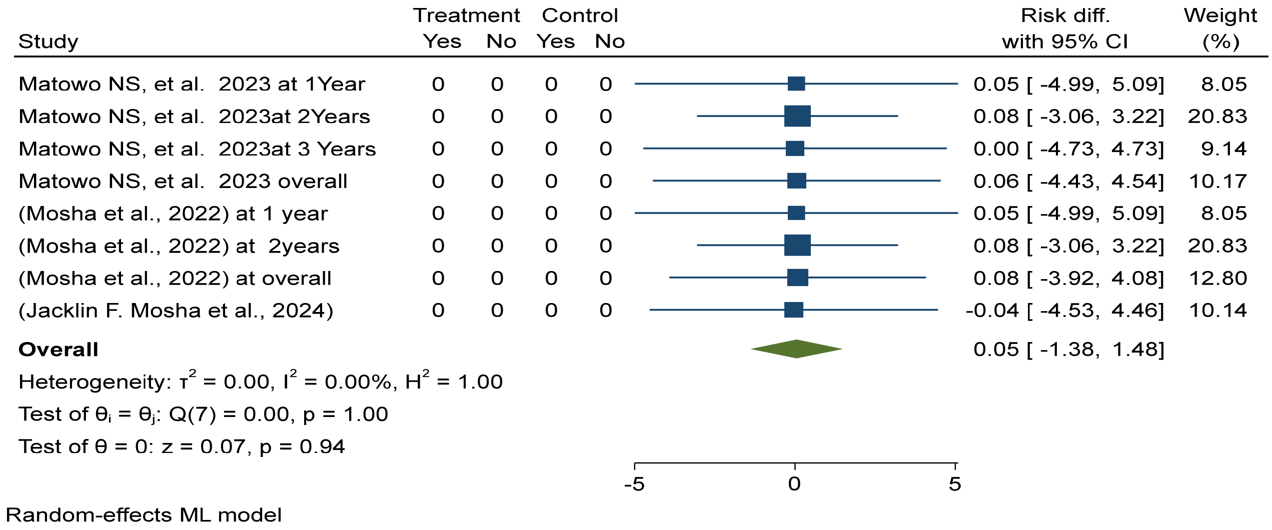

Figure S102: Forest plot shows pooled post-intervention effectiveness and efficacy of Piperonyl butoxide versus pyriproxyfen long-lasting insecticidal nets (LLINs) reduce pooled mean entomological inoculation rate per household per night in Africa 2024

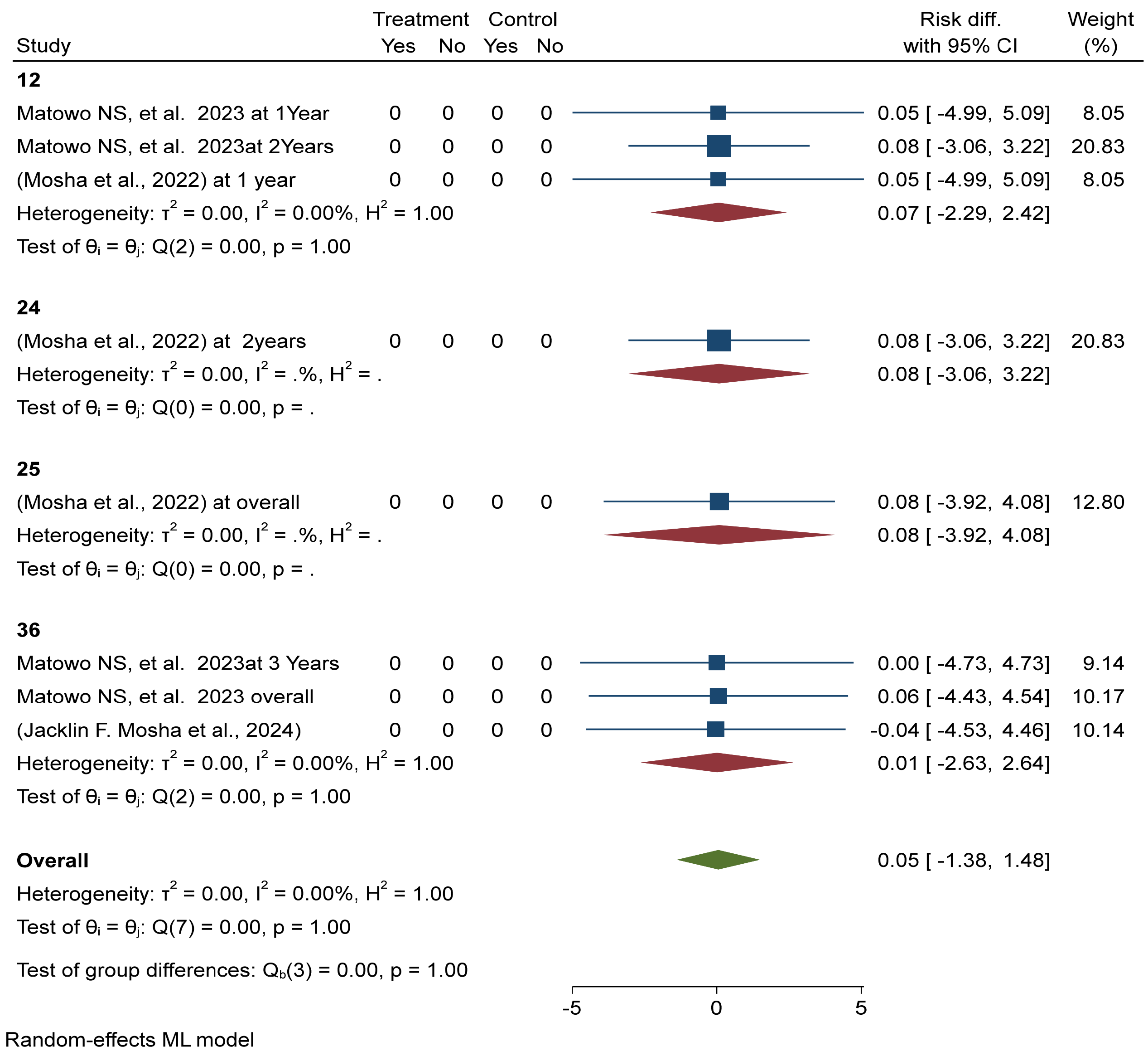

Figure S103: Forest plot shows subgroup analysis of post-intervention follow-up effectiveness and efficacy of Piperonyl butoxide versus pyriproxyfen long-lasting insecticidal nets (LLINs) reduce pooled mean entomological inoculation rate per household per night in Africa 2024

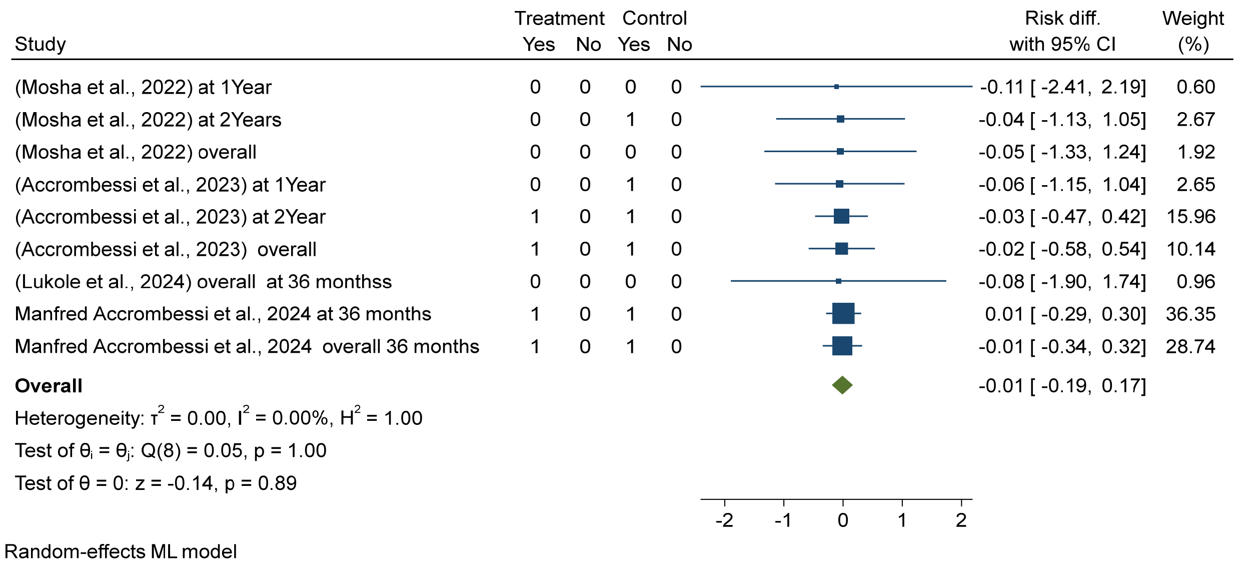

Figure S104: Forest plot shows post-intervention effectiveness and efficacy of chlorfenapyr long-lasting insecticidal nets (LLINs) in malaria case incidence reduction in children aged 6 months to 10 years over different durations in Africa 2024.

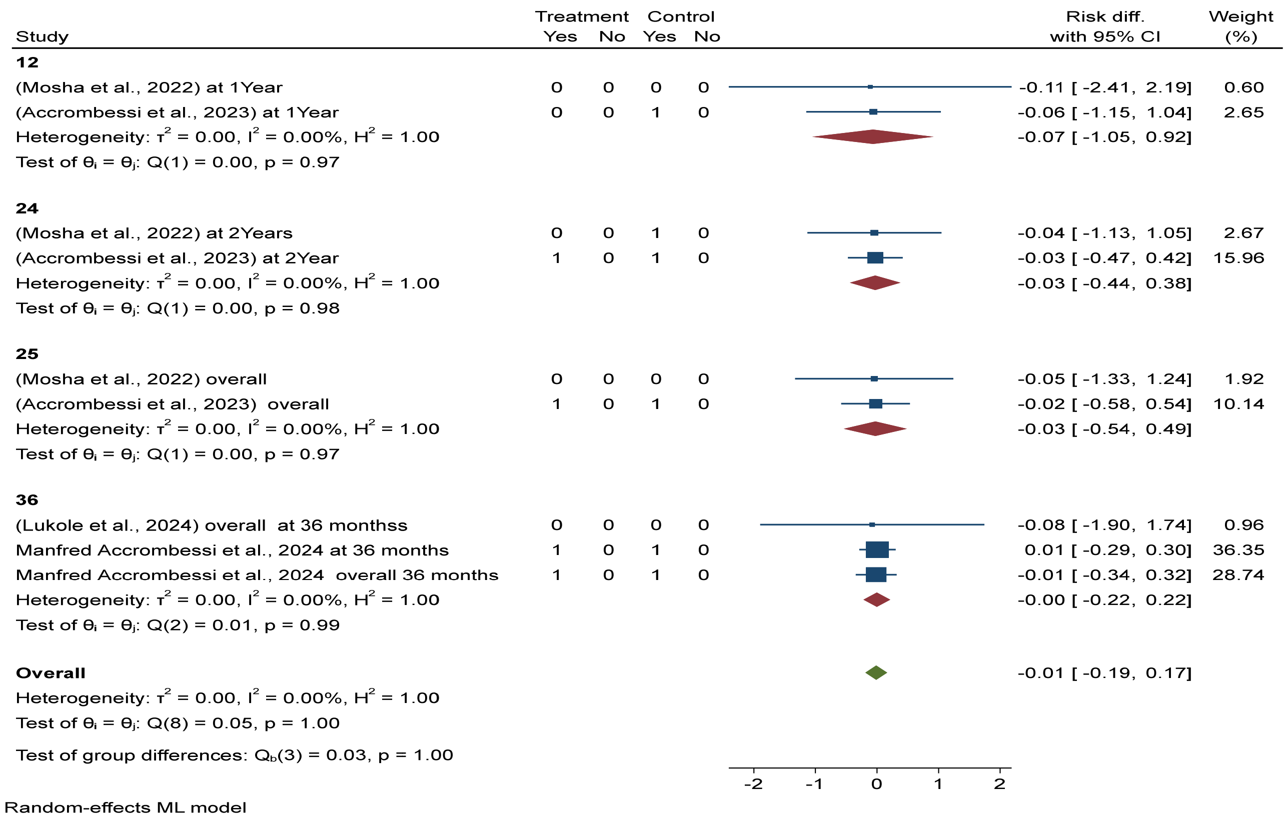

Figure S105: Forest plot shows subgroup analysis of post-intervention follow-up effectiveness and efficacy of chlorfenapyr long-lasting insecticidal nets (LLINs) in malaria case incidence reduction in children aged 6 months to 10 years over different durations in Africa 2024.

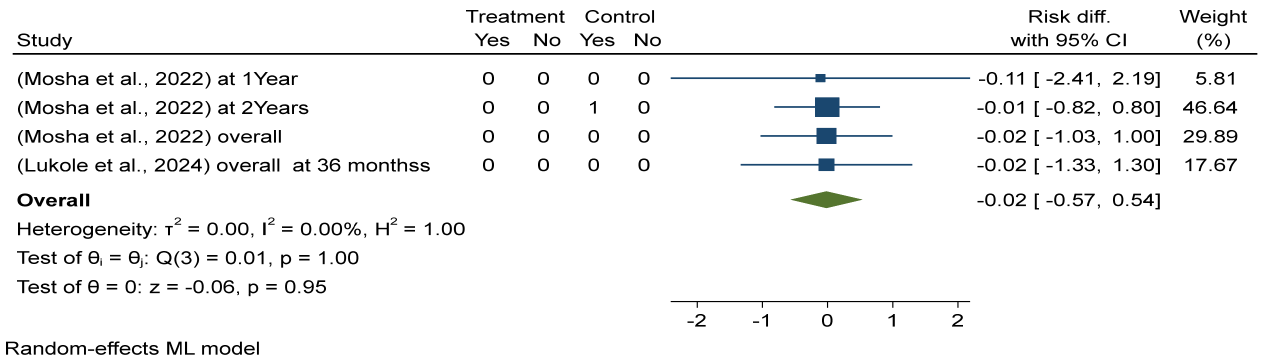

Figure S106: Forest plot shows post-intervention follow-up effectiveness and efficacy of piperonyl butoxide long-lasting insecticidal nets (LLINs) in malaria case incidence reduction in children aged 6 months to 10 years over different durations in Africa 2024.
