# Supplementary material for: Effectiveness and Efficacy of Long-Lasting Insecticidal Nets for Malaria Control in Africa: Systematic Review and Meta-Analysis of Randomized Controlled Trials
Source: Int J Environ Res Public Health. 2025 Jun 30;22(7):1045. doi: 10.3390/ijerph22071045 (PMC12294781; doi:10.3390/ijerph22071045)
Supplement: Supplementary file 1 [file ijerph-22-01045-s001.zip › File S4. Evidence grade supplementary 3.pdf]

The adopted GRADE (Grading of Recommendations Assessment, Development and Evaluation) as the method for assessing the quality of a body of evidence and for determining the direction and strength of the resulting recommendations.
This generated evidence was evaluated the effectiveness and efficacy of pyriproxyfen, chlorfenapyr, and piperonyl butoxide long-lasting insecticidal nets against the pyrethroid-only LLINs.

This study found that PYR-only LLINs (control arm) had higher pooled prevalence of malaria infection, case incidence, anemia, mean indoor vector density, incubation rate, and sporozoite rate as compared to intervention group (PPF, CFP, and PBO LLINs

Table 1. Evidence profile compared the effectiveness and efficacy of pyriproxyfen, chlorfenapyr, and piperonyl butoxide LLINs with pyrethroid-only LLINs for malaria control in Africa

**People**	All age, adult or mixed (children and adult) included studies				
**Settings**	Africa				
**Intervention**	Effectiveness or efficacy of long-lasting insecticidal nets (LLINs) of pyriproxyfen, chlorfenapyr, and piperonyl butoxide				
**Comparison**	Pyrethroid-only long-lasting insecticidal nets (LLINs)				
Outcomes	Pyriproxyfen LLINs	Piperonyl butoxide LLINs	Chlorfenapyr LLINs	**Pyrethroid-only long-lasting insecticidal nets (LLINs)**	Certainty of the evidence (GRADE)
Malaria infection (10)	Pooled prevalence<br>33.70 per 100 children<br>(95% CI: 28.03–39.37%)	Pooled prevalence<br>32.38 per 100 children<br>(95% CI: 25.27–39.50%)	Pooled prevalence<br>25.58 per 100 children<br>(95% CI: 19.52–31.64%)	40.84Per 100 children<br>(32.45%, 49.22%)	⊕⊕⊕⊕<br>**High:**
Anemia (8)	Pooled prevalence<br>29.28per 100 children<br>(95% CI: 5.81–52.75%)	Pooled prevalence<br>14.31 per 100 children<br>(95% CI: 6.11%, 22.52%)	Pooled prevalence<br>29.28per 100 children<br>(95% CI: 5.81–52.75%)	25.18 Per 100 children<br>(12.78%, 37.58%)	⊕⊕⊕⊕<br>**High:**
Malaria case incidence per children years(4)	Pooled malaria case incidence<br>69 per 100 children years<br>(95% CI: 0.46, 0.89)	Pooled malaria case incidence<br>31per 100 children years<br>(95% CI: 0.19, 0.43)	Pooled malaria case incidence<br>46 per 100 children years<br>(95% CI: 0.28 , 0.63)	46 Per 100 children years<br>(0.28 , 0.63)	⊕⊕⊕⊕<br>**High:**

Mean indoor vectors/ vector density per household per night (8)	Pooled mean indoor vectors density 7.74 per 100 household (95% CI: 4.71, 10.78%)	Pooled mean indoor vectors density 1.9per 100 household per night (95% CI: 1.15, 2.66%)	Pooled mean indoor vectors density 5.53per 100 household per night (95% CI: 2.82, 8.15%)	8.04 Per 100 Household (4.28%, 11.81%)	⊕⊕⊕⊕ **High:**
Mean entomological inoculation rate per household per night (6)	Pooled mean Incubation Rate 4per 100 household (95% CI: (-0.00, 0.08%)	Pooled mean MEIR 3per 100 household per night (95% CI: 0.00, 0.06%)	Pooled mean Incubation rate 4per 100 household (95% CI: (-0.00, 0.08%)	7Per 100 Household (0.03, 0.12%)	⊕⊕⊕⊕ **High:**
Sporozoite rate per mosquitoes (7).	Pooled sporozoite rate 165per 100 anopheles (95% CI: 1.13 2.18%)	Pooled sporozoite rate 172 per 100 anopheles (95% CI: 1.06, 2.38%)	Pooled sporozoite rate 79 per 100 anopheles (95% CI: 0.49, 1.09 %)	227 Per 100 anopheles (1.59 2.95%)	⊕⊕⊕⊕ **High:**
Margin of error = Confidence interval (95% CI) RR: Risk ratio GRADE: GRADE Working Group grades of evidence (see above and last page)					

The evidence generated from this meta-analysis reveals that pyriproxyfen (PPF) long-lasting insecticidal nets (LLINs) have no significant difference in malaria infection, case incidence, or anemia reduction among children as compared to pyrethroid-only LLINs. However, this study found that Pyriproxyfen (PPF) LLINs effectively and efficaciously reduce indoor vector density, entomological inoculation rate, and sporozoite rate of malaria parasites compared to pyrethroid-only LLINs.

The study found that chlorfenapyr(CFP) and piperonyl butoxide (PBO) long-lasting insecticidal nets (LLINs) are highly effective and efficacious in reducing malaria infection, case incidence, and anemia among children, as well as reducing indoor vector density, incubation rate, and sporozoite rate in Africa as compared to pyrethroid-only LLINs.

Critical appraisal of individual randomized control trials revealed that 100% of the studies scored high quality, and Cochrane methodology was used to assess the risk of bias and evaluate evidence quality, which was graded as high. This research provides a very good indication of the likely effect. The likelihood that the effect will be substantially different is low.

Table 1. Evidence profile compared the effectiveness and efficacy of pyriproxyfen, chlorfenapyr, and piperonyl butoxide LLINs with pyrethroid-only LLINs for malaria control in Africa

**People**	**All age, adult or mixed (children and adult) included studies**			
**Settings**	**Africa**			
**Intervention**	**Effectiveness or efficacy of long-lasting insecticidal nets (LLINs) of pyriproxyfen, chlorfenapyr, and piperonyl butoxide**			
**Comparison**	**Pyrethroid-only long-lasting insecticidal nets (LLINs)**			
Outcomes	Pyriproxyfen LLINs<br>Relative effect (95% CI)	piperonyl butoxide LLINs<br>Relative effect (95% CI)	Chlorfenapyr LLINs<br>Relative effect (95% CI)	Certainty of the evidence (GRADE)
**Malaria infection (9)**	**0.0% no difference (-0.03,0.02%)**	**1% less (-0.02, 0.01%)**	**-1% less (-0.04 to 0.02%)**	⊕⊕⊕⊕ **High:**
**Anemia (8),**	**0.0% no difference (-0.05 to 0.05%)**	**2% less (-0.07 to 0.04%)**	**1% less (-0.05 to 0.03%)**	⊕⊕⊕⊕ **High:**
**Malaria case incidence per children years (4)**	**0.0% no difference (-0.11 to 0.12%)**	**-3% less (-0.57, 0.5%)**	**-4% less (-0.33, 0.26%)**	⊕⊕⊕⊕ **High:**
**Mean indoor vectors/ vector density per household per night (8)**	**-1% less (-0.05, 0.08%)**	**-3% less (-0.19 to 0.13%)**	**-4% less (-0.15 to 0.06%)**	⊕⊕⊕⊕ **High:**
**Mean entomological incubation rate per household per night (6)**	**-7% less (-1.00 to 0.85%)**	**-12% less (-0.97, 0.73)**	**-23% less (-1.16 to 0.70%)**	⊕⊕⊕⊕ **High:**
**Sporozoite rate per mosquitoes (7).**	**15% less (--0.08, 0.37%)**	**10% less (-0.09, 0.29%)**	**9% less (--0.16, 0.35%)**	⊕⊕⊕⊕ **High:**
**Margin of error = Confidence interval (95% CI) RR: Risk ratio GRADE: GRADE Working Group grades of evidence (see above and last page)**				

This study also evaluated the effectiveness and efficacy of chlorfenapyr, and piperonyl butoxide long-lasting insecticidal nets compared to pyriproxyfen LLINs.

The evidence generated found that piperonyl butoxide (PBO) long-lasting insecticidal nets effectively and efficaciously reduce indoor vector density, entomological incubation rate, and sporozoite rate of malaria parasites compared to Pyriproxyfen (PPF) LLINs, but no significant difference was found in malaria infection reduction among children who use piperonyl butoxide (PBO) versus Pyriproxyfen (PPF) long-lasting insecticidal nets in Africa.

The study found that chlorfenapyr (CFP) long-lasting insecticidal nets (LLINs) are highly effective and superiorly efficacious in reducing malaria infection, and case incidence among children, as well as reducing mean indoor vector density, mean entomological incubation rate, and sporozoite rate compared to pyriproxyfen (PPF) long-lasting insecticidal nets (LLINs) in Africa.

Critical appraisal of individual randomized control trials revealed that 100% of the studies scored high quality, and Cochrane methodology was used to assess the risk of bias and evaluate evidence quality, which was graded as high. This research provides a very good indication of the likely effect. The likelihood that the effect will be substantially different is low.

Table 1.Evidence profile compared the effectiveness and efficacy of chlorfenapyr, and piperonyl butoxide long-lasting insecticidal nets with pyriproxyfen long-lasting insecticidal nets for malaria control in Africa

**People**	**All age, adult or mixed (children and adult) included studies**		
**Settings**	**Africa**		
**Intervention**	**Effectiveness or efficacy of long-lasting insecticidal nets (LLINs) of chlorfenapyr, and piperonyl butoxide**		
**Comparison**	**Pyriproxyfen long-lasting insecticidal nets (LLINs)**		
Outcomes	piperonyl butoxide LLINs<br>Relative effect (95% CI)	Chlorfenapyr LLINs<br>Relative effect (95% CI)	Certainty of the evidence (GRADE)
**Malaria infection (9)**	**0.0% no difference<br>(-0.04,0.04%)**	**-1% less<br>(-0.04 to 0.03%)**	⊕⊕⊕⊕<br>**High:**
**Malaria case incidence per children years (4)**	**-2% less<br>(-0.57, 0.54%)**	**-1% less<br>(-0.19, 0.17%)**	⊕⊕⊕⊕<br>**High:**
**Mean indoor vector density per household per night (8)**	**-4% less<br>(-0.24 to 0.16%)**	**-1% less<br>(-0.08 to 0.06%)**	⊕⊕⊕⊕<br>**High:**
**Mean entomological incubation rate per household per night (6)**	**-5% less<br>(-1.38, 1.48)**	**-15% less<br>(--1.18, 0.88%)**	⊕⊕⊕⊕<br>**High:**
**Sporozoite rate per mosquitoes (7).**	**-1 less<br>(-0.28, 0.26%)**	**-7% less<br>(-0.35, 0.21%)**	⊕⊕⊕⊕<br>**High:**

Margin of error = Confidence interval (95% CI) RR: Risk ratio GRADE: GRADE Working Group grades of evidence (see above and last page)

Additional

Table 1: GRADE evidence profile of the effectiveness and efficacy of pyriproxyfen long-lasting insecticidal nets (LLINs) compared with pyrethroid-only LLINs for malaria control in Africa from a systematic review and meta-analysis of randomized controlled trials by Demissie DB. et al.

Quality assessment						Summary of findings		
No of studies (No of participants)/households	Study limitations	Consistency	Directness	Precision	Publication bias	Relative effect† (95% CI)	Absolute effect (95% CI)	Quality
Malaria infection reduction among children								
10(34327)	No serious limitations	No important inconsistency	Direct	No important imprecision	Unlikely	0.0% ‡ no difference (-0.03,0.02%)	33.70 per 100 children vs 40.84Per 100 children	++++ High
Anemia prevalence among children								
9 (65235)	No serious limitations	No important inconsistency	Direct	No important imprecision	Unlikely	0.0% ‡ no difference (-0.05 to 0.05%)	29.28per 100 children vs 25.18 Per 100 children	++++ High
Malaria case incidence per children years								
4 (1484)	No serious limitations	No important inconsistency	Direct	No important imprecision	Unlikely	0.0% ‡ no difference (-0.11 to 0.12%)	69 per 100 children years Vs 46 Per 100 children years	++++ High
Mean indoor vectors/ vector density per household per night								
8 (18074)	No serious limitations	No important inconsistency	Direct	No important imprecision	Unlikely	-1% less (-0.05, 0.08%)	7.74 per 100 household Vs 8.04 Per 100 Household	++++ High
Mean entomological inoculation rate per household per night								
3 (8640)	No serious limitations	No important inconsistency	Direct	No important imprecision	Unlikely	-7% less (-1.00 to 0.85%)	4per 100 household per night vs 7Per 100 Household per night	++++ High
Sporozoite rate per mosquitoes								
3 (6192)	No serious limitations	No important inconsistency	Direct	No important imprecision	Unlikely	15% less (-0.08, 0.37%)	165per 100 anopheles vs 227 Per 100 anopheles	++++ High

* All studies, patients blinded, outcome assessors blinded in all studies, analyzed using intention to treat, and per per-protocol.

†Relative risks (95% confidence intervals) are based on random effect models.

‡Confidence interval includes possible benefit from either long-lasting insecticidal nets (LLINs) or no difference impacts in malaria control.

Factors in deciding on quality of evidence

Factors that might decrease quality of evidence

- Study limitations
- Inconsistency of results
- Indirectness of evidence
- Imprecision
- Publication bias
- Factors that might increase quality of evidence
- Large magnitude of effect
- Plausible confounding, which would reduce a demonstrated effect
- Dose-response gradient

Table 1: GRADE evidence profile of the effectiveness and efficacy of piperonyl butoxide long-lasting insecticidal nets (LLINs) compared with pyrethroid-only LLINs for malaria control in Africa from a systematic review and meta-analysis of randomized controlled trials by Demissie DB. et al.

Quality assessment						Summary of findings		
No of studies (No of participants)/households	Study limitations	Consistency	Directness	Precision	Publication bias	Relative effect† (95% CI)	Absolute effect (95% CI)	Quality
Malaria infection reduction among children								
10(34327)	No serious limitations	No important inconsistency	Direct	No important imprecision	Unlikely	1% less (-0.02, 0.01%)	32.38 per 100 children vs 40.84Per 100 children	++++ High
Anemia prevalence among children								
9 (65235)	No serious limitations	No important inconsistency	Direct	No important imprecision	Unlikely	2% less (-0.07 to 0.04%)	14.31 per 100 children vs 25.18 Per 100 children	++++ High
Malaria case incidence per children years								
4 (1484)	No serious limitations	No important inconsistency	Direct	No important imprecision	Unlikely	-3% less (-0.57, 0.5%)	31 per 100 children years Vs 46 Per 100 children years	++++ High
Mean indoor vectors/ vector density per household per night								
8 (18074)	No serious limitations	No important inconsistency	Direct	No important imprecision	Unlikely	-3% less (-0.19 to 0.13%)	1.9 per 100 household Vs 8.04 Per 100 Household	++++ High
Mean entomological inoculation rate per household per night								
3 (8640)	No serious limitations	No important inconsistency	Direct	No important imprecision	Unlikely	-12% less (-0.97, 0.73)	3per 100 household per night vs 7Per 100 Household per night	++++ High
Sporozoite rate per mosquitoes								
3 (6192)	No serious limitations	No important inconsistency	Direct	No important imprecision	Unlikely	10% less (-0.09, 0.29%)	172per 100 anopheles vs 227 Per 100 anopheles	++++ High

* All studies, patients blinded, outcome assessors blinded in all studies, analyzed using intention to treat, and per per-protocol.

†Relative risks (95% confidence intervals) are based on random effect models.

‡Confidence interval includes possible benefit from either long-lasting insecticidal nets (LLINs) or no difference impacts in malaria control.

Table 1: GRADE evidence profile of the effectiveness and efficacy of chlorfenapyr long-lasting insecticidal nets (LLINs) compared with pyrethroid-only LLINs for malaria control in Africa from a systematic review and meta-analysis of randomized controlled trials by Demissie DB. et al.

Quality assessment						Summary of findings		
No of studies (No of participants)/households	Study limitations	Consistency	Directness	Precision	Publication bias	Relative effect† (95% CI)	Absolute effect (95% CI)	Quality
Malaria infection reduction among children								
10(34327)	No serious limitations	No important inconsistency	Direct	No important imprecision	Unlikely	-1% less (-0.04 to 0.02%)	25.58 per 100 children vs 40.84Per 100 children	++++ High
Anemia prevalence among children								
9 (65235)	No serious limitations	No important inconsistency	Direct	No important imprecision	Unlikely	1% less (-0.05 to 0.03%)	29.28 per 100 children vs 25.18 Per 100 children	++++ High
Malaria case incidence per children years								
4 (1484)	No serious limitations	No important inconsistency	Direct	No important imprecision	Unlikely	-4% less (-0.33, 0.26%)	46per 100 children years Vs 46 Per 100 children years	++++ High
Mean indoor vectors/ vector density per household per night								
8 (18074)	No serious limitations	No important inconsistency	Direct	No important imprecision	Unlikely	-4% less (-0.15 to 0.06%)	5.53 per 100 household Vs 8.04 Per 100 Household	++++ High
Mean entomological inoculation rate per household per night								
3 (8640)	No serious limitations	No important inconsistency	Direct	No important imprecision	Unlikely	-23% less (-1.16 to 0.70%)	4per 100 household per night vs 7Per 100 Household per night	++++ High
Sporozoite rate per mosquitoes								
3 (6192)	No serious limitations	No important inconsistency	Direct	No important imprecision	Unlikely	9% less (-0.16, 0.35%)	79per 100 anopheles vs 227 Per 100 anopheles	++++ High

* All studies, patients blinded, outcome assessors blinded in all studies, analyzed using intention to treat, and per per-protocol.

†Relative risks (95% confidence intervals) are based on random effect models.

‡Confidence interval includes possible benefit from either long-lasting insecticidal nets (LLINs) or no difference impacts in malaria control.

Table 1: GRADE evidence profile of the effectiveness and efficacy of piperonyl butoxide long-lasting insecticidal nets (LLINs) compared with pyriproxyfen long-lasting insecticidal nets for malaria control in Africa, from a systematic review and meta-analysis of randomized controlled trials by Demissie DB. et al.

Quality assessment						Summary of findings		
No of studies (No of participants)/households	Study limitations	Consistency	Directness	Precision	Publication bias	Relative effect† (95% CI)	Absolute effect (95% CI)	Quality
Malaria infection reduction among children								
10(34327)	No serious limitations	No important inconsistency	Direct	No important imprecision	Unlikely	0.0% ‡ no difference (-0.04,0.04%)	32,28 per 100 children vs 33.70 Per 100 children	++++ High
Anemia prevalence among children								
9 (65235)	No serious limitations	No important inconsistency	Direct	No important imprecision	Unlikely	--------	14.31 per 100 children vs 29.28 Per 100 children	++++ High
Malaria case incidence per children years								
4 (1484)	No serious limitations	No important inconsistency	Direct	No important imprecision	Unlikely	-2% less (-0.57, 0.54%)	31 per 100 children years Vs 69Per 100 children years	++++ High
Mean indoor vectors/ vector density per household per night								
8 (18074)	No serious limitations	No important inconsistency	Direct	No important imprecision	Unlikely	-4% less (-0.24 to 0.16%)	1.9 per 100 household Vs 7,74Per 100 Household	++++ High
Mean entomological inoculation rate per household per night								
3 (8640)	No serious limitations	No important inconsistency	Direct	No important imprecision	Unlikely	-5% less (-1.38, 1.48)	3per 100 household per night vs 4Per 100 Household per night	++++ High
Sporozoite rate per mosquitoes								
3 (6192)	No serious limitations	No important inconsistency	Direct	No important imprecision	Unlikely	-1 less (-0.28, 0.26%)	172 per 100 anopheles vs 165Per 100 anopheles	++++ High

* All studies, patients blinded, outcome assessors blinded in all studies, analyzed using intention to treat, and per per-protocol.

†Relative risks (95% confidence intervals) are based on random effect models.

‡Confidence interval includes possible benefit from either long-lasting insecticidal nets (LLINs) or no difference impacts in malaria control.

Table 1: GRADE evidence profile of the effectiveness and efficacy of chlorfenapyr long-lasting insecticidal nets (LLINs) compared with pyriproxyfen long-lasting insecticidal nets for malaria control in Africa, from a systematic review and meta-analysis of randomized controlled trials by Demissie DB. et al.

Quality assessment						Summary of findings		
No of studies (No of participants)/households	Study limitations	Consistency	Directness	Precision	Publication bias	Relative effect† (95% CI)	Absolute effect (95% CI)	Quality
Malaria infection reduction among children								
10(34327)	No serious limitations	No important inconsistency	Direct	No important imprecision	Unlikely	-1% less (-0.04 to 0.03%)	25.58 per 100 children vs 33.70 Per 100 children	++++ High
Anemia prevalence among children								
9 (65235)	No serious limitations	No important inconsistency	Direct	No important imprecision	Unlikely	--------	29.28 per 100 children vs 29.28 Per 100 children	++++ High
Malaria case incidence per children years								
4 (1484)	No serious limitations	No important inconsistency	Direct	No important imprecision	Unlikely	--1% less (-0.19, 0.17%)	46 per 100 children years Vs 69Per 100 children years	++++ High
Mean indoor vectors/ vector density per household per night								
8 (18074)	No serious limitations	No important inconsistency	Direct	No important imprecision	Unlikely	-1% less (-0.08 to 0.06%)	5.53 per 100 household Vs 7.74Per 100 Household	++++ High
Mean entomological inoculation rate per household per night								
3 (8640)	No serious limitations	No important inconsistency	Direct	No important imprecision	Unlikely	–15% less (–1.18, 0.88%)	4per 100 household per night vs 4Per 100 Household per night	++++ High
Sporozoite rate per mosquitoes								
3 (6192)	No serious limitations	No important inconsistency	Direct	No important imprecision	Unlikely	-7% less (-0.35, 0.21%)	79per 100 anopheles vs 165Per 100 anopheles	++++ High

* All studies, patients blinded, outcome assessors blinded in all studies, analyzed using intention to treat, and per per-protocol.

†Relative risks (95% confidence intervals) are based on random effect models.

‡Confidence interval includes possible benefit from either long-lasting insecticidal nets (LLINs) or no difference impacts in malaria control.
