# Supplementary material for: Effectiveness and Efficacy of Long-Lasting Insecticidal Nets for Malaria Control in Africa: Systematic Review and Meta-Analysis of Randomized Controlled Trials
Source: Int J Environ Res Public Health. 2025 Jun 30;22(7):1045. doi: 10.3390/ijerph22071045 (PMC12294781; doi:10.3390/ijerph22071045)
Supplement: Supplementary file 1 [file ijerph-22-01045-s001.zip › File S5. Supplementary details table 4.pdf]

JBI critical appraisal tool for randomized controlled trials and | GRADE evidence profile for Malaria infection prevalence in selected children using different long-lasting insecticidal nets (LLINs) as malaria control in Africa

								Households and children (aged 6 months to 14 years) selected for baseline cross-sectional survey							
Author year	Country	study design	No of cluster	Interventions and Control	Population in core and buffer areas	Population in core area of clusters	Households in the lowest socioeconomic	Number selected HH	Number selected Children	LLIN use of all age groups	LLIN use in selected children	Malaria infection children	SE	Overall quality result	Overall evidence (GRADE)
(Mosha et al., 2022)	Tanzania	RCT	84	Standard LLIN/Pyrethroid-only group	6183	43877	30.9	680	1295	59.6	64.2	45.9	1.911	High	**High:**
				Pyriproxyfen group	57567	43266	34.5	667	1290	62.2	65	46.2	1.930	High	**High:**
				Chlorfenapyr group	60115	41748	31.1	671	1249	59.3	62.5	42.7	1.910	High	**High:**
				Piperonyl butoxide group	57631	45020	37.1	638	1185	61.7	63.4	42	1.954	High	**High:**
(Protopopoff et al., 2018)	Tanzania	RCT	48	Standard LLIN/Pyrethroid-only group	33820	15947	31	464	1085	30	39	68	2.166	High	**High:**
				Piperonyl butoxide group/ PBO LLIN	32861	16282	31	534	1086	26	32	61	2.111	High	**High:**
				Standard LLIN plus IRS	38081	16358	38	528	1092	28	31	67	2.046	High	**High:**
				PBO LLIN plus IRS	31138	14845	35	467	1066	26	32	64	2.221	High	**High:**
(Jacklin F. Mosha et al., 2024)	Tanzania	RCT	84	PY LLINs (reference)	61183	43877	28.9	394		59.6	63.2	59.6	2.472	High	**High:**
				Pyriproxyfen–PY LLINs	57567	43266	36.1	390		62.2	64.9	46.2	2.525	High	**High:**
				Chlorfenapyr–PY LLINs	60115	41748	30.7	426		59.3	62.5	42.7	2.397	High	**High:**
				PBO–PY LLINs	57631	45020	36.6	333		61.7	63.4	42	2.705	High	**High:**
(Protopopoff et al., 2023)	Tanzania	RCT	48	Standard LLIN/Pyrethroid-only group	33,820	15,947	31.5	464		30.1		67.8	2.169	High	**High:**
				Piperonyl butoxide group/ PBO LLIN	32,861	16,282	31.1	534		26.3		61.1	2.110	High	**High:**
				Standard LLIN plus IRS	38,081	16,358	37.5	528		27.6		66.6	2.053	High	**High:**
				PBO LLIN plus IRS	31,138	14,845	34.9	467		26.3		63.6	2.226	High	**High:**
(Accrombessi et al., 2023)	Benin	RCT	60	Pyrethroid-only LLIN group	69239	1058	29	1487		96.5	99.2	46.5	1.293	High	**High:**
				Pyriproxyfen-pyrethroid LLIN group	74822	1096	35.8	1479		95.8	99	43.1	1.288	High	**High:**
				Chlorfenapyr-pyrethroid LLIN group	70989	1121	36.2	1474		94.9	98.7	40.7	1.280	High	**High:**
Maiteki-Sebuguzi et al., 2023	Uganda	RCT	48	Non-PBO LLIN	2399		32.9	473	3973	95		17.8	1.759	High	**High:**
				PBO LLIN	2098		36.3	414	3676	91		24.3	2.108	High	**High:**
(Staedke et al., 2020)	Uganda	RCT	52	Non-PBO LLIN	2598		33	17	4328	64		19	9.515	High	**High:**
				PBO LLIN	2598		34	20	4506	66		19	8.772	High	**High:**
(Minakawa et al., 2021)	Kenya	RCT	8	piperonyl butoxide (PBO-LLINs)	12,098			982	775	26	56	61	1.556	High	**High:**
				standard LLINs	3,352			1,028	861	24	54	59	1.534	High	**High:**
(Lukole et al., 2024)	Tanzania	RCT	21	Pyrethroid (PY) LLIN (n)			27.5	498	1464	2.5		40.9	2.203	High	**High:**
				Chlorfenapyr-PY LLIN (n)			27.5	557	1500	3.8		23.8	1.804	High	**High:**
				PBO-PY LLIN (n)			27.5	403	915	3.8		34.5	2.368	High	**High:**
				Pyriproxyfen-PY LLIN (n)			27.5	458	1181	3.8		35.3	2.233	High	**High:**
Manfred Accrombessi et al., 2024	Benin	RCT	60	Pyrethroid-only LLIN group	69239	69239	29	1488	600	95.1	96.5	46.5	1.293	High	**High:**
				Pyriproxyfen-pyrethroid LLIN group	74822	74822	35.8	1479	600	96.4	95.8	43.1	1.288	High	**High:**
				Chlorfenapyr-pyrethroid LLIN group	70989	70989	36.2	1474	600	97.1	94.9	40.7	1.280	High	**High:**
Continent	Africa	Total	513		1145035	693011		21916	34327	51.925	66.86	46.3			

JBI critical appraisal tool for randomized controlled trials and | GRADE evidence profile for Anaemia prevalence in children aged 6 months to 4 years using different long-lasting insecticidal nets (LLINs) as malaria control in Africa

					Study clusters Population			Households and children (aged 6 months to 14 years)							
Author year	Country	study design	No of cluster	Interventions and Control	Population in core and buffer areas	Population in core area of clusters	Households in the lowest socioeconomic	Number selected Households	Number selected Children	LLIN use of all age groups	LLIN use In selected children	Anaemia† prevalence in children	SE anemia	Overall quality result	Overall evidence (GRADE)
(Mosha et al., 2022)	Tanzania	RCT	84	Standard LLIN/Pyrethroid-only group	6183	43877	30.9	680	1295	59.6	64.2	6.2	0.670	High	**High:**
				Pyriproxyfen group	57567	43266	34.5	667	1290	62.2	65.0	5.6	0.640	High	**High:**
				Chlorfenapyr group	60115	41748	31.1	671	1249	59.3	62.5	5.5	0.645	High	**High:**
				Piperonyl butoxide group	57631	45020	37.1	638	1185	61.7	63.4	4.2	0.583	High	**High:**
(Protopopoff et al., 2018)	Tanzania	RCT	48	Standard LLIN/Pyrethroid-only group	33820	15947	31	464	1085	30.0	39.0	10.4	0.927	High	**High:**
				Piperonyl butoxide group/ PBO LLIN	32861	16282	31	534	1086	26.0	32.0	10.6	0.934	High	**High:**
				Standard LLIN plus IRS	38081	16358	38	528	1092	28.0	31.0	10.6	0.932	High	**High:**
				PBO LLIN plus IRS	31138	14845	35	467	1066	26.0	32.0	10.6	0.943	High	**High:**
(Jacklin F. Mosha et al., 2024)	Tanzania	RCT	84	PY LLINs (reference)	61183	43877	28.9	394	8329	59.6	63.2	6.1	0.262	High	**High:**
				Pyriproxyfen–PY LLINs	57567	43266	36.1	390	8329	62.2	64.9	5.2	0.243	High	**High:**
				Chlorfenapyr–PY LLINs	60115	41748	30.7	426	8329	59.3	62.5	5.5	0.250	High	**High:**
				PBO–PY LLINs	57631	45020	36.6	333	8329	61.7	63.4	4.2	0.220	High	**High:**
(Accrombessi et al., 2023)	Benin	RCT	60	Pyrethroid-only LLIN group	69239	1058	50.2	1487	884	96.5	99.2	50.2	1.682	High	**High:**
				Pyriproxyfen-pyrethroid LLIN group	74822	1096	35.8	1479	884	95.8	99.0	53.3	1.678	High	**High:**
				Chlorfenapyr-pyrethroid LLIN group	70989	1121	36.2	1474	884	94.9	98.7	53.4	1.678	High	**High:**
Maiteki-Sebuguzi et al., 2023	Uganda	RCT	48	Non-PBO LLIN	2399		32.9	473	3973			33.3	0.748	High	**High:**
				PBO LLIN	2098		36.3	414	3676			28.7	0.746	High	**High:**
(Staedke et al., 2020)	Uganda	RCT	52	Non-PBO LLIN	2598		33	17	4328	64.0		35.0	0.725	High	**High:**
				PBO LLIN	2598		34	20	4506	66.0		28.0	0.669	High	**High:**
(Minakawa et al., 2021)	Kenya	RCT	8	piperonyl butoxide (PBO-LLINs)	12,098			982	775	26.0	56.0	10.2	1.087	High	**High:**
				standard LLINs	3,352			1,028	861	24.0	54.0	10.5	1.045	High	**High:**
Manfred Accrombessi et al., 2024	Benin	RCT	60	Pyrethroid-only LLIN group	69239	69239	29	1488	600	95.1	96.5	50.2	2.041	High	**High:**
				Pyriproxyfen-pyrethroid LLIN group	74822	74822	35.8	1479	600	96.4	95.8	53.3	2.037	High	**High:**
				Chlorfenapyr-pyrethroid LLIN group	70989	70989	36.2	1474	600	97.1	94.9	53.3	2.037	High	**High:**
	**Continent Africa**	Total	444	Total	1009135	629579	34.55909091	18007	65235	61.4	66.9	22.7			

JBI critical appraisal tool for randomized controlled trials and | GRADE evidence profile for mean indoor vectors density per household per night using different long-lasting insecticidal nets (LLINs) as malaria control in Africa

					Study clusters Population			Households and children (aged 6 months to 14 years) selected for baseline cross-sectional survey				Entomological vector density			
Author year	Country	study design	No of cluster	Interventions and Control	Population in core and buffer areas	Population in core area of clusters	Households in the lowest socioeconomic	Number selected Households	Number selected Children	LLIN use of all age groups	LLIN use In selected children	Mean indoor vectors per household per night	SE	Overall quality result	Overall evidence (GRADE)
(Mosha et al., 2022)	Tanzania	RCT	84	Standard LLIN/Pyrethroid-only group	6183	43877	30.9	680	1295	59.6	64.2	5.9	0.9036	High	**High:**
				Pyriproxyfen group	57567	43266	34.5	667	1290	62.2	65.0	4.2	0.7767	High	**High:**
				Chlorfenapyr group	60115	41748	31.1	671	1249	59.3	62.5	2.8	0.6369	High	**High:**
				Piperonyl butoxide group	57631	45020	37.1	638	1185	61.7	63.4	1.9	0.5405	High	**High:**
(Protopopoff et al., 2018)	Tanzania	RCT	48	Standard LLIN/Pyrethroid-only group	33820	15947	31	464	1085	30.0	39.0	17.0	1.7438	High	**High:**
				Piperonyl butoxide group/ PBO LLIN	32861	16282	31	534	1086	26.0	32.0	37.0	2.0893	High	**High:**
				Standard LLIN plus IRS	38081	16358	38	528	1092	28.0	31.0	11.8	1.4040	High	**High:**
				PBO LLIN plus IRS	31138	14845	35	467	1066	26.0	32.0	43.6	2.2947	High	**High:**
(Jacklin F. Mosha et al., 2024)	Tanzania	RCT	84	PY LLINs (reference)	61183	43877	28.9	394	8329	59.6	63.2	5.9	1.1871	High	**High:**
				Pyriproxyfen–PY LLINs	57567	43266	36.1	390	8329	62.2	64.9	4.2	1.0157	High	**High:**
				Chlorfenapyr–PY LLINs	60115	41748	30.7	426	8329	59.3	62.5	2.8	0.7993	High	**High:**
				PBO–PY LLINs	57631	45020	36.6	333	8329	61.7	63.4	1.9	0.7482	High	**High:**
(Accrombessi et al., 2023)	Benin	RCT	60	Pyrethroid-only LLIN group	69239	1058	50.2	1487	884	96.5	99.2	17.0	0.9741	High	**High:**
				Pyriproxyfen-pyrethroid LLIN group	74822	1096	35.8	1479	884	95.8	99.0	37.0	1.2554	High	**High:**
				Chlorfenapyr-pyrethroid LLIN group	70989	1121	36.2	1474	884	94.9	98.7	11.8	0.8403	High	**High:**
Maiteki-Sebuguzi et al., 2023	Uganda	RCT	48	Non-PBO LLIN	2399		32.9	473	3973			43.6	2.2801	High	**High:**
				PBO LLIN	2098		36.3	414	3676			0.9	0.4641	High	**High:**
(Staedke et al., 2020)	Uganda	RCT	52	Non-PBO LLIN	2598		33	17	4328	64.0		0.6	1.9038	High	**High:**
				PBO LLIN	2598		34	20	4506	66.0		0.5	1.5455	High	**High:**
(Minakawa et al., 2021)	Kenya	RCT	8	piperonyl butoxide (PBO-LLINs)	12,098			982	775	26.0	56.0	0.3	0.1745	High	**High:**
				standard LLINs	3,352			1,028	861	24.0	54.0	0.5	0.2200	High	**High:**
(Staedke et al., 2020)	Uganda	RCT	52	Non-PBO LLIN	2598			33	17	19.0	64.0	0.3	0.9520	High	**High:**
				PBO LLIN	2598			34	20	19.0	66.0	0.4	1.0825	High	**High:**
Manfred Accrombessi et al., 2024	Benin	RCT	60	Pyrethroid-only LLIN group	69239	69239	29	1488	600	95.1	96.5	22.5	1.0825	High	**High:**
				Pyriproxyfen-pyrethroid LLIN group	74822	74822	35.8	1479	600	96.4	95.8	25.5	1.1334	High	**High:**
				Chlorfenapyr-pyrethroid LLIN group	70989	70989	36.2	1474	600	97.1	94.9	14.0	0.9038	High	**High:**
	**Continent Africa**	Total	496		1014331	629579	34.56	18074	65272	57.9	66.7	12.1			

JBI critical appraisal tool for randomized controlled trials and | GRADE evidence profile for sporozoite rate of vectors infected with malaria parasite														
Author year	Country	study design	No of cluster	Interventions and Control	Population in core and buffer areas	Population in core area of clusters	Households in the lowest socioeconomic	Number selected Households	Number selected Children	LLIN use of all age groups	LLIN use In selected children	Sporozoite rate	SE	Overall quality result	Overall evidence (GRADE)
(Mosha et al., 2022)	Tanzania	RCT	84	Standard LLIN/Pyrethroid-only group	6183	43877	30.9	680	1295	59.6	64.2	4.4	0.787	High	**High:**
				Pyriproxyfen group	57567	43266	34.5	667	1290	62.2	65	3.3	0.692	High	**High:**
				Chlorfenapyr group	60115	41748	31.1	671	1249	59.3	62.5	2.2	0.566	High	**High:**
				Piperonyl butoxide group	57631	45020	37.1	638	1185	61.7	63.4	3	0.675	High	**High:**
(Protopopoff et al., 2018)	Tanzania	RCT	48	Standard LLIN/Pyrethroid-only group	33820	15947	31	464	1085	30	39	5	1.012	High	**High:**
				Piperonyl butoxide group/ PBO LLIN	32861	16282	31	534	1086	26	32	5	0.943	High	**High:**
				Standard LLIN plus IRS	38081	16358	38	528	1092	28	31	5	0.948	High	**High:**
				PBO LLIN plus IRS	31138	14845	35	467	1066	26	32	3	0.789	High	**High:**
(Jacklin F. Mosha et al., 2024)	Tanzania	RCT	84	PY LLINs (reference)	61183	43877	28.9	394	8329	59.6	63.2	4.4	1.033	High	**High:**
				Pyriproxyfen–PY LLINs	57567	43266	36.1	390	8329	62.2	64.9	3.3	0.905	High	**High:**
				Chlorfenapyr–PY LLINs	60115	41748	30.7	426	8329	59.3	62.5	2.2	0.711	High	**High:**
				PBO–PY LLINs	57631	45020	36.6	333	8329	61.7	63.4	3	0.935	High	**High:**
	**Continent Africa**	Total	216		553892	411254	33.41	6192	42664	49.63	53.59	3.65			

**JBI critical appraisal tool for randomized controlled trials and | GRADE evidence profile for Mean entomological inoculation rate per household per night (MEIR)**															
					Study clusters Population			Households and children (aged 6 months to 14 years) selected for baseline cross-sectional survey							
Author year	Country	study design	No of cluster	Interventions and Control	Population in core and buffer areas	Population in core area of clusters	Households in the lowest socioeconomic	Number selected Households	Number selected Children	LLIN use of all age groups	LLIN use In selected children	Mean entomological inoculation rate	EIR SE	Overall quality result	Overall evidence (GRADE)
(Mosha et al., 2022)	Tanzania	RCT	84	Standard LLIN/Pyrethroid-only group	6183	43877	30.9	680	1295	59.6	64.2	0.35	0.2265	High	**High:**
				Pyriproxyfen group	57567	43266	34.5	667	1290	62.2	65.0	0.11	0.1283	High	**High:**
				Chlorfenapyr group	60115	41748	31.1	671	1249	59.3	62.5	0.04	0.0772	High	**High:**
				Piperonyl butoxide group	57631	45020	37.1	638	1185	61.7	63.4	0.07	0.1047	High	**High:**
(Jacklin F. Mosha et al., 2024)	Tanzania	RCT	84	PY LLINs (reference)	61183	43877	28.9	394	8329	59.6	63.2	0.35	0.2975	High	**High:**
				Pyriproxyfen–PY LLINs	57567	43266	36.1	390	8329	62.2	64.9	0.11	0.1679	High	**High:**
				Chlorfenapyr–PY LLINs	60115	41748	30.7	426	8329	59.3	62.5	0.04	0.0969	High	**High:**
				PBO–PY LLINs	57631	45020	36.6	333	8329	61.7	63.4	0.07	0.1449	High	**High:**
Manfred Accrombessi et al., 2024	Benin	RCT	60	Pyrethroid-only LLIN group	69239	69239	29.0	1488	600	95.1	96.5	0.96	0.2528	High	**High:**
				Pyriproxyfen-pyrethroid LLIN group	74822	74822	35.8	1479	600	96.4	95.8	0.62	0.2041	High	**High:**
				Chlorfenapyr-pyrethroid LLIN group	70989	70989	36.2	1474	600	97.1	94.9	0.48	0.1800	High	**High:**
	**Continent Africa**	Total	228		633042	562872	33.4	8640	40135	70.4	72.4	0.29			
