# Supplementary material for: Effectiveness and Efficacy of Long-Lasting Insecticidal Nets for Malaria Control in Africa: Systematic Review and Meta-Analysis of Randomized Controlled Trials
Source: Int J Environ Res Public Health. 2025 Jun 30;22(7):1045. doi: 10.3390/ijerph22071045 (PMC12294781; doi:10.3390/ijerph22071045)
Supplement: Supplementary file 1 [file ijerph-22-01045-s001.zip › File S6. Additional Supplemental JBI critical appraisal tool for RCT.pdf]

Additional Supplemental: JBI critical appraisal tool for randomized controlled trials

RoB Assessor: 3	Date of Appraisal: 20/02/2024	Record Number: 11
Study Author: Dereje Bayissa Demissie, Getahun Fetensa, Firew Tiruneh Tiyare	Study Title: Effectiveness and efficacy of long-lasting insecticidal nets for malaria control in Africa: Systematic review and meta-analysis of randomized controlled trials	Study Year: 2024

	Internal Validity	Choice - Comments/ Justification	Yes	No	Unclear	N/A
	**Bias related to selection and allocation**					
1	Was true randomization used for assignment of participants to treatment groups?		☒	☐	☐	☐
2	Was allocation to treatment groups concealed?		☒	☐	☐	☐
3	Were treatment groups similar at the baseline?		☒	☐	☐	☐
	**Bias related to administration of intervention/exposure**					
4	Were participants blind to treatment assignment?		☒	☐	☐	☐
5	Were those delivering the treatment blind to treatment assignment?		☒	☐	☐	☐
6	Were treatment groups treated identically other than the intervention of interest?		☒	☐	☐	☐
	**Bias related to assessment, detection and measurement of the outcome**					
7	Were outcome assessors blind to treatment assignment?		Yes	No	Unclear	N/A
	**Outcome 1:** Malaria infection prevalence reduction		☒	☐	☐	☐

			Yes	No	Unclear	N/A
	**Outcome 2:** Anemia prevalence reduction among children		☒	☐	☐	☐
	**Outcome 3:** Malaria case incidence reduction		☒	☐	☐	☐
	**Outcome 4:** Mean indoor vector density reduction		☒	☐	☐	☐
	**Outcome 5:** Mean entomological incubation rate reduction		☒	☐	☐	☐
	**Outcome 6:** Sporozoite rate reduction		☒	☐	☐	☐

8	**Were outcomes measured in the same way for treatment groups?**		**Yes**	**No**	**Unclear**	**N/A**
	**Outcome 1:** Malaria infection prevalence reduction		☒	☐	☐	☐
	**Outcome 2:** Anemia prevalence reduction among children		☒	☐	☐	☐
	**Outcome 3:** Malaria case incidence		☒	☐	☐	☐
	**Outcome 4:** Mean indoor vector density reduction		☒	☐	☐	☐
	**Outcome 5:** Mean entomological incubation rate reduction		☒	☐	☐	☐
	**Outcome 6:** Sporozoite rate reduction		☒	☐	☐	☐

9	**Were outcomes measured in a reliable way**		**Yes**	**No**	**Unclear**	**N/A**
	**Outcome 1:** Malaria infection prevalence reduction		☒	☐	☐	☐
	**Outcome 2:** Anemia prevalence reduction among children		☒	☐	☐	☐

**Outcome 3:** Malaria case incidence		☒	☐	☐	☐
**Outcome 4:** Mean indoor vector density reduction		☒	☐	☐	☐
**Outcome 5:** Mean entomological incubation rate reduction		☒	☐	☐	☐
**Outcome 6:** Sporozoite rate reduction		☒	☐	☐	☐

Bias related to participant retention

10	**Was follow-up complete and if not, were differences between groups in terms of their follow-up adequately described and analyzed?**					
	**Outcome 1** Malaria infection prevalence reduction		**Yes**	**No**	**Unclear**	**N/A**
	Result 1 Effectiveness and efficacy of Pyriproxyfen as compared to pyrethroid-only LLINs		☒	☐	☐	☐
	Result 2 **Effectiveness and efficacy of** Chlorfenapyr as compared to pyrethroid-only LLINs		☒	☐	☐	☐
	Result 3 **Effectiveness and efficacy of** Piperonyl Butoxide as compared to pyrethroid-only LLINs		☒	☐	☐	☐
	**Outcome 2: Anemia prevalence reduction among children**		**Yes**	**No**	**Unclear**	**N/A**
	Result 1 Effectiveness and efficacy of Pyriproxyfen as compared to pyrethroid-only LLINs		☒	☐	☐	☐
	Result 2 **Effectiveness and efficacy of** Chlorfenapyr as compared to pyrethroid-only LLINs		☒	☐	☐	☐
	Result 3 **Effectiveness and efficacy of** Piperonyl Butoxide as compared to pyrethroid-only LLINs		☒	☐	☐	☐
	**Outcome 3: Malaria case incidence reduction**		**Yes**	**No**	**Unclear**	**N/A**
	Result 1 Effectiveness and efficacy of Pyriproxyfen as compared to pyrethroid-only LLINs		☒	☐	☐	☐
	Result 2 **Effectiveness and efficacy of** Chlorfenapyr as compared to pyrethroid-only LLINs		☒	☐	☐	☐

		Yes	No	Unclear	N/A
Result 3 **Effectiveness and efficacy of** Piperonyl Butoxide as compared to pyrethroid-only LLINs		☒	☐	☐	☐
**Outcome 4: Mean indoor vector density reduction**		**Yes**	**No**	**Unclear**	**N/A**
Result 1 Effectiveness and efficacy of Pyriproxyfen as compared to pyrethroid-only LLINs		☒	☐	☐	☐
Result 2 **Effectiveness and efficacy of** Chlorfenapyr as compared to pyrethroid-only LLINs		☒	☐	☐	☐
Result 3 **Effectiveness and efficacy of** Piperonyl Butoxide as compared to pyrethroid-only LLINs		☒	☐	☐	☐
**Outcome 5:Mean entomological incubation rate reduction**		**Yes**	**No**	**Unclear**	**N/A**
Result 1 Effectiveness and efficacy of Pyriproxyfen as compared to pyrethroid-only LLINs		☒	☐	☐	☐
Result 2 **Effectiveness and efficacy of** Chlorfenapyr as compared to pyrethroid-only LLINs		☒	☐	☐	☐
Result 3 **Effectiveness and efficacy of** Piperonyl Butoxide as compared to pyrethroid-only LLINs		☒	☐	☐	☐
**Outcome 6: Sporozoite rate reduction**		**Yes**	**No**	**Unclear**	**N/A**
Result 1 Effectiveness and efficacy of Pyriproxyfen as compared to pyrethroid-only LLINs		☒	☐	☐	☐
Result 2 **Effectiveness and efficacy of** Chlorfenapyr as compared to pyrethroid-only LLINs		☒	☐	☐	☐
Result 3 **Effectiveness and efficacy of** Piperonyl Butoxide as compared to pyrethroid-only LLINs		☒	☐	☐	☐

Statistical Conclusion Validity

#						
11	**Were participants analyzed in the groups to which they were randomized?**					
	**Outcome 1**		**Yes**	**No**	**Unclear**	**N/A**
	Result 1 Effectiveness and efficacy of Pyriproxyfen as compared to pyrethroid-only LLINs		☒	☐	☐	☐
	Result 2 **Effectiveness and efficacy of** Chlorfenapyr as compared to pyrethroid-only LLINs		☒	☐	☐	☐

		Yes	No	Unclear	N/A
Result 3 **Effectiveness and efficacy of** Piperonyl Butoxide as compared to pyrethroid-only LLINs		☒	☐	☐	☐
**Outcome 2: Anemia prevalence reduction among children**		**Yes**	**No**	**Unclear**	**N/A**
Result 1 Effectiveness and efficacy of Pyriproxyfen as compared to pyrethroid-only LLINs		☒	☐	☐	☐
Result 2 **Effectiveness and efficacy of** Chlorfenapyr as compared to pyrethroid-only LLINs		☒	☐	☐	☐
Result 3 **Effectiveness and efficacy of** Piperonyl Butoxide as compared to pyrethroid-only LLINs		☒	☐	☐	☐
**Outcome 3: Malaria case incidence reduction**		**Yes**	**No**	**Unclear**	**N/A**
Result 1 Effectiveness and efficacy of Pyriproxyfen as compared to pyrethroid-only LLINs		☒	☐	☐	☐
Result 2 **Effectiveness and efficacy of** Chlorfenapyr as compared to pyrethroid-only LLINs		☒	☐	☐	☐
Result 3 **Effectiveness and efficacy of** Piperonyl Butoxide as compared to pyrethroid-only LLINs		☒	☐	☐	☐
**Outcome 4: Mean indoor vector density reduction**		**Yes**	**No**	**Unclear**	**N/A**
Result 1 Effectiveness and efficacy of Pyriproxyfen as compared to pyrethroid-only LLINs		☒	☐	☐	☐
Result 2 **Effectiveness and efficacy of** Chlorfenapyr as compared to pyrethroid-only LLINs		☒	☐	☐	☐
Result 3 **Effectiveness and efficacy of** Piperonyl Butoxide as compared to pyrethroid-only LLINs		☒	☐	☐	☐
**Outcome 5:Mean entomological incubation rate reduction**		**Yes**	**No**	**Unclear**	**N/A**
Result 1 Effectiveness and efficacy of Pyriproxyfen as compared to pyrethroid-only LLINs		☒	☐	☐	☐
Result 2 **Effectiveness and efficacy of** Chlorfenapyr as compared to pyrethroid-only LLINs		☒	☐	☐	☐

			Yes	No	Unclear	N/A
	Result 3 **Effectiveness and efficacy of** Piperonyl Butoxide as compared to pyrethroid-only LLINs		☒	☐	☐	☐
	**Outcome 6: Sporozoite rate reduction**		**Yes**	**No**	**Unclear**	**N/A**
	Result 1 Effectiveness and efficacy of Pyriproxyfen as compared to pyrethroid-only LLINs		☒	☐	☐	☐
	Result 2 **Effectiveness and efficacy of** Chlorfenapyr as compared to pyrethroid-only LLINs		☒	☐	☐	☐
	Result 3 **Effectiveness and efficacy of** Piperonyl Butoxide as compared to pyrethroid-only LLINs		☒	☐	☐	☐
12	**Was appropriate statistical analysis used?**					
	**Outcome 1**		**Yes**	**No**	**Unclear**	**N/A**
	Result 1 Effectiveness and efficacy of Pyriproxyfen as compared to pyrethroid-only LLINs		☒	☐	☐	☐
	Result 2 **Effectiveness and efficacy of** Chlorfenapyr as compared to pyrethroid-only LLINs		☒	☐	☐	☐
	Result 3 **Effectiveness and efficacy of** Piperonyl Butoxide as compared to pyrethroid-only LLINs		☒	☐	☐	☐
	**Outcome 2: Anemia prevalence reduction among children**		**Yes**	**No**	**Unclear**	**N/A**
	Result 1 Effectiveness and efficacy of Pyriproxyfen as compared to pyrethroid-only LLINs		☒	☐	☐	☐
	Result 2 **Effectiveness and efficacy of** Chlorfenapyr as compared to pyrethroid-only LLINs		☒	☐	☐	☐
	Result 3 **Effectiveness and efficacy of** Piperonyl Butoxide as compared to pyrethroid-only LLINs		☒	☐	☐	☐
	**Outcome 3: Malaria case incidence reduction**		**Yes**	**No**	**Unclear**	**N/A**
	Result 1 Effectiveness and efficacy of Pyriproxyfen as compared to pyrethroid-only LLINs		☒	☐	☐	☐
	Result 2 **Effectiveness and efficacy of** Chlorfenapyr as compared to pyrethroid-only LLINs		☒	☐	☐	☐
	Result 3 **Effectiveness and efficacy of** Piperonyl Butoxide as compared to pyrethroid-only LLINs		☒	☐	☐	☐

Outcome 4: Mean indoor vector density reduction		Yes	No	Unclear	N/A
Result 1 Effectiveness and efficacy of Pyriproxyfen as compared to pyrethroid-only LLINs		☒	☐	☐	☐
Result 2 **Effectiveness and efficacy of** Chlorfenapyr as compared to pyrethroid-only LLINs		☒	☐	☐	☐
Result 3 **Effectiveness and efficacy of** Piperonyl Butoxide as compared to pyrethroid-only LLINs		☒	☐	☐	☐
**Outcome 5:Mean entomological incubation rate reduction**		**Yes**	**No**	**Unclear**	**N/A**
Result 1 Effectiveness and efficacy of Pyriproxyfen as compared to pyrethroid-only LLINs		☒	☐	☐	☐
Result 2 **Effectiveness and efficacy of** Chlorfenapyr as compared to pyrethroid-only LLINs		☒	☐	☐	☐
Result 3 **Effectiveness and efficacy of** Piperonyl Butoxide as compared to pyrethroid-only LLINs		☒	☐	☐	☐
**Outcome 6: Sporozoite rate reduction**		**Yes**	**No**	**Unclear**	**N/A**
Result 1 Effectiveness and efficacy of Pyriproxyfen as compared to pyrethroid-only LLINs		☒	☐	☐	☐
Result 2 **Effectiveness and efficacy of** Chlorfenapyr as compared to pyrethroid-only LLINs		☒	☐	☐	☐
Result 3 **Effectiveness and efficacy of** Piperonyl Butoxide as compared to pyrethroid-only LLINs		☒	☐	☐	☐

			Yes	No	Unclear	N/A
13	**Was the trial design appropriate and any deviations from the standard RCT design (individual randomization, parallel groups) accounted for in the conduct and analysis of the trial?**		☒	☐	☐	☐

Overall appraisal: **Include:** ☒ **Exclude:** ☐ **Seek Further Info:** ☐

Comments: Critical appraisal of individual randomized control trials revealed that 100% of the studies scored high quality
